# Oxidative stress in the eye and its role in the pathophysiology of ocular diseases

**DOI:** 10.1016/j.redox.2023.102967

**Published:** 2023-11-18

**Authors:** Elsa Wilma Böhm, Francesco Buonfiglio, Anna Maria Voigt, Philipp Bachmann, Tarek Safi, Norbert Pfeiffer, Adrian Gericke

**Affiliations:** Department of Ophthalmology, University Medical Center, Johannes Gutenberg University Mainz, Langenbeckstrasse 1, 55131, Mainz, Germany

**Keywords:** Oxidative stress, Reactive oxygen species, Antioxidants, Eye, Ocular diseases, Pathophysiology

## Abstract

Oxidative stress occurs through an imbalance between the generation of reactive oxygen species (ROS) and the antioxidant defense mechanisms of cells. The eye is particularly exposed to oxidative stress because of its permanent exposure to light and due to several structures having high metabolic activities. The anterior part of the eye is highly exposed to ultraviolet (UV) radiation and possesses a complex antioxidant defense system to protect the retina from UV radiation. The posterior part of the eye exhibits high metabolic rates and oxygen consumption leading subsequently to a high production rate of ROS. Furthermore, inflammation, aging, genetic factors, and environmental pollution, are all elements promoting ROS generation and impairing antioxidant defense mechanisms and thereby representing risk factors leading to oxidative stress. An abnormal redox status was shown to be involved in the pathophysiology of various ocular diseases in the anterior and posterior segment of the eye. In this review, we aim to summarize the mechanisms of oxidative stress in ocular diseases to provide an updated understanding on the pathogenesis of common diseases affecting the ocular surface, the lens, the retina, and the optic nerve. Moreover, we discuss potential therapeutic approaches aimed at reducing oxidative stress in this context.

## 1 Introduction

### 1.1 Definition of oxidative stress

Oxidative metabolism primarily occurs in the mitochondria and endoplasmic reticulum of eukaryotic cells, where metabolic byproducts, such as free radicals, are generated during physiologic oxidation-reduction reactions [1]. Free radicals contain unpaired electrons and together with peroxides or other electrophilic molecules are known as reactive oxygen species (ROS) and reactive nitrogen species (RNS) [1,2]. They are produced as byproducts of normal cellular metabolism, and an imbalance between the generation of ROS/RNS and the antioxidant defense system is termed oxidative or nitro-oxidative stress [1,3]. ROS are extremely reactive molecules, which can modify and damage cellular macromolecules, including DNA, proteins, and lipids. ROS accumulation can lead to oxidative damage, which is involved in the development of various diseases, including cancer, diabetes, cardiovascular diseases, neurodegenerative disorders, and several ocular diseases [3].

### 1.2 Sources and types of reactive oxygen species

Reactive species can be classified based on their chemical structure. The most relevant are ROS and RNS. Additionally, there are several other subgroups of species like reactive chlorine species (RClS), reactive sulfur species (RSS) and reactive bromine species (RBrS) [4]. Moreover, there are radical reactive species, such as superoxide (O_2_^•−^), hydroxyl (^•^OH), hydroperoxyl (HOO^•^), peroxyl (ROO^•^), alkoxyl (RO^•^), and nonradical reactive species, such as hydrogen peroxide (H_2_O_2_), ozone (O_3_), singlet oxygen (^1^△g) and hypochlorous acid (HOCl) [3]. Several endogenous sources contribute to oxidative stress. Approximately 90% of ROS are generated by the mitochondrial electron-transport chain, but they can also be produced by electron chains in the endoplasmic reticulum [2]. Cellular respiration in the mitochondria occurs in the electron transport chain, where oxygen (O_2_) is reduced to generate high energy phosphate bonds in the form of adenosine triphosphate (ATP) via oxidative phosphorylation [5]. During this process, active electrons may escape the electron transport carriers and reduce molecular oxygen superoxide anion (O_2_^•−^), which is then converted to H_2_O_2_ by enzymatic reactions. Furthermore, the reduction of H_2_O_2_ leads to the formation of OH^•^ and OH^−^. The hydroxy radical is one of the most reactive ROS [2,6,7].

Cellular enzymes represent another source of ROS [6]. One of the main enzymes responsible for ROS production is the nicotinamide adenine dinucleotide phosphate (NADPH) oxidase (NOX). NOX is a membrane-bound enzyme with several subunits, which produce superoxide from molecular oxygen using NADPH as electron donor. Different isoforms of NOX are co-expressed or predominantly expressed in various cell types, such as phagocytes (NOX2), colon epithelial cells (NOX1), vascular cells (NOX4), or lymphoid tissue cells (NOX5). Their expression is regulated primarily at the transcriptional level, but epigenetic mechanisms have also been reported [8]. During their activation, electrons are transferred to flavin adenine nucleotide (FAD), passed through heme groups, and finally passed to molecular oxygen, resulting in the generation of superoxide anions [6]. It is noteworthy that ROS are essential for several physiological functions, such as microbial killing or inflammasome activation [6].

ROS are also produced as byproducts in various metabolic processes. Enzymes like xanthine oxidase (XO) also form superoxide and H_2_O_2_ [9]. During endothelial dysfunction, uncoupled endothelial nitric oxide (NO) synthase (eNOS) generates superoxide instead of NO [9]. Moreover, cyclooxygenases (COX), phospholipase A2, lipoxygenase and cytochrome p450-catalyzed reactions also contribute to the generation of ROS [[9], [10], [11]]. Moreover, ROS formation is closely related to inflammation, as excessive amounts of free radicals are secreted by inflammatory cells, which are further stimulated by ROS [12].

In addition to endogenous sources of ROS, exogenous sources play a crucial role in their generation. Air pollution, tobacco smoke, ultraviolet (UV) radiation and traffic noise represent important exogenous sources of ROS [[13], [14], [15], [16]]. Anthropogenic air pollution and subsequent chemical exposure induce ROS production in the human respiratory tract and in the cardiovascular system [13,17]. Tobacco smoke contains a complex mixture of potentially toxic chemicals that directly generate ROS. Additionally, smoke-induced inflammation contributes to an indirect stimulation of ROS generation [18]. Exposure to UV radiation is another significant factor inducing ROS generation. It stimulates the synthesis of NO synthase and affects the enzyme, catalase, leading to increased ROS production. Furthermore, DNA and other chromophores can be modified through UV radiation, resulting in elevated ROS levels [19,20].

### 1.3 The antioxidant defense system

To counteract the destructive effects of ROS, cells are equipped with a complex antioxidant defense system. This system includes enzymes like superoxide dismutase (SOD), catalase (CAT), heme oxygenase (HO) and glutathione peroxidase (GPX), as well as non-enzymatic antioxidants like vitamin E, vitamin C, and glutathione (GSH), which act as antioxidant scavengers [6].

Gene expression of antioxidant enzymes is mainly regulated by the nuclear transcription factor nuclear factor erythroid 2-related factor 2 (Nrf2). Hence, it is not surprising that dysregulation of Nrf2 has been associated with numerous diseases related to oxidative stress [21].

Three different isoforms of SOD have been identified, which are found in the mitochondria, the cytosol and the extracellular matrix [4]. SOD belongs to the family of metalloenzymes and converts O_2_^•−^ to H_2_O_2_, which is then converted by CAT to H_2_O and O_2_ [2]. Enzymes belonging to the family of peroxidases, such as GPX, are also capable of neutralizing lipid hydroperoxides. In this process, GSH acts as a reducing agent and can also scavenge ROS directly [2]. Reduced GSH is an antioxidant tripeptide that acts as a potent redox buffer due to its ability to convert to its oxidized form. It is also capable of generating cellular antioxidants like vitamin C and E, in their active form. GSH reductase balances the level of reduced GSH by reducing GSH disulfide [2,20]. NADPH and its oxidized product NADP^+^ represent another relevant cellular redox buffer, serving as an important coenzyme for GSH reductase. Apart from its role in GSH regeneration, NADPH is also used by other antioxidant enzymes, such as thioredoxin reductase and peroxiredoxin, to scavenge ROS and protect cells from oxidative damage [[22], [23], [24]]. Moreover, it features direct antioxidant functions via reduction of different radicals [25]. Aldehyde dehydrogenase is a NAPD^+^-dependent enzyme, which is critical in the prevention of UV-induced oxidative damage in ocular structures by scavenging UV-induced free radicals. In addition, it is involved in the production of NADPH, thereby contributing to antioxidant GSH levels [20]. The reductive potential of NADPH is maintained by glucose-6-phosphate dehydrogenase in the pentose phosphate pathway [26].

There are also several non-enzymatic antioxidants involved in the antioxidant defense system. Ascorbate (vitamin C) represents a strong electron donor for free radicals, superoxide anions, hydroxyl and peroxy radicals [20]. GSH and NADPH contribute to the regeneration of oxidized vitamin C. Notably, elevated levels of ascorbate were found in diurnal animals compared to nocturnal species, indicative of a protective role against UV radiation [27]. α-Tocopherol (vitamin E) is another fat-soluble chain-breaking antioxidant that can interrupt lipid peroxidation by intercepting peroxyl radicals [28]. Moreover, a synergism between ascorbate and α-tocopherol has been described [28]. α-Tocopherol is also involved in the regeneration of other antioxidants [20]. In addition, uric acid, the final product of purine metabolism in humans, is a water-soluble molecule acting as potent scavenger of singlet oxygen and hydroxyl radicals and is involved in regulation of the redox state of the GSH-ascorbate system [20,29]. Retinol (vitamin A) is an essential agent involved in visual function and antioxidant actions by capturing ROS, neutralizing radicals and stabilizing peroxyl radicals [30]. There are several additional small molecules, such as l-cysteine, l-tyrosine, albumin or ferritin, which prevent Fenton reaction-derived ROS and possess antioxidant potential [20].

### 1.4 Molecular mechanisms of oxidative damage

ROS generation is a physiological process in healthy individuals, which is balanced by the antioxidant defense system before oxidative damage occurs. However, when the production of ROS exceeds the capacity of the antioxidant defense system, oxidative stress emerges, which contributes to the pathogenesis of numerous diseases [3]. ROS can damage cellular macromolecules by modifying proteins, lipids, and DNA. ROS will react with DNA molecules, leading to the oxidation of bases and deoxyribose [5]. This may cause base modification, rearrangement of DNA sequences, miscoding of DNA, gene duplications, or activation of oncogenes [6]. These mechanisms are assumed to be involved in carcinogenesis [6]. ROS can also damage mitochondrial DNA (mtDNA), which encodes proteins of the mitochondrial respiration chain. Defective mtDNA may lead to mitochondrial dysfunction and further aggravate ROS formation [31]. Moreover, ROS can induce protein oxidation. Surface-exposed methionine and cysteine residues are especially susceptible to oxidation, which may alter protein function [7]. Furthermore, cross-linking, protein misfolding, fragmentation and aggregation may occur [5]. Cell membranes are largely composed of lipids, as well as cell organelles. Membrane polylipids contain polyunsaturated fatty acids particularly sensitive to oxidative stress. As lipid peroxidation occurs, endogenous aldehydes and their derivates, such as glyoxal, methylglyoxal (MG), malondialdehyde (MDA) or 4-hydroxy-2-nonenal (4-HNE), are formed with high reactivity and toxicity for cell components. Additionally, the formation of adducts with cellular proteins and DNA, as well as increased inflammation, is possible [5,32,33]. Moreover, cytokines and several transcription factors like nuclear factor kappa B (NF-κB) and hypoxia-inducible factor-1α (HIF1-α) are regulated by ROS [34]. A result of these events is the induction of autophagy and apoptosis, driving to cell death and further oxidative damage [35,36]. Such events can aggravate inflammation and cellular stress responses, further increasing ROS levels and promoting the vicious circle of oxidative stress.

### 1.5 Therapeutic approaches to oxidative stress

Therapeutic strategies to decrease oxidative stress include administration of antioxidants to scavenge ROS and the modulation of cellular signaling pathways that regulate ROS production and antioxidant defense mechanisms. The therapeutic effects of antioxidants, e.g., SOD and mimetics, peroxidase and mimetics, vitamin E, C or A have been studied in various oxidative stress-stimulated diseases. The therapeutic effects of antioxidants are, however, controversial, and there is some evidence that high doses of antioxidants may induce side effects and increase the risk of total mortality [6,37]. Inhibitors of ROS generation can antagonize specific ROS-generating systems. NOX inhibitors, allopurinol or xanthine oxidase inhibitors may reduce ROS levels [6]. Another approach is modification of cellular signaling pathways that regulate ROS production and antioxidant defense. For example, activation of Nrf2 can trigger antioxidant enzyme expression and diminish oxidative stress [38].

### 1.6 Oxidative stress and ocular diseases

Because of its permanent exposure to light and its composition of several susceptible tissues with high metabolic activities, the visual system is very vulnerable to oxidative stress. Particularly, the anterior part of the eye, including the conjunctiva, cornea and lens, is highly exposed to UV radiation [12]. To meet these challenges, these structures are equipped with a complex and regulated antioxidant system [39]. When ROS formation exceeds the capacity of the antioxidant defense systems, oxidative stress occurs, which is involved in the pathophysiology of various anterior ocular segment disorders, such as dry eye disease (DED) or pterygium. Additionally, cataract and a variety of corneal diseases, including keratoconus, Fuchs endothelial corneal dystrophy, diabetic keratopathy are triggered by oxidative stress [36,40]. The posterior ocular segment, including the optic nerve and retina, exhibits high metabolic rates and high oxygen consumption compared to other body tissues. Hence, larger amounts of ROS are generated in the mitochondria of these neuronal structures. Because of low antioxidant enzyme levels and an abundant content of oxidizable structures, these structures are prone to oxidative damage [4]. Vascular dysfunction is recognized to be part of the pathogenesis of several retinal pathologies, such as diabetic retinopathy (DR), age-related macular degeneration (AMD), retinal vessel occlusion, retinopathy of prematurity and glaucoma [41]. Modification of cellular signaling pathways by ROS is intimately involved in vascular dysfunction and is thereby essentially contributing to the pathogenesis of ocular diseases associated with abnormal ocular perfusion [42]. In this review, we summarize sources of ROS, oxidative stress-activated signaling pathways and antioxidant mechanisms in ocular structures. Moreover, we display the importance of oxidative stress in the pathogenesis of frequent ocular diseases, except for Leber’s hereditary optic neuropathy (LHON), and discuss potential therapeutic options. We excluded, however, ocular injuries and infections as well as complex multifactorial inflammatory diseases, such as uveitis, since this would open more large chapters and go beyond the scope of this review.

## 2 Oxidative stress in ocular diseases

### 2.1 The ocular surface

The ocular surface comprises the surface and epithelia of the conjunctiva, cornea, main and accessory lacrimal glands, and meibomian glands, as well as their apical and basal matrices, the eyelashes with their associated glands, the eyelid components responsible for the blink, and the draining tear ducts. The conjunctiva, a transparent mucous membrane, lines the inner surface of the eyelids and covers the anterior surface of the eye globe, except the cornea. It consists of two parts: the palpebral conjunctiva, which covers the inner eyelids, and the bulbar conjunctiva, covering the eyeball [43]. The bulbar part is loosely adherent to the underlying Tenon’s capsule [43]. Comprising non-keratinized, stratified squamous epithelium, a layer of loose connective tissue, and a highly vascularized layer of substantia propria, the conjunctiva plays a critical role in maintaining ocular immune defense and preventing infection, through its numerous blood vessels, lymphatic vessels, and immune cells [44]. Goblet cells, secreting mucin, are abundant in this organ, especially in the upper eyelid. The conjunctiva also contains lymphoid follicles, which contribute to the immune defense of the ocular surface [45]. The main role of the conjunctiva is to safeguard the ocular surface from extrinsic irritants and pathogens, to maintain moisture, and to provide a smooth surface for the eyelids to move over. The conjunctiva is also involved in tear film production and tear drainage.

**Fig. 1:**
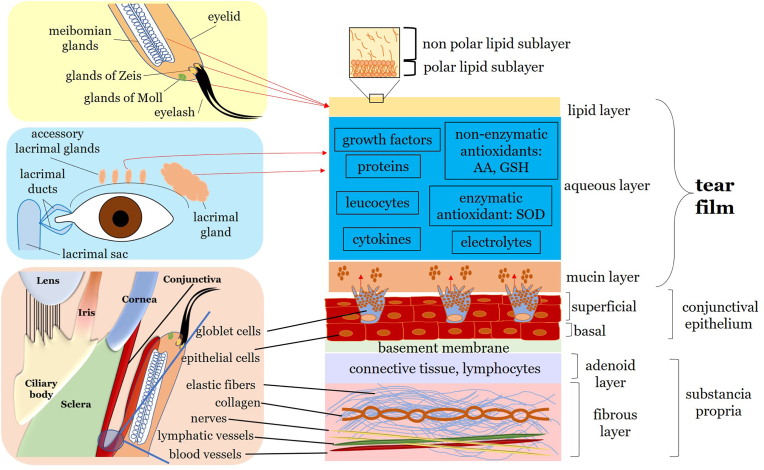
Structure of the ocular tear film and conjunctiva. The ocular tear film is composed of three different layers. The lipid layer is produced by the Meibomian glands located in the eyelids and is separated into a non-polar lipid sublayer and a polar lipid sublayer. The aqueous layer is produced by the lacrimal gland and accessory lacrimal glands and contains various proteins and electrolytes, including growth factors, cytokines, and the antioxidant defense system of the ocular tear film. The mucin layer is the innermost layer secreted by conjunctival goblet cells. The ocular conjunctiva consists of the conjunctival epithelium, a non-keratinized stratified squamous epithelium, and the substantia propria with vessels, connective tissue and lymphocytes. AA: ascorbic acid; GSH: glutathione; SOD: superoxide dismutase.

The ocular tear film nutrifies, lubricates and protects the ocular surface and is composed of three main layers [46]. The outermost lipid layer, provided by Meibomian glands, reduces evaporation and protects the ocular surface from external contaminants [47]. The middle layer, or aqueous layer, primarily supplied by the main and accessory lacrimal glands, contains water, electrolytes, proteins, antimicrobial agents, cytokines, vitamins, immunoglobulins, peptide growth factors, and hormones, providing oxygen, nutrients, and other essential compounds to the ocular surface [46,48]. The innermost mucin layer, provided by conjunctival goblet cells, helps to spread the tear film evenly over the surface of the eye and to anchor it to epithelial cells. Tear film stability is regulated by a complex balance of tear secretion, evaporation, drainage, absorption and osmolality [46]. The composition of the tear film and the structure of the conjunctiva are presented in Fig. 1.

As the first layer of defense against external potential oxidative stimuli, the ocular tear film harbors an antioxidant defense system. Several antioxidant factors, including ascorbic acid, cysteine, GSH, tyrosine and uric acid, can be found in the ocular tear film and may protect from oxidative stress under physiologic conditions [12,49]. In addition to these nonenzymatic antioxidants, SOD isoenzymes have been found in the ocular tear film [39,50].

#### 2.1.1 Dry eye disease

##### 2.1.1.1 Clinical insights into dry eye disease

**Fig. 2:**
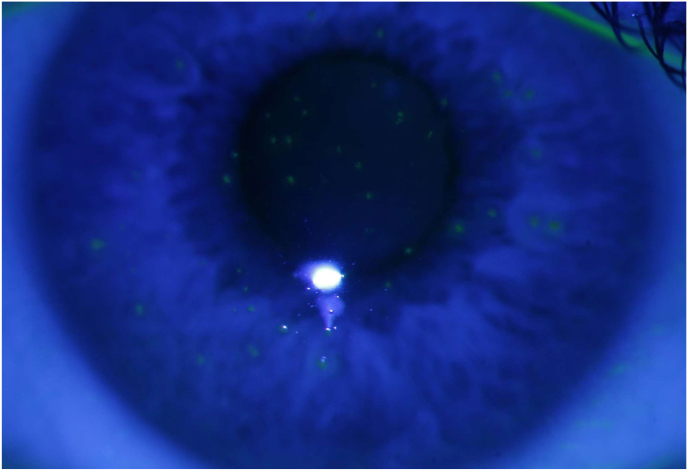
Superficial punctate keratitis in a patient with dry eye disease. This slit lamp photograph of the anterior eye segment stained with sodium fluorescein-containing drops reveals fluorescein-positive yellow spots on the corneal surface, which represent epithelial defects often seen in patients with dry eye disease. Unpublished image. Department of Ophthalmology, University Medical Center Mainz. (For interpretation of the references to colour in this figure legend, the reader is referred to the Web version of this article.)

Dry eye disease (DED) is a multifactorial disorder characterized by a deficiency in tear production or excessive tear evaporation resulting in ocular surface disruption, inflammation and discomfort. It is a common condition affecting people of all age categories, but with elevated prevalence in older individuals and women [51,52]. The pathogenesis of DED involves a complex interplay of various factors, including inflammation of the ocular surface, instability of the tear film, and neurosensory abnormalities. Common risk factors for DED include aging, hormonal changes, autoimmune diseases, environmental factors, and certain medications [53]. Symptoms of DED can vary widely, but common complaints include dryness, burning, itching, tearing, and a foreign body sensation. Diagnosis of DED involves a comprehensive ocular surface evaluation, including assessment of tear film quality, quantity, and stability, as well as measurement of tear osmolarity and ocular surface staining. Additional tests, such as meibomian gland imaging and assessment of blink rate and completeness, may be necessary in specific cases. Subjective scales, such as Ocular Surface Index (OSDI), play also a crucial role in diagnosing DED [51]. The treatment of DED typically involves a combination of pharmacological and non-pharmacological interventions, depending on the severity and underlying causes of the condition. A representative picture of DED is demonstrated in Fig. 2.

##### 2.1.1.2 Mechanisms of oxidative stress in dry eye disease

Oxidative stress essentially contributes to the development and progression of DED. Based on a meta-analysis, various oxidative stress markers, including lipid peroxide, myeloperoxidase, NO synthase, xanthine oxidase/oxidoreductase, 4-HNE, and MDA were found to be elevated in tear fluid and conjunctival specimens from patients with DED [54]. Moreover, impaired antioxidant functions have been detected in DED patients. For example, expression of CAT, GPX, and SOD was reduced in conjunctival epithelial cells from patients with DED compared to healthy controls [55].

Several environmental factors have been shown to induce oxidative stress on the ocular surface. For example, air pollution has been reported to trigger ROS production in the conjunctiva and cornea, contributing to DED [56]. Moreover, gaseous substances, such as O_3_ and NO_2_ may irritate the ocular surface and exacerbate oxidative stress [57]. One of the most relevant environmental sources of oxidative stress is exposure to UV radiation. UVA, UVB, and UVC radiation trigger DNA damage and promote the production of H_2_O_2_, the most frequently found ROS from UV radiation, damaging cellular proteins, lipids, and DNA [20,58]. The main neutralizing enzyme of H_2_O_2_ is SOD, which was reported to be diminished in the ocular tear fluid from patients with DED [55,59].

Aging is another well-established risk factor for DED, associated with a decline in antioxidant defense mechanisms and an increase in oxidative stress [60]. The rate of O_2_^•−^ and H_2_O_2_ production increases, inducing a pro-oxidative shift and accumulation of oxidatively damaged molecules [61]. Nrf2 signaling, an important regulator of antioxidant defense, is impaired during aging [60], leading to reduced levels of antioxidant enzymes like CAT or SOD [62]. Mice lacking SOD exhibit a phenotype with accelerated aging, showing alterations in the lacrimal gland, reduced tear production, and increased inflammation [63]. Accumulation of lipofuscin granules in the lacrimal gland also contributes to oxidative stress [64]. Metabolic changes during aging, such as impaired insulin signaling and the consequent aggregation of advanced glycation end products (AGEs) are associated with oxidative stress and inflammation in the lacrimal gland [64].

Inflammation of the ocular surface, a condition highly interconnected with oxidative stress, is typically present in DED [12]. ROS induce the expression of inflammatory cytokines such as interleukin-1 (IL-1), interleukin-6 (IL-6) and tumor necrosis factor α (TNF-α) activating multiple inflammatory pathways [65]. Conversely, immune cells also release significant amounts of ROS, such as O_2_^•−^, H_2_O_2_, further exacerbating the vicious circle between oxidative stress and inflammation [66]. Elevated levels of cytokines and chemokines have been found in the tear fluid of patients with symptomatic DED [67]. There is also a correlation between IL-6 levels, disease severity and ocular surface parameters [68]. Stimulation of conjunctival epithelial cells with TNF-α and interferon- γ (INF-γ) leads to an increased secretion of IP-10/CXCL10, a potent chemoattractant for immune cells, further aggravating oxidative stress and inflammation [69,70]. In mice with reduced tear volume, mononuclear infiltration and fibrosis of the lacrimal gland were observed [71].

The tear film has a crucial function in protecting the ocular surface from external insults and providing a smooth optical surface. In DED, the tear film becomes unstable and hyperosmolar. Hyperosmolarity of the tear film is a critical step in the pathogenesis of DED. It initiates morphological changes, such as apoptosis of conjunctival and corneal cells, and activates inflammatory pathways, resulting in the loss of mucin-producing goblet cells, further exacerbating tear film instability [72]. Hyperosmolar stress stimulates generation of inflammatory cytokines, e.g., IL-1β and TNF-α, promoting ocular surface inflammation [73]. Primary human corneal epithelial cells exposed to hyperosmolar stress displayed elevated oxidative stress markers and a disrupted balance of oxygenases and antioxidant enzymes [74]. Hyperosmolarity also resulted in mitochondrial DNA damage. In this regard, mitochondria-induced oxidative damage was associated with lacrimal gland inflammation and dysfunction in transgenic mice [71].

Tear film stability may also be affected by dysfunctional meibomian glands, which produce the lipid compound of the tear film. Meibomian gland dysfunction can result in a reduced quantity and quality of the lipid layer, resulting in increased tear evaporation and consequent oxidative stress [75]. Mice lacking SOD1 exhibited meibomian gland dysfunction with elevated levels of the oxidative stress markers, 4-HNE and 8-hydroxy-2′-deoxyguanosine (8-OHdG), as well as inflammatory cell infiltrates in the acinar epithelium of meibomian glands [76]. Notably, lipids are highly prone to oxidative degradation, further reducing tear film stability under oxidative stress conditions [75]. To counteract lipid peroxidation, meibomian glands also possess antioxidant enzymes, such as GPX [77]. However, these antioxidant functions may be hampered under pathological conditions [12]. Dysfunction of the lacrimal gland also contributes to the pathophysiology of DED. In a transgenic mouse model encoding the *mev-1* gene, which induces oxidative stress, mitochondrial oxidative damage and inflammation of the lacrimal gland was detected. This resulted in reduced protein and aqueous secretory functions, leading to increased ocular surface epithelial damage [78]. In a study of our own, aged mice lacking the M_3_ muscarinic acetylcholine receptor developed tear fluid deficiency with signs of DED and a pronounced increase of oxidative stress markers and prooxidative redox and inflammatory genes in the corneal and conjunctival epithelium [79]. Additionally, in aging rats, Rab GTPases were reported to be involved in exocytosis of tear components, and antioxidant markers were found to be decreased [80]. Apart from direct disturbances in the lacrimal gland, oxidative stress may impair innervation. The ocular surface is innervated by sensory nerve endings of the trigeminal nerve, and acini of the lacrimal gland are innervated by efferent sympathetic and parasympathetic nerves. A pro-oxidant state may damage the nerve’s myelin, driving to impaired innervation of the lacrimal gland, which may result in reduced tear secretion [64,81].

**Fig. 3:**
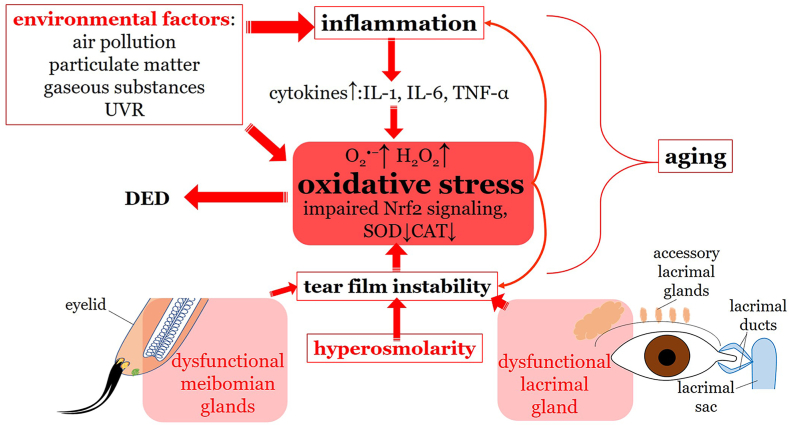
Role of oxidative stress in the pathogenesis of dry eye disease. Environmental factors, such as air pollution, particulate matter, gaseous substances or UVR as well as aging lead to increased inflammation with consequent imbalance between ROS generation and the antioxidant defense. Additionally, hyperosmolarity and instability of the tear film further aggravates inflammation and oxidative stress contributing to the vicious circle of dry eye disease. DED: dry eye disease; UVR: ultraviolet radiation; IL-1: interleukin 1; IL-6: interleukin 6; TNF-α: tumor necrosis factor α; O_2_^•−^: superoxide; H_2_O_2:_ hydroxyl peroxide; Nrf2: nuclear factor erythroid 2-related factor 2; SOD: superoxide dismutase; CAT: catalase.

In summary, oxidative stress in DED can arise from various sources, including tear film instability, inflammation, lacrimal and meibomian gland dysfunction, environmental factors, and aging. Knowing the oxidative stress sources in DED is crucial for developing targeted therapies to prevent or limit the damage caused by ROS in this condition. A scheme on the role of oxidative stress in DED is presented in Fig. 3.

##### 2.1.1.3 Therapeutic approaches for oxidative stress in dry eye disease

The therapeutic approaches for oxidative stress in DED aim to limit the production of ROS and to enhance the antioxidant defense mechanisms of the ocular surface. These targets include both pharmacological and non-pharmacological approaches, which can be used alone or in combination to manage the symptoms of DED.

Antioxidants, such as vitamin A and E, coenzyme Q10 or lipoic acid, can be administered topically in tear substitutes to scavenge ROS and to prevent from their harmful effects to the ocular surface [82]. Other studies evaluated the therapeutic potential of lactoferrin in DED. Lactoferrin is an iron-binding glycoprotein, which plays a pivotal role in enhancing iron retention while also exerting antioxidant functions by limiting the production of free radicals and mitigating inflammation. It is an important component of the ocular tear film and is usually produced by corneal epithelial cells and the lacrimal gland [83]. Decreased levels of lactoferrin in the tear fluid were found in patients with DED and were found to be negatively correlated with DED symptoms [84]. Moreover, studies in an animal model of DED revealed that uptake of selenium-binding lactoferrin by a receptor into corneal epithelial cells could prevent corneal damage [85]. In animal models of corneal epithelial damage and DED, topical administration of lactoferrin reduced inflammation and promoted wound healing [86,87]. In patients with Sjögren’s syndrome and cataract surgery-induced DED, oral administration of lactoferrin, ameliorated tear film stability and reduced DED symptoms [88,89]. In addition, lactoferrin-loaded contact lenses were proposed to protect the corneal epithelium from oxidative stress in ocular surface pathologies [90].

After cataract surgery, DED is a common problem, and postoperative application of eye drops containing preservatives, such as benzalkonium chloride, known to trigger ocular surface oxidative stress and to cause Meibomian gland dysfunction and DED [91,92]. Comparison between postoperatively administered preservative-free and preserved eye drops showed decreased parameters of DED, reduced inflammation, and higher levels of antioxidants in the group treated with preservative-free eye drops [93,94]. These findings highlight the possibility to minimize oxidative stress with consequent DED by using preservative-free eye drops after cataract surgery.

Iodide is an oxygen free radical scavenger acting as a reducing agent and electron donor [95]. Iodide iontophoresis represents a therapeutic technique that involves the application of an electronic current to facilitate the movement of iodide ions through body surfaces. This technique has been used to enhance the water-soluble antioxidant capacity of the ocular tear fluid [75,96]. Patients with DED treated with iodide iontophoresis showed improved subjective symptoms, better tear film stability and reduced morphologic changes [95]. However, a limiting factor for the use of iodine are its potential cytotoxic effects to corneal epithelial and endothelial cells [97].

There are also several studies using nanoparticles to develop therapies to face DED. For example, xanthohumol induced the production of antioxidant transcription factors, such as Nrf2, regulating antioxidant enzymes. Moreover, xanthohumol-encapsulating poly (lactic-co-glycolic acid) nanoparticles showed cytoprotective effects against oxidative stress in vitro and in a mouse model in vivo [98]. In summary, there are several topical therapeutic approaches aimed at targeting oxidative stress in DED include the reduction of inflammation and the enhancement of antioxidative defense mechanisms. For more studies concerning antioxidants in the treatment of DED, please refer to Table 1 in the online supplement.

#### 2.1.2 Pterygium

##### 2.1.2.1 Clinical insights into pterygium

**Fig. 4:**
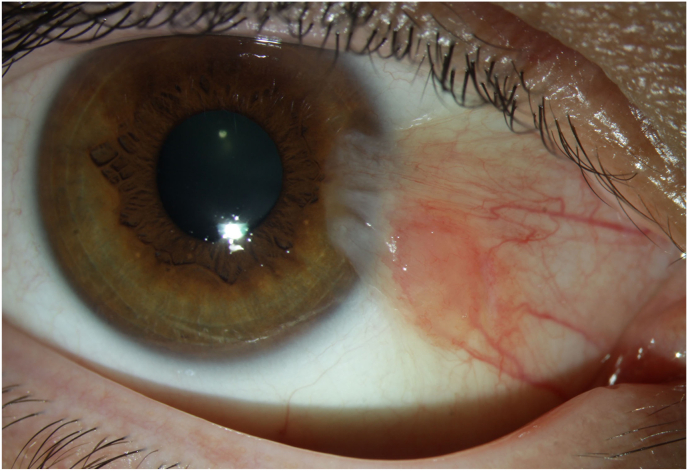
Pterygium in a 30-year-old man. The photograph of the anterior segment of the right eye shows the typical appearance of a pterygium with a fleshy, triangular-shaped mass of conjunctival tissue growing from the nasal side onto the cornea. Unpublished image. Department of Ophthalmology, University Medical Center Mainz.

Pterygium is a widespread ocular surface disease characterized by a fibrovascular conjunctival degeneration with the subsequent growth of a fleshy, triangular-shaped mass of conjunctival tissue onto the cornea [99]. The pathogenesis of pterygium involves genetic, environmental, and immunological factors. Chronic exposure to UV radiation, dry and dusty environments, genetic susceptibility, increasing age and male gender have been identified as major risk factors for pterygium [99,100]. Most pterygia are located nasally in the interpalpebral zone [99]. A photograph of pterygium is shown in Fig. 4.

Patients with pterygium may suffer from visual loss, conjunctival redness, irritation, dryness and epiphora [99]. Diagnostic evaluation of pterygium usually involves visual acuity testing, slit-lamp examination, and measurement of its size. Treatment is usually initiated in cases of visual impairment, rapid growth, or discomfort. Conservative options include lubricants and sunglasses. The standard surgical therapy is excision with a conjunctival or limbal-conjunctival autograft [99].

##### 2.1.2.2 Mechanisms of oxidative stress in pterygium

Oxidative stress contributes to the pathogenesis of pterygium. In samples of patients with pterygium, a higher serum total oxidant status and higher compensatory total antioxidant status were detected compared to a healthy control group [101]. In pterygium specimens, enhanced labeling of proteins modified by 4-hydroxyhexenal and 4-hydroxynonenal, which are reactive aldehydes derived from nonenzymatic oxidation of n-3 and n-6 polyunsaturated fatty acids, has been found, suggesting a potential role of oxidative stress in its pathogenesis [102].

The activity of antioxidant enzymes, such as CAT, SOD and GPX, was significantly reduced in pterygium samples compared to conjunctiva from healthy controls. Increased levels of NO and MDA, a marker for lipid peroxidation, have also been found in patients with pterygium [103]. Increased NO levels may be a consequence of elevated inflammation with consequently higher levels of endotoxins and cytokines. For example, positive staining for COX2 was detected in most pterygium specimens [104]. Notably, GSH S-transferase was elevated in pterygium specimens, which may be a compensatory response to free radical formation [105].

UV radiation is discussed to be the main risk factor in the pathogenesis of pterygium. UVB light is supposed to be the most biologically active form of UV radiation, thereby playing a crucial role in the pathogenesis of pterygium [106]. Its phototoxic effects induce oxidative stress and damage of cellular DNA [106]. Immunohistochemical studies revealed significantly higher levels of the oxidative DNA damage marker, 8-OHdG, in pterygium specimens compared to normal conjunctival tissue [107].

Oxidative stress is associated with increased expression of ALDH3A1, PDIA3 and PRDX2. These enzymes may provide a certain resistance to UV-induced apoptosis, and a hyperproliferative stage may be promoted [108]. These results indicate that the pathogenesis of pterygium may be a consequence of hampered apoptosis and uncontrolled cell proliferation [109].

UV radiation and consequent ROS excess can activate the extracellular-signal regulated kinase (ERK), a mitogen-activated protein kinase (MAPK) pathway [110]. By activating this pathway, different growth factors, such as matrix metalloproteinase-1 (MMP-1), interleukins and VEGF, which play a crucial role in the pathogenesis of pterygium, are also activated [110,111]. MMP-1 was found to be expressed by pterygium-diseased cells dissolving Bowman’s layer and thereby playing a key role in the formation and migration of pterygium [112]. Other authors also detected increased levels of MMP-1 and MMP-3 in pterygium head fibroblasts, initiating corneal invasion [113].

Elevated expression of interleukins, such as IL-6 and IL-8 was observed in patients with pterygium induced by UVB radiation [114]. Increased levels of VEGF-165, VEGF-A, the endothelial junction protein CD31 and the lymphatic marker D2-40 as well as elevated blood and lymphatic vessel counts in pterygium specimens compared to normal conjunctiva support the fact that lymphangiogenesis and angiogenesis are pivotal for the pathogenesis of pterygium [115]. UVB radiation and oxidative stress have also been shown to trigger the NF-κB pathway in keratocytes and in ocular surface epithelium [116,117]. Activation of this pathway with consecutively increased expression of NF-κB-induced inflammatory genes was detected in patients with pterygium [[118], [119], [120]].

In summary, chronic exposure to environmental stressors, particularly UV radiation, can initiate ROS production on the ocular surface. Decreased levels of antioxidant enzymes and insufficiency of apoptosis further exacerbate oxidative stress and contribute to the pathogenesis of pterygium.

##### 2.1.2.3 Therapeutic approaches targeting oxidative stress in pterygium

Surgical removal of pterygium with conjunctival autografting is the standard therapy to treat symptomatic pterygium [99]. Recurrence of pterygium after surgical excision is a major problem. In this regard, elevated indicators for oxidative stress and lower levels of antioxidant enzymes were found in recurrent pterygia compared to primary pterygia [121]. Thereby adjuvant postoperative therapies should face this problem.

Administration of antioxidants may be a therapeutic tool in the treatment of pterygium. Phenolic compounds, such as flavonoids, carotenoids, curcumin, ellagic acid and chalcones, are supposed to exert anti-inflammatory effects and to reduce oxidative stress [122]. For example, curcumin exerts antioxidant, anti-angiogenic and anti-inflammatory properties, and these effects were also demonstrated in human pterygium fibroblasts with consequent inhibition of proliferation [123].

Other therapeutic approaches target ROS-dependent signaling molecules, such as proangiogenic and proinflammatory factors. For example, some authors reported that perioperative subconjunctival injection of bevacizumab, a recombinant and humanized anti-VEGF antibody, reduced the recurrence rate of pterygium [[124], [125], [126]]. Experiments in cultured pterygium fibroblasts revealed suppressed cell migration and reduced expression levels of MMP-3 and MMP-13 after treatment with the immunosuppressive agent, cyclosporine A [127]. In addition, adjuvant use of cyclosporine A with pterygium excision was reported to reduced recurrence rates [128,129]. Notably, antioxidant eye drops that are used in DED might represent another therapeutic option to prevent pterygium onset and progression.

### 2.2 The cornea

The cornea is a transparent and avascular tissue covering the anterior segment of the eye and providing approximatively 40–44 D of the total ocular refractive power [43]. It is composed of different layers that are fundamental to protect the eye and to maintain transparency. The outermost layer is the corneal epithelium, a nonkeratinized stratified squamous epithelium composed of several cell layers providing a smooth optical surface and representing a barrier to chemicals, water and microorganisms [43]. The Bowman’s layer is composed of collagen and proteoglycans and is an acellular layer anterior to the stroma that is important for maintaining the corneal shape [43]. In humans, approximatively 80–85% of the corneal tissue is formed by the stroma, a transparent layer with a peculiar organization of stromal fibers and extracellular matrix, characterized by glycosaminoglycans and by an arrangement of collagen fibers in parallel bundles. The predominant cell type in the stroma are keratocytes, which produce the extracellular matrix. The Descemet’s membrane is located between the stroma and the corneal endothelium contributing to the overall structural integrity of the cornea and consisting of collagen and laminin. The endothelium is the most posterior layer of the cornea with contact to the aqueous humor. It is a monolayer of non-regenerative cells with decreasing cell density throughout life. These cells act as endothelial pumps to regulate corneal water content. To generate a flux of ions out of the stroma to the aqueous humor they are richly equipped with Na^+^/K^+^ ATPase and an intracellular carbonic anhydrase pathway [43]. Due to its localization at the front of the eye, the cornea is permanently exposed to high concentrations of oxygen [36] as well as to solar UV radiation [130,131]. Thereby, ROS accumulation occurs chronically. To face this problem, the cornea possesses an antioxidant defense system including nonenzymatic antioxidants, e.g., vitamin A and E, GSH and ferritin as well as enzymatic antioxidants, such as CAT, SOD and GPX [20,[131], [132], [133]]. Under specific conditions, the prooxidant and antioxidant balance may, however, become disturbed.

#### 2.2.1 Keratoconus

##### 2.2.1.1 Clinical aspects of keratoconus

Keratoconus is a generally bilateral corneal disorder characterized by their thinning and bulging. This may result in a progressive central thinning of the corneal stroma with consecutive irregular astigmatism and myopia, inducing to vision loss [134]. Histopathological studies revealed that the disease is associated with defects in the Bowman’s layer and iron deposits in the basal epithelium (Fleischer’s Ring). The progressive corneal thinning may lead to rupture of the Descemet’s membrane and acute corneal edema or even corneal perforation. The prevalence of keratoconus shows a large variation over different regions of the world [135,136]. A meta-analysis by Hashemi et al., which included more than 50 million people in 15 countries determined a global prevalence of keratoconus of 138 per 100,000 [137]. In early stages of keratoconus with mild or moderate vision impairment, glasses or rigid gas permeable contact lenses may to correct astigmatism [138]. In cases of disease progression, corneal cross-linking, intracorneal ring segments or, in advanced stages, deep anterior lamellar or penetrating keratoplasty represent established therapeutic approaches [139].

##### 2.2.1.2 Mechanisms of oxidative stress in keratoconus

While keratoconus is classically regarded a non-inflammatory disease, recent reports suggest an inflammatory component in its pathogenesis. A meta-analysis by Navel et al. showed a significant decrease of antioxidants in tear fluid, aqueous humor and blood serum of patients with keratoconus compared with healthy controls. The total antioxidant status, aldehyde/NADPH dehydrogenase, lactoferrin/transferrin/albumin, selenium/zinc were significantly decreased in keratoconus patients compared to healthy individuals [140].

Moreover, was shown that patients with keratoconus had elevated systemic markers for oxidative stress [141]. These findings indicate a possible systemic component in the development of keratoconus.

Liu et al. observed a significantly higher expression of prooxidative genes, including NOX2 and NOX4, in a rabbit cornea model for keratoconus [142]. Moreover, disturbed transcription and activity of different antioxidant enzymes was detected in keratoconus corneas [143].

**Fig. 5:**
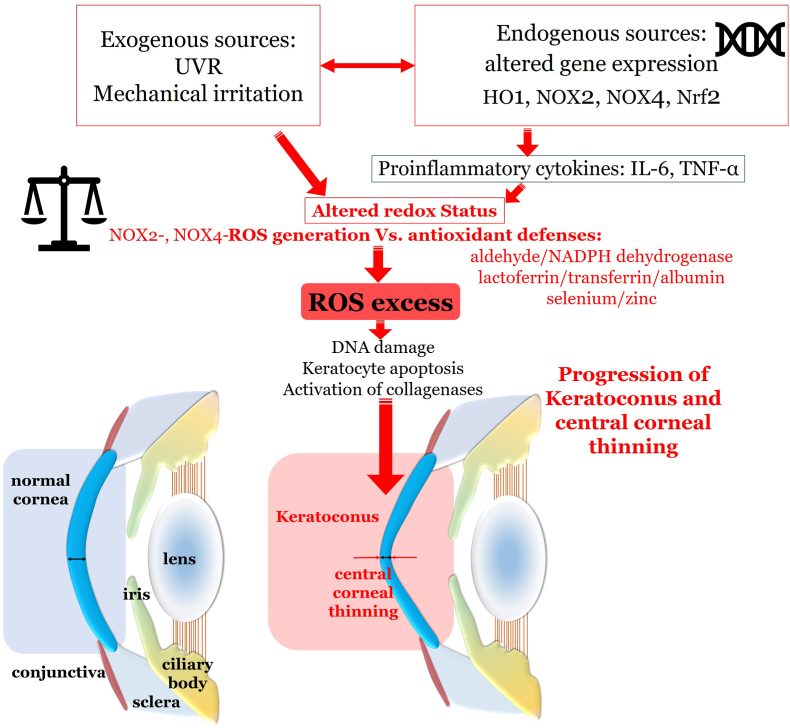
Sources and role of oxidative stress in keratoconus. Exogenous sources of oxidative stress include UV radiation and mechanical irritation. Moreover, altered gene expression of ROS-related genes, such as HO1, NOX2, NOX4 and NRF2, with consequently elevated expression of proinflammatory cytokines, such as IL-6, TNF-α and MMP, are found. By increased levels of ROS, protein damage and keratocyte death as well as changes in the extracellular matrix occur causing progressive thinning of the central cornea. UVR: ultraviolet radiation; HO1: heme oxygenase-1; NOX: nicotinamide adenine dinucleotide phosphate oxidase; Nrf2: nuclear factor erythroid 2-related factor 2; IL-6: interleukin 6; TNF-α: tumor necrosis factor α; ROS: reactive oxygen species; NADPH: reduced form of nicotinamide adenine dinucleotide.

Additionally, in the tear film of keratoconus patients an increased level of proinflammatory cytokines, such as IL-6, TNF-α and MMP was detected [[144], [145], [146]]. Given the young age at manifestation, ROS-mediated damage to mitochondrial function is currently assumed [147]. In this regard, Karamichos et al. demonstrated increased levels of lactate in fibroblasts of patients with keratoconus [148]. Due to increased H_2_O_2_ formation and DNA damage with decreased antioxidant defense at the same time, keratocyte apoptosis as well as changes in the corneal extracellular matrix may occur and lead to increased corneal thinning and disease progression [147,149]. Activation of collagenases and gelatinases by excessive ROS generation may further aggravate corneal thinning [150]. Potential ROS sources and the role of oxidative stress in keratoconus are shown in Fig. 5.

##### 2.2.1.3 Therapeutic approaches targeting oxidative stress in keratoconus

There are very few therapeutic agents that aim to reduce oxidative stress in keratoconus. Flavonoids were shown to protect against UV-induced oxidative stress by obtaining potent antioxidant capacities [151]. McKay et al. showed that quercetin, a natural flavonoid, may be able to regulate metabolic activity of human keratoconus cells and reduce inflammatory pathways, thereby representing a potential therapeutic approach in this context [152,153]. Lactoferrin-loaded contact lenses may also represent a therapeutic option in keratoconus because higher resistance to cytotoxicity in tears of keratoconus patients was demonstrated [154]. Likewise, vitamin D supplementation is supposed to have beneficial effects on inflammation, oxidative stress and collagen degradation. Stabilization in the progression of keratoconus was detected after 12 months of vitamin D supplementation [155]. Improved antioxidant capacity and reduced inflammation in patients with keratoconus were also found after oral supplementation with highly concentrated docosahexaenoic acid (DHA) triglyceride [156]. Moreover, the application of sulforaphane could activate the Nrf-2/HO1 signaling pathway with protective effects in keratoconus corneas of rabbits [157].

#### 2.2.2 Fuchs endothelial corneal dystrophy

##### 2.2.2.1 Clinical insights into fuchs endothelial corneal dystrophy

The corneal endothelium is highly prone to oxidative stress as a consequence of its life-long exposition to UV radiation and its lack of proliferative cells [158]. It is a monolayer of the posterior corneal surface. The corneal endothelium is rich in Na^+^/K^+^-ATPase as its main ion transporter to generate an osmotic gradient out of the corneal stroma, which works against the inwardly leading pressure caused by stromal glycosaminoglycans [159,160]. Therefore, these cells are commonly known as the pump cells of the cornea. In case of an insufficient osmotic gradient, corneal swelling can establish, leading to disorganization of collagen fibrils due to embedded water molecules, which causes a loss of corneal transparency and visual impairment [161,162]. This pathological set is typical for the most common primary corneal endothelial dystrophy known as the Fuchs endothelial corneal dystrophy (FECD) [163,164]. This condition is globally a major reason for corneal transplantation [164]. Therapeutic options are hyperosmotic sodium chloride drops or ointments aimed at reducing corneal edema, bandage contact lenses in advanced cases with painful epithelial bullae or surgical treatment with endothelial or penetrating keratoplasty [163].

##### 2.2.2.2 Mechanisms of oxidative stress in fuchs endothelial corneal dystrophy

The high density of ion transporters in the endothelium of the cornea results in a high metabolic activity, being the second highest energy consumption site in the eye after retinal photoreceptors [165]. The corneal endothelium is therefore prone to oxidative stress. A recent review hypothesized that cellular stress and abnormal cell interactions within the extracellular matrix may be pivotal factors in the pathophysiology of FECD [163]. Another research group reported on a down-regulation of Nrf2 in FECD [166]. A variety of antioxidant molecules are dependent on regulation by this important transcription factor [167]. It has been demonstrated that most of the energy required by the ion pumps to maintain corneal endothelial function is provided by the mitochondria [168]. Due to its lack of histone protection as well as its exposure to the ROS producing electron transport chain, mtDNA is at high risk of oxidative damage. Increased levels of 8-OHdG, a marker for oxidative DNA damage, have been observed in FECD, and it has been proposed that oxidative stress primarily targets mtDNA in the disease [161]. In comparison to healthy tissue, upregulated levels of 8-OHdG can be interpreted as a sign of accelerated aging of corneal endothelial cells affected by FECD, and a consequence of oxidative stress. In another study, exposure of the corneal endothelium to menadione, a ROS inducing substance, was shown to promote rosette formation of corneal endothelium as well as mtDNA and nDNA damage with mitochondrial dysfunction. Finally, apoptosis is induced via caspase cleavage and cytochrome *c* release [169].

**Fig. 6:**
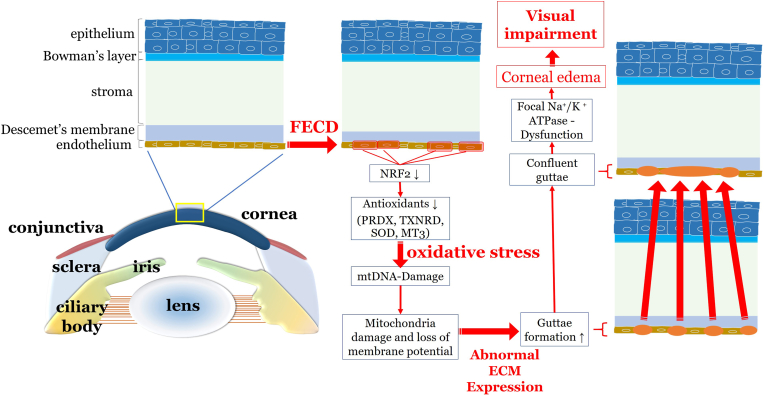
Oxidative stress related pathways in the pathogenesis of FECD. In patients with FECD, downregulation of Nrf2, which is in turn responsible for the expression of numerous antioxidants, leads to increased oxidative stress. Damaged mitochondrial DNA causes mitochondrial dysfunction with loss of membrane potential. This leads to increased formation of guttae. Mitochondrial loss further leads to dysfunction of Na^+^/K^+^-ATPase. Consecutive loss of the osmotic gradient between the anterior chamber and the corneal stroma causes water influx and stromal edema with decrease of visual acuity. Nrf2: nuclear factor erythroid 2-related factor 2; PRDX: peroxiredoxin; TXNRD: thioredoxin reductase; SOD: superoxide dismuatse; MT3: metallothionein-3; ECM: extracellular matrix; FECD: Fuchs endothelial corneal dystrophy.

Accumulation of oxidized DNA is characteristic for ROS-induced damage at the molecular level and can be detected in a variety of diseases associated with oxidative stress. It has been shown that the extent of apoptotic cell death and oxidative stress correlates with the severity of FECD [161]. A scheme of oxidative stress-related pathways in FECD is shown in Fig. 6.

##### 2.2.2.3 Therapeutic approaches targeting oxidative stress in fuchs endothelial corneal dystrophy

Limited nuclear translocation of Nrf2 and consequently a diminished activation of the antioxidant response element (ARE) was reported to play an essential role in the pathophysiology of FECD. Exposure of corneal endothelial cell lines to sulforaphane decreased ROS production, enhanced cell viability and significantly upregulated ARE-dependent antioxidants reducing oxidative stress-triggered apoptosis in FECD [170]. Lithium treatment of corneal endothelial cell cultures improved cell survival and reduced oxidative and ER stress. Increased autophagy was discussed to contribute to this effect [171]. Similar effects with elevated antioxidant markers and decreased ER stress markers were detected after treatment with N-acetlycysteine in cultured murine corneal endothelial cells with FECD [172]. Oxidative stress, inflammation and apoptosis can also be diminished by stimulation of the adenosine A_2A_ receptor. Polydeoxyribonucleotide is a registered drug activating this receptor, and an in vitro study in a FECD model with human corneal endothelial cells revealed reduced H_2_O_2_-induced damage after incubation with this agent [173]. Poly (ADP-ribose) polymerase (PARP) is associated with cell death and apoptosis in several diseases. The PARP1 inhibitor PJ34 was shown to protect the corneal endothelium from UVA-induced oxidative damage and apoptosis, representing another possible approach for the therapy of FECD [174]. The antioxidative capacity of human corneal endothelial cells was be improved after in vitro treatment with curcumin by activation of the Keap1/Nrf2/ARE pathway [175]. In advanced disease stages, corneal transplantation may be mandatory. Recently, it has been found that 2% human platelet lysate medium could represent a suitable xeno-free substitution for 2% fetal bovine serum medium in cultured human donor corneas due to beneficial alterations in gene expression, including the upregulation of HO1 with consequently reduced endothelial cell loss [176]. These findings demonstrate that there are several therapeutic approaches targeting oxidative stress in FECD. Since most of the existing agents were analyzed in vitro, further clinical studies are mandatory in this field.

#### 2.2.3 Diabetic keratopathy

##### 2.2.3.1 Clinical insights into diabetic keratopathy

Diabetes mellitus is globally a very common disease, and DR is one of the most common causes of acquired blindness in industrialized countries and will continue to increase in the approaching decades [177]. However, much less is known about other ocular complications, such as diabetic keratopathy, involving the cornea as a consequence of diabetic metabolism. In the course of peripheral diabetic neuropathy, nerve damage probably triggered by hyperglycemic conditions also affects the corneal nerve fibers, resulting in a significant reduction and change in the morphology of subepithelial nerve fibers [178]. This leads to a reduction of corneal surface sensitivity, one of the elements of diabetic keratopathy [179]. Smaller case series showed an increased risk for corneal epithelial defects and corneal wound healing disorders after intraocular surgery in diabetic patients [180,181]. In vitro experiments also demonstrated slower wound healing in diabetic corneas [182]. In diabetes, the risk of spontaneous corneal trauma was shown to be markedly increased both under laboratory and clinical conditions [177,183,184]. The management of corneal erosions can be much more challenging in a hyperglycemic state and often requires treatment options beyond conventional therapy, such as amniotic membrane coverage in combination with autologous serum eye drops [185] or insulin eye drops [186].

##### 2.2.3.2 Mechanisms of oxidative stress in diabetic keratopathy

Diabetic keratopathy is caused by changes in cytokine and growth factor expression as well as by the accumulation of advanced glycation endproducts (AGEs), which are detectable in diabetic corneas and are responsible for disturbance of epithelial cell migration, that is fundamental for the physiological corneal turnover [[187], [188], [189]]. A dedicated study reported that expression of the antioxidant receptors and enzymes was significantly reduced in mice with diabetes, whereas markers for oxidative stress were significantly increased [190]. Furthermore, decreased tear film production and increased oxidative stress were demonstrated in humans and experimental diabetic animals [191]. A complex signaling cascade related to diabetic metabolic disorders leads to the destruction of the corneal epithelium and initiates a surface disorder with superficial punctate keratopathy, persistent erosions up to corneal ulcers. AGEs play an important role in the pathophysiology of diabetic keratopathy. These are compounds formed by non-enzymatic reactions of glycans, proteins, nucleic acids and lipids. They are usually characterized by inertness, therefore tending to accumulate. AGEs can activate PKC through binding their specific receptor, namely receptor for advanced glycation end products (RAGE), which activates NOX and consequently ROS production. In case of depletion of antioxidant capacities, a ROS excess triggers the activation of the transcription factor NF-κB and the NLRP3 inflammasome. Both mechanisms can lead to a strong inflammatory reaction and cell death. Activated NLRP3 elicits production of inflammatory cytokines, such as IL-1β and IL-18. Concomitantly, activation of caspase-1 results in proteolytic division of gasdermin D, which in turn induces pyroptosis. In this context, the cell releases proinflammatory cell contents or cytokines, such as IL-1β and IL-18, via lytic pores in its membrane, thereby further escalating inflammation [192].

##### 2.2.3.3 Therapeutic approaches for oxidative stress in diabetic keratopathy

Improvement of glycemic control represents the first step in the treatment of diabetic keratopathy with consequent increase in corneal nerve fiber density [193]. Moreover, application of artificial tears may enhance epithelial barrier function, thereby reducing inflammation and ROS generation. Additionally, topical steroids and non-steroidal anti-inflammatory agents may decrease oxidative stress and ROS formation by inhibiting various pro-inflammatory mediators [194]. Thymosin β4, a naturally occurring polypeptide, has been shown to reduce corneal inflammation and improve corneal reepithelialization in ocular surface diseases [195,196]. Through inhibition of the NLRP3 inflammasome, topical calcitriol administration improved corneal healing and reinnervation in diabetic corneas [197]. Similar effects with reduced ROS accumulation were found after topical treatment with N-acetylcysteine in a diabetic mouse model [198]. Other potential therapeutic agents that lead to reduced inflammation and improved antioxidant capacities with subsequent amelioration of the diabetic corneal epithelium are β-carotene [199], pycnogenol [200] or α-lipoic acid [201].

### 2.3 The lens

The crystalline lens is a transparent biconvex structure with refractive properties. Histologically, the lens can be divided into two distinct cell subtypes: the anterior single-layered collection of cuboidal lens epithelial cells and otherwise main lens cells with elongated collagen fibers. These two types of lens cells are covered by a circular basement membrane, also termed lens capsule [202].

#### 2.3.1 Cataract

##### 2.3.1.1 Clinical presentation of cataract

**Fig. 7:**
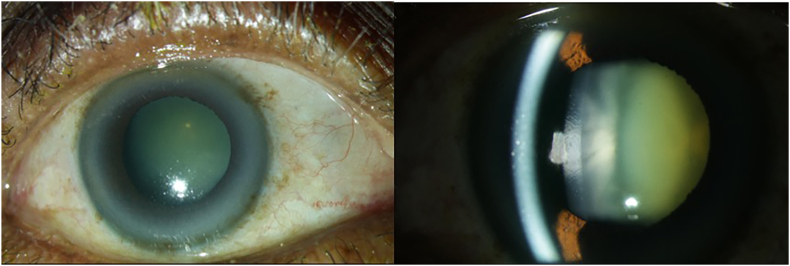
Cataract in an 83-year-old woman. The photograph of the anterior segment of the right eye shows the typical clinical appearance of cataract with corticonuclear lens opacification. Unpublished images. Department of Ophthalmology, University Medical Center Mainz.

Lens opacification, also termed cataract, is the most common cause of blindness worldwide, especially in non-industrial nations and rural areas [203]. The cataract itself can be roughly divided anatomically into different subtypes, which can also be mixed. A cataract of the lens cortex is termed cortical cataract and is often accompanied by hyperopia. Opacification of the lens nucleus is termed nuclear cataract and often results in myopia. Subcapsular cataract is located under the lens capsule and is often associated with a rapid decrease of visual acuity. Furthermore, cataract can be classified according to its etiology, for example into age-related, traumatic, diabetic, or radiation-related subtypes. The most common form of cataract is age-related nuclear cataract [204]. A clinical picture of a patient with cataract in presented in Fig. 7.

##### 2.3.1.2 Mechanisms of oxidative stress in cataract formation

During aging, lens cells lose the ability to synthesize or replace proteins, resulting in the deposition of proteins, such as crystallins, the main proteins in the lens, which contribute to the crucial function of maintaining transparency [205]. Age-related or oxidative processes can cause destabilization of crystallins with subsequent protein accumulation, which finally drives to a process of lens opacification, visible in age-related nuclear cataract [206]. This age-dependent process can be accelerated by exogenous influences, including UV radiation [207] or heavy metals [208]. An altered redox status, resulting in ROS excess, has been shown to profoundly contribute the pathogenesis of age-related nuclear cataract. Due to the long-term exposure of lens cells to UV radiation and consequently to oxidative stress, lens cells have developed high levels of antioxidants [209], repairing mechanisms for proteins [210] and nucleic acids [211].

The major antioxidant protein present in the lens is reduced GSH [212]. It is produced by the lens epithelium and is almost entirely present in its reduced form. Accordingly, oxidized glutathione (GSSG) is rapidly reduced to GSH. GSH preserves proteins and enzymes, including Na^+^/K^+^-ATPase, from oxidative damage. With increasing age, GSH synthesis and reduction of GSSG decreases [213,214]. The increasing rate of GSSG causes thiolation of proteins leading to the formation of disulfides and large protein conglomerates by further oxidation [215,216]. Large proteins are characterized by a high molecular weight and their size causes increased refraction of light and thus the formation of cataract. This metabolic process is the main step in cataract development. Large crystalline conglomerates held together by disulfide compounds have been detected in human lenses with cataract. Agents that can repair and restore these proteins to their original state are for example glutareoxin [217] and glyceraldehyde-3-phosphate dehydrogenase (G3PD) [218]. The activity of these enzymes decreases with age, indirectly favoring cataract formation. It has been demonstrated that GSH is relatively absent in the nucleus, favoring the primary development of age-related nuclear cataract [219]. Apart from the above-mentioned oxidative processes [81,220], non-oxidative metabolic processes such as deamination [221,222] or mutations of amino acids in proteins [223] may also play a role.

**Fig. 8:**
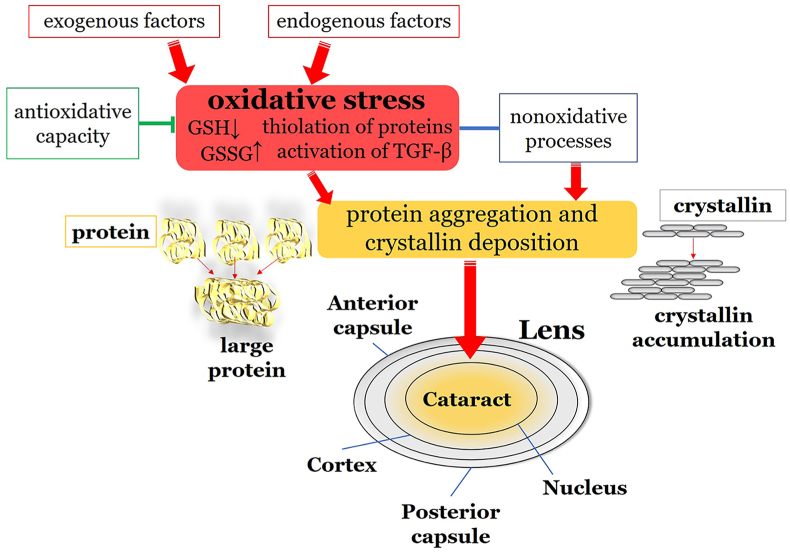
Role of oxidative stress in cataract formation. Exogenous factors, such as UV radiation, and endogenous factors, such as aging cause increased oxidative stress. This leads to crystallin deposition, ultimately resulting in the formation of large protein conglomerates causing the typical opacity of cataract and leading to loss of visual acuity. GSH: glutathione; GSSG: oxidized glutathione; TGF-β: transforming growth factor-beta.

Oxidative stress is one of the major contributors to the pathogenesis of fibrotic cataract forms, including posterior subcapsular cataract [224]. A distinctive feature in the formation of this type of cataract is the epithelial-mesenchymal transition (EMT) of lens epithelial cells [225,226]. In EMT, normal cuboid lens epithelial cells morphologically transform into spindle-shaped mesenchymal cells. In posterior subcapsular cataract, lens epithelial cells complete EMT and migrate posteriorly, where they form a dense fibrotic plaque, reducing vision. TGF-β has been shown to favor this transition by promoting migration, contraction, and production of extracellular matrix [226]. Oxidative stress favors the activation of TGF-β, further aggravating cataract formation [225]. In animal models, antioxidants such as GSH prevented TGF-β-mediated cataract formation [227]. Wang et al. assessed that extracellular vesicles promote EMT [228]. In this context, Thompson et al. postulated that oxidative stress triggers cytokine expression possibly leading to EMT and finally to posterior subcapsular cataract [229]. The role of oxidative stress in cataract formation is illustrated in Fig. 8.

##### 2.3.1.3 Therapeutic approaches for oxidative stress in cataract

Lifestyle modifications may represent the first step in the reduction of cataract development. A healthy lifestyle that includes a balanced diet, regular exercise, and avoiding smoking and excessive alcohol consumption can contribute to overall eye health and reduce the risk of cataract development [[230], [231], [232]]. Topical and systemic administration of antioxidants has been analyzed as a therapeutic approach in several oxidative stress-related ocular diseases. Ascorbic acid as radical scavenger and regulator of antioxidants with UV light-filtering effects may reduce the risk of cataract in patients with impaired antioxidant capacity or low plasma levels of ascorbic acid [233,234]. A meta-analysis assessed that supplementation of the antioxidant, vitamin E, is associated with a reduced risk for age-related cataract [235]. Other authors also reported positive effects of antioxidants, such as lutein, zeaxanthin or carotenoids in this context [[236], [237], [238]]. However, others found contrary results: A systematic review revealed that there is no evidence for supplementation with antioxidant enzymes, such as vitamin C, vitamin E or beta-carotenes to prevent or slow cataract progression [239]. Several other studies found no positive effects of vitamin supplementation with regard to cataract formation either [[240], [241], [242]].

As previously mentioned, Nfr2 is a nuclear transcription factor that can induce various antioxidant genes. Inducers of Nrf2, such as the acetyl ester of the trimethylated amino acid l‐carnitine (ALCAR), hyperoside, flavonoids such as morin, SFN or NBP stimulate antioxidative defense systems and have been shown to reduce cataract formation [243]. Melatonin was shown to inhibit ferroptosis and delayed age-related cataract formation by regulation of Nrf2 associated pathways [244]. Humanin as a mitochondrial-related peptide has a cytoprotective character and increases mitochondrial autophagy with consequent reduction of ROS production under oxidative stress conditions. Thereby, it may represent another interesting therapeutic approach for age-related cataract [245].

### 2.4 The retina

The retina is a well-structured part of the central nervous system converting incoming light into an electrical signal, which is transmitted to the brain to generate a complex visual output [246]. It is located at the posterior eye segment between the vitreous body and the choroid. The retina is formed by nine different layers: the internal limiting membrane, the nerve fiber layer, the ganglion cell layer, the inner plexiform layer, the inner nuclear layer, the outer plexiform layer, the outer nuclear layer, the external limiting membrane and the photoreceptor layer [3]. It is adjacent to the retinal pigment epithelium (RPE), a basement membrane on the choroidal side forming the outer blood-retina barrier. Moreover, the RPE plays a crucial role in recycling the chromophore for visual pigment, retinaldehyde, by phagocytosis of the outer photoreceptor segments [247]. The photoreceptors, rods and cones, are located in the outermost layer anterior to the RPE and contain light-sensitive pigment. When these receptors are stimulated by light, they undergo a chemical change with consequent generation of a chemical signal. This process is called phototransduction [247]. Signal processing is ensured by different cell types in inner retinal layers, such as bipolar cells, horizontal cells, amacrine cells and ganglion cells [247]. The final output cells of the retina are the retinal ganglion cells (RGCs) whose axons form the optic nerve to carry the visual information to the visual cortex [248].

The retina receives blood supply from two vascular beds: The inner retinal layers are perfused by vessels from the central retinal artery, whereas the outer retinal layers are supplied via diffusion from choroidal vessels. The central retinal artery is the first branch of the ophthalmic artery and enters the eye with the optic nerve. It branches into three different vascular layers: the superficial, intermediate, and deep vascular plexus [3,41]. RGCs are supplied by the superficial and intermediate capillary plexus and have been shown to provide important transcriptional factors for retinal angiogenesis, such as HIF-1α [249].

The retinal blood supply is regulated by autoregulatory processes, while choroidal blood flow is in part regulated by autonomic nerves [250]. Due to its high metabolic rate and high oxygen consumption, retinal tissue generates large amounts of ROS [36]. Especially, the retinal pigment epithelium needs stable redox homeostasis and obtains reparative systems of endogenous cell defense to maintain retinal functions [251]. NO generated by NOS is a very powerful vasodilatory factor. It uses l-arginine as a substrate and needs tetrahydrobiopterin (BH4) as a cofactor. Under oxidative conditions, this important regulator of NOS function may become depleted leading to NOS uncoupling and aggravation of ROS generation [252]. Consecutive endothelial dysfunction impairs blood flow regulation and initiates a proinflammatory and prothrombotic state, which is a basis for various retinal diseases [3].

#### 2.4.1 Age-related macular degeneration

##### 2.4.1.1 Clinical insights into age-related macular degeneration

AMD is a leading cause of blindness in the elderly population. In 2020, AMD ranked fourth among common causes of blindness worldwide in people over 50 years (with 5.4% of 33.6 million blind adults) after cataract, glaucoma, and under-corrected refractive error. The prevalence of AMD is expected to increase even further due to the rising average life expectancy with 288 million expected patients in 2040 [253,254]. Many risk factors for AMD have been found, such as ethnicity, genetics, gender, environmental factors (like high fat diet, sunlight exposure and smoking) and, what is eponymous for the disease, age [255]. Genetic variants like mtDNA polymorphisms, CFH-related proteins, apolipoprotein E and complement factor 2,3,9,I and B are associated with AMD [256].

**Fig. 9:**
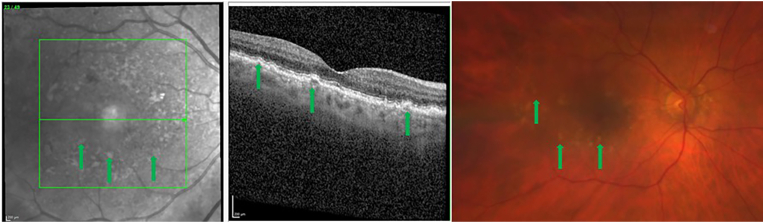
Non-neovascular age-related macular degeneration in an 80-year-old woman. Optical coherence tomography and fundus photograph of a patient with nonvascular (dry) age related macular degeneration are shown. The green arrows point to drusen, that appear as focal yellow extracellular deposits within and around the macula. Unpublished image. Department of Ophthalmology, University Medical Center Mainz. (For interpretation of the references to colour in this figure legend, the reader is referred to the Web version of this article.)

AMD is characterized by a progressive degeneration of the central retina and can be classified into three clinical stages: early, intermediate and advanced [257]. In early and intermediate stages, small or medium sized drusen, focal extracellular yellow deposits, are visible. Central vision loss appears in advanced stages that can be subdivided into non-neovascular (“dry”) AMD and neovascular (“wet” or exudative) AMD. In advanced non-neovascular AMD, geographic atrophy that involves the central macula or fovea is the predominant feature and causes usually slowly progressing vision loss. Geographic atrophy is a progressive deterioration of the RPE, photoreceptors and macular choroidal capillaries [257]. Optical coherence tomography and a fundus photograph of a patient with non-neovascular AMD are shown in Fig. 9. Neovascular AMD is defined by choroidal neovascularization (CNV) and increased expression of VEFG, which lead to hemorrhage and leakage of fluid into the inner retinal layers or subretinal space. A rapid progression with loss of central vision is typical [256,257]. Intravitreal application of anti-VEGF agents is the main treatment option for neovascular AMD. However, laser therapy like photodynamic therapy can be used in addition [258].

##### 2.4.1.2 Mechanisms of oxidative stress in age-related macular degeneration

The retina stands out as one of the tissues with the highest oxygen-consumption in the human body. Of note, the outer retina is supplied with oxygen from the choroidal circulation whereas the inner retina is dependent on the retinal vasculature. However, the central 250–600 μm of the macula do not possess any retinal vessels and are exclusively fueled by the underlying choriocapillaris. Thereby, the RPE is exposed to high levels of oxygen that promote ROS production, such as O_2_^•−^, ^•^OH or H_2_O_2_ [256,259]. Under physiological conditions, significant amounts of ROS are generated, which are essential for physiological cell functions [259]. Certainly, an imbalance between generated ROS and the antioxidant capacity of the macula in favor of oxidative stress can cause RPE cell death. Excessive generation of ROS can trigger the MAPKs and NF-κB-dependent signaling pathways and therefore elevated levels of inflammatory cytokines, such as IL-1β, IL-18 and TNF-α via the NLRP3 inflammasome and caspase-1 [256]. Additionally, ROS may be linked to an increase of VEGF levels via HIF-α and a decrease of vasoprotective NO levels, contributing to choroidal vascular dysfunction [256,260].

Different risk factors may promote ROS generation in patients suffering from AMD. Aging is associated with oxidative modifications of macromolecules. An increased generation of ROS may be due to a rising leakage of electrons from the electron transport chain and an accompanied by a reduction of the antioxidant defense capacity [61,261].

Another factor causing increased ROS production and therefore photooxidative stress is the exposure to UV radiation and blue light [262]. For example, a dose-dependent reduction in cell viability to UVB light exposure with simultaneously increased ROS levels has been observed in an in vitro cell-based model. Furthermore, expression of the apoptotic marker Bax was increased and of the antiapoptotic marker Bcl-2 decreased [263]. However, anterior structures of the eye filter most parts of the UV spectrum and only a portion of the UVA band penetrates into the retina in adults [264]. The retinal absorption of UV radiation results in photochemical reactions via a type 1 mechanism (direct free radical reactions) and a type 2 mechanism (reactions involving ROS) [264].

Additionally, UV exposure upregulates proinflammatory molecules via NF-κB activation and decreases ATP generation. Lower ATP levels could be responsible for a less effective RPE phagocytosis resulting in a RPE hyperpigmentation, a potential risk factor for AMD [261]. In another cell-based model, increased ROS production by enhanced NOX activity and mitochondria-like activity especially in the outer segments of photoreceptors has been observed after blue light radiation [265].

Moreover, a rhodopsin-dependent toxic effect of blue light on the retina has been reported, suggesting that reduced exposition might decrease the risk for retinal diseases such as AMD [266]. However, large epidemiologic studies evaluating the effects of blue light blocking intraocular lenses did not show any benefits [267].

One of the most significant environmental risk factors for AMD is cigarette smoking, as shown in numerous epidemiologic studies like the Blue Mountain Eye Study [255,259]. Likewise, direct injury of RPE cells via cigarette smoke-induced oxidative stress has been demonstrated in mice, which developed RPE apoptosis and basal drusen-like deposits after an exposition of 6 months [261]. As part of the comprehensive cytoprotective response in RPE cells, cigarette smoke triggers the activation of the antioxidant transcription factor Nrf2 and upregulates autophagy related genes, including p62 [268]. Interestingly, Zhao et al. detected ocular pathologies similar to human AMD in Nrf2-deficient mice [269]. Nrf2 extenuates oxidative injury and supports the maintenance of cellular redox homeostasis [270]. Without surrounding stress, it gets tagged by Kelch-like ECH-associated protein 1 (Keap1) and then decomposed in the proteasome. However, when p62 is upregulated, it competes with Nrf2 for Keap1 interaction which prolongs the action time of Nrf2 and therefore increases the cellular antioxidant capacity. Notably, p62 plays an important role in autophagy of aggregated proteins through its ubiquitin-associated (UBA) domain [268]. Impaired autophagy in RPE appears to profoundly contribute to the pathogenesis of AMD, because an ineffective removal of oxidatively impaired proteins and vulnerable fatty acids can further exacerbate oxidative stress [256].

##### 2.4.1.3 Therapeutic approaches targeting oxidative stress in age-related macular degeneration

Due to the significant role of oxidative stress in the development of AMD and the limited treatment options for non-neovascular AMD, therapeutic strategies targeting oxidative stress may hold promise for AMD treatment in addition to established methods.

Anti-VEGF therapy is a standard treatment for neovascular AMD to reduce the risk of vision loss [271]. Apart from its direct effects like suppression of endothelial cell proliferation, formation of choroidal neovascularization and vascular permeability, there is evidence that vascular dysfunction and ROS are directly and indirectly connected. For example, a direct link between VEGF and elevated levels of ROS has been discovered in cultured human choroidal endothelial cells. In the study, VEGF induced activation of Rac1, a part of NOX, and NOX inhibitors lead to a decline of ROS generation [260]. Multiple subtypes of NOX have been shown to generate ROS, leading to elevated VEGF levels [260]. Combination of anti-VEGF therapy and inhibition of angiopoietin-2 with faricimab represents a new promising therapeutic option in neovascular AMD [272]. Reduced inflammation and vascular permeability have been detected in a murine model of retinal neovascularization and ischemia/reperfusion after dual inhibition of angiopoietin-2 and VEGF [273].

Apart from anti-VEGF therapy, conservative treatment options are another method to suppress oxidative stress. Different risk factors like high fat diet, sunlight exposure and smoking should be reduced or eliminated to minimize oxidative stress [255]. Well-studied supplements are the carotenoids lutein and zeaxanthin. The Age-Related Eye Disease Study (AREDS) reported a reduced risk of AMD progression following daily supplementation of 500 mg vitamin C, 400 IU vitamin E, 2 mg cupric oxide, 80 mg zinc, and 15 mg β-carotene. While other studies supported these findings, some large prospective studies, like the Beaver Dam Study, did not find any evidence for a benefit of antioxidant supplementation or only found it in specific subgroup analyses [274].

Additionally, p62 and Nrf2 have emerged as potential targets for a novel therapeutic approach in AMD. Various nutritional supplements have been identified for their ability to activate or induce Nrf2 and its downstream genes [261]. For instance, sulforaphane, found in broccoli and cabbages, increased GSH levels and the expression of the antioxidant protein thioredoxin, which is regulated by Nrf2, as well as NOX activity in murine models [275]. Similarly, curcumin, a component of curcuma, induced the Nrf2-driven gene HO1, while analogs like carnosic acid (found in rosemary and sage), salvianolic acids (from sage), mangostin (from mangostan), and taxifolin (from conifers) all induced Nrf2 itself [261]. Another conservative treatment option are statins which may have a potential protective ability by increasing eNOS and potentially downregulating VEGF expression and ROS generation, but large prospective studies are not available to this date [276,277].

#### 2.4.2 Diabetic retinopathy

##### 2.4.2.1 Clinical presentation of diabetic retinopathy

**Fig. 10:**
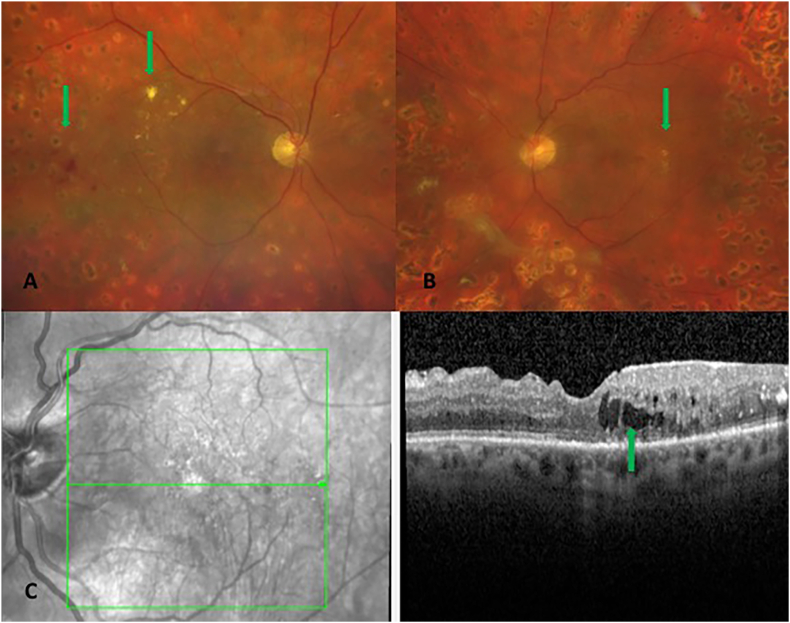
Non-proliferative diabetic retinopathy in a 70-year old woman. Fundus photographs of both eyes and optical coherence tomography of left eye in a patient with severe non-proliferative diabetic retinopathy are shown. The green arrows point to intraretinal hemorrhages and hard exsudates around the macula. Optical coherence tomography demonstrates diabetic macular edema. Unpublished image. Department of Ophthalmology, University Medical Center Mainz. (For interpretation of the references to colour in this figure legend, the reader is referred to the Web version of this article.)

DR is a common microvascular complication of diabetes mellitus and a leading cause of vision loss. It ranks as the fifth most common cause of blindness in adults over 50 years [253,278]. The number of patients with diabetes mellitus was approximately 463 million in 2019, and is expected to reach 700 million by 2045 [279]. About 45% of these patients have DR, and 22% have vision-threatening DR [279,280]. Persistent hyperglycemia leads to increased oxidative and ER stress, inflammation, and dysregulated autophagy, resulting in significant damage to retinal tissue [281]. Most patients do not experience noticeable symptoms before developing severe complications, underscoring the importance of a systematic screening program [278]. DR can be clinically classified into different stages based on fundus examination. It is essential to distinguish between the non-proliferative and the proliferative form. Proliferative disease is characterized by new blood vessels formation and/or vitreous or preretinal hemorrhage, while non-proliferative DR presents with microaneurysms, intraretinal hemorrhages, or intraretinal microvascular abnormalities without signs of proliferation. Non-proliferative DR can further be subdivided clinically into mild, moderate, and severe subtypes. In addition to hemorrhages, a common complication of DR is diabetic macular edema. Both can lead to severe vision loss [282]. Optimal management of DR requires strict control of blood glucose levels and other cardiovascular risk factors, such as blood pressure and serum cholesterol levels [278]. Standard ophthalmologic treatment includes panretinal laser photocoagulation for proliferative DR, which may have side effects such as visual-field constraints. Intravitreal anti-VEGF or steroid injections for diabetic macular edema are well-established and can also be used as treatment options [283]. A fundus photograph of a patient with DR is shown in Fig. 10.

##### 2.4.2.2 Mechanisms of oxidative stress in diabetic retinopathy

Hyperglycemia, the primary factor contributing to the pathogenesis of diabetes-related complications like DR, leads to increased ROS levels via activation of multiple signaling pathways [284]. First, the polyol pathway converts glucose into sorbitol and then into fructose. This process requires NADPH and NAD^+^. Since NADPH is usually involved in the regeneration of the antioxidant GSH, a decrease in NADPH availability results in a reduced antioxidant capacity [285]. Additionally, accumulation of sorbitol and fructose increases osmotic pressure, leading to membrane permeability and edema [284]. Second, the hexosamine pathway converts glucose to N-acetlyglucosamine 6-phosphate, which, similar to the polyol pathway, decreases NADPH-dependent GSH production, resulting in increased H_2_O_2_ levels [286]. Third, AGEs are nonenzymatically glycosylated macromolecules formed under hyperglycemic conditions playing a crucial role in ROS generation. Activation of RAGE, the receptor for AGEs, initiates enhanced ROS generation and various other effects, such as cytokine release and microvascular complications [3,287]. Moreover, the PKC pathway gets activated via high ROS levels in hyperglycemia due to the described biochemical mechanisms. The activated PKC pathway further increases ROS generation via NOX activation [286]. Due to further promotion of ROS generation, the bioavailability of NO becomes impaired consequently increasing endothelial permeability and VEGF expression [3].

At the cellular level, hyperglycemia triggers mitochondrial dysfunction. Elevated glucose levels disrupt the proton gradient and the mitochondrial electron transport chain, leading to the formation of superoxide through coenzyme Q. In experimental animals with DR, increased levels of superoxide have been observed along with a reduced expression of mitochondrial SOD. Increased superoxide and ROS levels are linked to augmented swelling, permeability, mitochondrial DNA damage and consequently retinal neuron apoptosis in DR models [288,289].

**Fig. 11:**
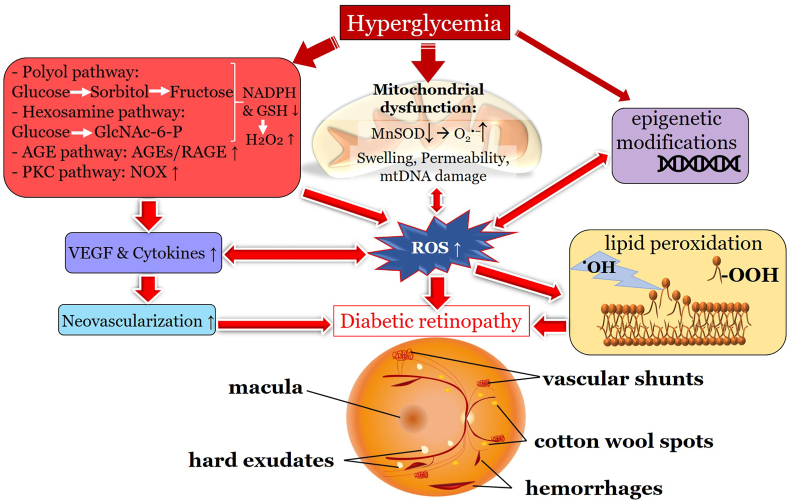
Source and role of oxidative stress in diabetic retinopathy. Hyperglycemia and epigenetic modifications induce mitochondrial dysfunction and metabolic pathways, such as polyol, hexosamine, PKC pathways and AGEs leading elevated levels of ROS. Elevated levels of ROS cause lipid peroxidation and neovascularization through elevated VEGF and cytokine levels, finally terminating in diabetic retinopathy. AGEs: Advanced Glycation End Products; PKC: Protein Kinase C; VEGF: vascular endothelial growth factor; ROS: reactive oxygen species; NADPH: reduced form of nicotinamide adenine dinucleotide; GSH: glutathione; H_2_O_2:_ hydroxyl peroxide; GlcNAc-6-P: N-acetlyglucosamine 6-phosphate; MnSOD: mitochondrial superoxide dismutase; O_2_^•−^: superoxide.

In addition to these metabolic abnormalities, chronic hyperglycemia can induce epigenetic modifications, that persist even after comprehensive antidiabetic therapy and glucose level normalization. This phenomenon is referred to as “metabolic memory” [290]. Epigenetic changes in DR promote upregulation of inflammatory proteins and inhibition of ROS scavenging genes through histone methylation or acetylation, modulation of transcription factors, or aberrant expression of regulatory non-coding RNAs [291]. For example, epigenetic modifications involving increased methyl H4K20 and acetyl H3K9, as well as LSD1-mediated elevated demethylation in H3K4, can downregulate SOD2 and promote apoptosis [291]. Another noteworthy epigenetic alteration involves the heightened expression of Keap1, driven by the promoter SetD7 due to hyperglycemia [292]. Keap1 hinders Nrf2 activity, which is critical for the antioxidant defense. Therefore, Keap1 and reduced antioxidant capacities play pivotal roles not only in AMD but also in the pathogenesis of DR [268]. Interestingly, not only chronic hyperglycemia but also intensely fluctuating glucose levels appear to be strong predictors of diabetic vascular complications. In human retinal endothelial cells cultured in vitro, intermittent high glucose levels trigger greater ROS generation and VEGF expression compared to sustained high glucose levels [293]. A scheme of sources and the role of oxidative stress in the pathogenesis of DR is shown in Fig. 11.

All these pathways lead to elevated oxidative stress and, along with hyperglycemia itself, inflict structural and functional damage in retinal cells. Lipid peroxidation, associated with elevated oxidative stress levels, exacerbates the severity of diabetes. This process compromises the integrity of cellular membranes and augments the generation of diffusible aldehydic by-products like HNE and HHE [288]. HNE can initiate p53-mediated apoptosis in RPE cells, while HHE can stimulate NF-κB-associated inflammatory pathways and disrupt mitochondrial membrane permeability [294,295].

Oxidative stress can directly trigger mitochondrial damage as well. Circular mtDNA is more vulnerable to oxidative stress than nuclear DNA due to the absence of protective histones. Damaged mtDNA enhances ROS generation through inaccurate protein synthesis, compromising electron transport [291]. Elevated oxidative stress within mitochondria increases pore permeability, leading to the release of cytochrome *c* and other proapoptotic factors like NF-κB. Cytochrome *c* indirectly activates caspase-3 via caspase-9. Activation of caspases and proapoptotic factors contributes to inflammation and initiates cellular apoptosis [288]. In an animal model, the inhibition of cytochrome *c* suppressed the oxidative stress-associated pathogenesis of DR [296]. Under oxidative stress conditions, alterations in uncoupling proteins have been noted. Uncoupling proteins offer a potential means to positively influence the electron transport chain in mitochondria under oxidative stress conditions. They increase proton leakage across the inner mitochondrial membrane, thereby reducing ROS generation [284]. Through elevated oxidative processes under diabetic conditions, cell injury in both, retinal blood vessels and neurons occurs with the induction of a vicious circle between neuronal and vascular dysfunction [3].

##### 2.4.2.3 Therapeutic approaches for oxidative stress in diabetic retinopathy

This mechanistic insight presents an encouraging therapeutic avenue in the management of DR.

For example, PPAR agonists, commonly employed in oral diabetic treatments, have been shown to decrease ROS production and cell death in cultured retinal neurons [297]. A natural compound that might activate UCP2 is curcumin, which is also known for its interaction with the Nrf2 pathway [261,298]. However, the limited bioavailability of curcumin remains a challenge for its potential therapeutic benefits in various retinal diseases [298].

Numerous other natural antioxidants or activators of antioxidant defense systems, such as lutein, zeaxanthin, sulforaphane, and curcumin, have been investigated for their therapeutic potential in DR and other retinal diseases. Some studies have reported that antioxidants can influence gene expression through epigenetic modifications. Polyphenols like epigallocatechin-3-gallate (EGCG), found in green tea, have been studied for their modulation of oxidative stress in DR. EGCG possesses diverse antioxidant capabilities: It enhances the activity of GPX and SOD and acts as a scavenger for peroxynitrite and free radical byproducts like hypochlorite and peroxyl radicals. EGCG also has metal-chelating properties, reducing the free forms of metals such as iron and copper, which subsequently curtails ROS reactions [299]. Additionally, it suppresses NF-κB activity, diminishes AGE formation, and hampers collagen cross-linking [300]. Another notable polyphenol is resveratrol, which can mitigate oxidative stress through multiple mechanisms. It functions as a ROS scavenger and thus holds potential protective effects across various ocular diseases. Particularly relevant to DR, resveratrol can inhibit the PKC pathway, which generates excessive amounts of ROS under hyperglycemic conditions. Furthermore, it can restore insulin levels and promote the expression of paraoxonase 1, an enzyme that safeguards lipoproteins from oxidation. Numerous other antioxidants, such as sulodexide, astaxanthin, lipoic acid, various vitamins (A, B1, C, E, D), and their derivatives like benfotiamine, are recognized for their favorable impact on oxidative stress [288,301]. However, more extensive clinical studies are needed to assess their potential contribution to the treatment of diabetes mellitus and DR in the future.

#### 2.4.3 Retinal vascular occlusion

##### 2.4.3.1 Clinical insight into retinal vascular occlusion

There are two different types of vessel occlusion in the retina: retinal artery and vein occlusion. Retinal artery occlusion is an ophthalmologic emergency with sudden severe vision loss due to sudden cessation of retinal blood supply [302]. The estimated incidence is about 1 in 100,000 people [303]. It is associated with an elevated risk of stroke and other cardiovascular events, since the major cause of retinal artery occlusion are emboli from the heart, aortic arch, carotid artery or systemic vasculitis [41]. Thereby, acute disease management and checkup for risk factors is necessary. Due to interruption of the retinal perfusion, retinal ischemia with irreversible cell death may occur. During fundus examination appearance of retinal opacity in the posterior pole, cherry-red spot, cattle trucking, retinal artery attenuation, optic disc edema and pallor are typical. Further diagnostic steps are optical coherence tomography (OCT), fluorescent angiography and assessment of cardiovascular risk factors, for example by duplex carotid ultrasound [303]. Clinical complications are permanent vision loss, retinal or vitreous hemorrhage, neovascularizations or neovascular glaucoma [3]. Therapeutic options are systemic or arterial fibrinolysis, but improvement of outcome has been questioned [304]. Conservative treatment options, such as methylprednisolone, ocular massage or acetylsalicylic acid, showed also low effects for outcome [303].

**Fig. 12:**
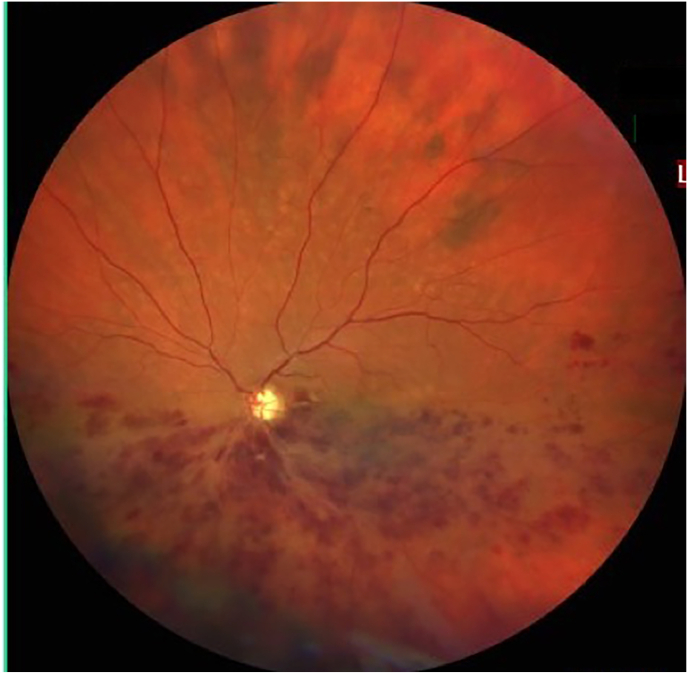
Branch retinal vein occlusion in a 62-year-old patient. Fundus photograph of a left eye with retinal vein occlusion in the inferior branch of central artery is demonstrated. Retinal occlusion leads to dilatation and tortuosity of retinal veins. Retinal hemorrhage as well as macular edema are demonstrated. Unpublished image. Department of Ophthalmology, University Medical Center Mainz.

Retinal vein occlusion is also a widespread cause of vision loss that is associated with aging, atherosclerosis and cardiovascular risk factors [41]. It is caused by thrombotic occlusion of central retinal vein or branches of retinal veins. Consequent increased pressure of the capillary bed leads to elevated retinal capillary permeability and leakage. Retinal ischemia promotes the production of VEGF causing neovascular complications [305]. Clinically, retinal vein occlusion leads to dilatation and tortuosity of the retinal veins. Moreover, retinal and subretinal hemorrhage as well as macular edema occur. Next to fundus examination, optical coherence tomography and fluorescein angiography are important diagnostic features [306]. In case of macular edema, intravitreal medication with anti-VEGF drugs or steroids is important. When the retina gets ischemic, panretinal photocoagulation is mandatory [305]. A fundus photograph of a patient with branch retinal vein occlusion is demonstrated in Fig. 12.

##### 2.4.3.2 Mechanisms of oxidative stress in retinal vascular occlusion

Both, retinal artery and vein occlusion are associated with retinal ischemia or ischemia/reperfusion injury. There are several experimental models of retinal or ocular ischemia that aim to analyze involved molecular pathways in this context. For example, in an ocular ischemia/reperfusion model in pigs where an ischemic period of 12 min was followed by 20 h of reperfusion, RGC loss, endothelial dysfunction and oxidative stress in retinal arterioles were observed together with upregulation of HIF-1α, VEGF-A, and NOX2 [307]. Likewise, in a mouse model of ocular ischemia/reperfusion, RGC loss, vascular endothelial dysfunction, elevated ROS levels and NOX2 upregulation were observed in the retina [308,309]. Genetic deletion of NOX2 in mice reduced RGC death after ischemic injury, indicating that ROS and NOX2-associated pathways may play a role in ischemic cell death in the retina [310]. After induction of retinal ischemia by central retinal artery ligation, apoptosis related genes, including Tp53, Akt1, Akt3, Pi3R1, caspases and TNF were upregulated [311]. Induction of necrosis and apoptosis after induction of ocular/retinal ischemia was demonstrated by various studies in different disease models [312,313].

Nrf2 is a major regulator of antioxidant enzymes. Nrf2 knockout mice exhibited an increased loss of RGCs after induction of retinal ischemia. In the same study, induction of ROS generation was inhibited by Nrf2 activation while survival of RGCs was increased [314].

Inflammation is also associated with oxidative stress, and toll-like receptor 2 and 4 (TLR2 and TLR4) were reported to be involved in the induction of apoptosis during ischemic retinal injury [315]. Myeloid differentiation protein2 has been identified to activate TLRs, and participation in TLR4-NOX4 complex formation may contribute to oxidative and inflammatory damage [316]. Moreover, the TLR2/TLR4/STAT3/NF-κB pathway was shown to be involved in the generation of ROS in a model of ischemia-reperfusion injury in the rat retina [315]. These experimental findings suggest the involvement of oxidative stress in retinal ischemia.

In patients with retinal vein occlusion, elevated serum oxidative stress marker levels, such as MDA, 8OHdG or H_2_O_2_ were detected, while scavenging enzymes for ROS, such as SOD, were decreased [317]. The oxidative stress index in the aqueous humor of patients with retinal vein occlusion was significantly higher compared to healthy controls [318]. In patients with retinal vein occlusion, erythrocyte–derived ROS and lipid peroxidation were elevated and positively correlated with whole blood viscosity and erythrocyte deformability [319]. High plasma levels of homocysteine represent a risk factor for central retinal vein occlusion in young adults due to potential toxicity to the vascular endothelium. A positive correlation between homocysteine levels and the oxidative stress marker, thiobarbituric acid-reacting substance, was detected in patients with retinal vein occlusion [320].

Moreover, in patients with retinal vein occlusion, vitreous levels of cytokines, such as IL-6, were significantly elevated in patients with the ischemic type compared to patients with a non-ischemic type [321]. Moreover, hypoxia and oxidative stress stimulate vascular endothelial cells to produce IL-8, which is a powerful chemoattractant for neutrophils and T-cells. Elevated levels of IL-8 were found in the vitreous fluid in ischemic ocular diseases, such as central retinal vein occlusion and DR [322]. The chemokine increases endothelial permeability by downregulation of tight junctions [323]. Ischemia is also associated with upregulation of VEGF, and higher levels of VEGFR-1 and VEGFR-2 were found in the aqueous humor of patients with central retinal vein occlusion. Activation of VEGFR-1 further promotes inflammation by increasing the expression of inflammatory enzymes, such as MCP-1 and ICAM-1 via NF-κB and thereby aggravating ROS generation [324].

##### 2.4.3.3 Therapeutic approaches for oxidative stress in retinal vessel occlusion

Pioglitazone reduced NF-κB activation and apoptosis in a rat retinal ischemia/reperfusion injury model [325]. Diminished oxidative stress and inflammation were also detected by application of chitosan oligosaccharides via modulation of NF-κB, JNK, ERK and p38 [326]. The NF-κB pathway was also suppressed in the retina by octreotide, a somatostatin analogue, in a murine ocular ischemia/reperfusion injury model [327].

Retinal ischemia/reperfusion has been shown to induce necroptosis, apoptosis and ferroptosis linked to iron deposition and lipid peroxidation. Inhibitors of apoptosis, necroptosis and ferroptosis, such as z-VAD-MFK, necrostatin-1 and ferrostatin-1, protected RGCs after retinal ischemia/reperfusion [328]. The protection of rat RGCs from ischemia/reperfusion injury was also demonstrated by mitochondrial μ-calpain [329], and inhibitors of the cJun N-terminal kinase [330]. Intraperitoneal injection of granulocyte colony-stimulating factor also protected from RGC death by phosphorylation and activation of AKT [331].

In mice, betulinic acid partially prevented vascular endothelial dysfunction and ROS formation in the retina after ischemia/reperfusion injury by boosting the expression of the antioxidant enzymes SOD3 and HO1 [308]. Resveratrol reduced the expression of the prooxidant enzyme NOX2 and reduced nitro-oxidative stress in the same animal model [309]. Dietary supplementation with the ROS scavenger coenzyme Q10 blocked apoptosis by decreasing Bax protein expression after ischemic injury [332]. Systemic treatment with brimonidine preserved mitochondrial function and protected against glutamate excitotoxicity-induced oxidative stress [333]. Nrf2 as an activator of antioxidant enzymes represents also a promising therapeutic target for retinal ischemia. Nrf2-activating drugs, such as triterpenoids, inhibitors of the Keap1-Nrf2 protein-protein or monomethyl fumarate reduced oxidative stress and RGC death during retinal ischemia [314,334,335].

Intravitreal treatment of macular edema in patients with retinal vein occlusion is a well-established therapy. Anti-VEGF therapy reduces inflammation, angiogenesis and ROS generation by inhibition of VEGF, ICAM-1, MIP-1, NOX-1 and NOX-4 in these patients [336]. Next to anti-VEGF, intravitreal steroid application is also an established therapeutic option. In a rabbit retinal vein occlusion model, upregulation of TNF-α and NF-κB related pathways was detected to play a crucial role during inflammation and angiogenesis [337]. Steroids, such as dexamethasone, have anti-inflammatory potential by reducing the expression of transcription factors, such as NF-κB. Moreover, upregulation of Nrf2 and other antioxidant factors was detected after treatment with dexamethasone [338,339].

#### 2.4.4 Retinitis pigmentosa

##### 2.4.4.1 Clinical insights into retinitis pigmentosa

Retinitis pigmentosa stands as the most prevalent inherited retinal degenerative disorder, affecting approximately 1 in 4000 individuals globally and emerging as a leading cause of blindness among younger patients [340]. This condition comprises a diverse spectrum of hereditary ocular disorders, encompassing around 90 identified genes with thousands of mutations nestled within them. In addition to genetic and allelic variability, distinct mutations within the same gene can lead to disparate diseases (phenotypic heterogeneity), while a singular mutation might result in an array of clinical outcomes (clinical heterogeneity) [341]. The underpinning of retinitis pigmentosa lies in inherited or spontaneous mutations, culminating in a gradual demise of photoreceptor cells (predominantly rods) and concurrent RPE deterioration [342].

**Fig. 13:**
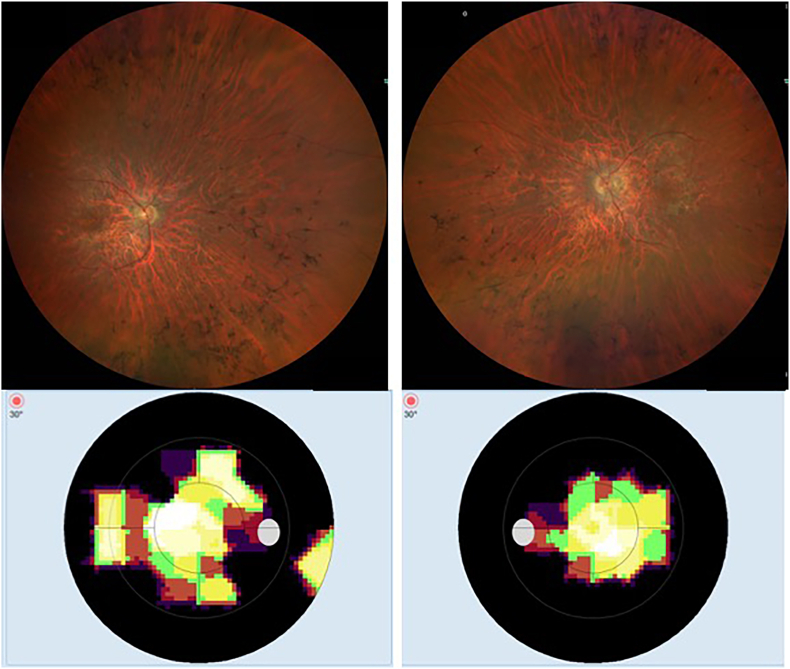
Retinitis pigmentosa in a 42-year-old patient. Fundus photograph und perimetry of both eyes with retinitis pigmentosa are demonstrated. Typical clinical signs, such as bone spicule-shaped pigment deposits, retinal atrophy, attenuation of retinal vessels, and a pale appearance of the optic nerve head can be detected. Perimetry shows typical concentric visual field loss with consequent tunnel vision. Unpublished image. Department of Ophthalmology, University Medical Center Mainz.

The hallmark of retinitis pigmentosa is the initial degeneration of photoreceptor rods, accompanied by subsequent disruption of cones. Consequently, the earliest symptom typically manifests as night blindness (nyctalopia), often noticed during adolescence, followed by peripheral visual field impairment and eventual tunnel vision, ultimately leading to central visual acuity loss [343]. The diverse array of genetic factors contributes to the varying age of onset, ranging from early childhood to mid-adulthood, along with different rates of progression [344]. Clinical signs encompass bone spicule-shaped pigment deposits, retinal atrophy, attenuation of retinal vessels, and a pale appearance of the optic nerve head [343]. Ocular manifestations further include cystoid macular edema and the formation of epiretinal membranes [345,346]. Diagnostic tools such as fundus evaluation, perimetry, and optical coherence tomography (OCT) are complemented by the full-field electroretinogram (ERG), a pivotal test especially useful in asymptomatic or mildly symptomatic patients, revealing hypovolted traces as a consequence of cellular degeneration [343]. Fundus photography and visual field examination of a patient with retinitis pigmentosa are demonstrated in Fig. 13.

Treatment avenues for retinitis pigmentosa can be classified into gene- or mutation-specific approaches and mutation-independent strategies. Gene-specific interventions require target cells, being most effective in the early stages with less cellular degeneration. Viral vectors are used to deliver wild-type copies of the complementary DNA (cDNA) corresponding to the mutated gene, restoring normal gene expressions. A significant breakthrough is the FDA-approved gene therapy, voretigene neparvovec (Luxturna), for RPE65 gene augmentation therapy, marking a milestone in retinal disease treatment [347].

Conventional treatments compromise dietary supplementation of vitamin A, docosahexaenoic acid (DHA), and lutein, along with sunlight protection [348]. An independent approach, unrelated to gene mutations, involves cell-based therapy, where stem cells hold the potential to restore dysfunctional cells or release tropic factors, facilitating restoration [348].

##### 2.4.4.2 Mechanisms of oxidative stress in retinitis pigmentosa

Experimental investigations in murine models of retinitis pigmentosa revealed elevated levels of retinal ROS. For instance, in rd1 mice with mutated Pde6b and Gpr179 genes, increased ROS levels were detected using dihydroethidium (DHE), a fluorescent probe that reacts with ROS, producing a signal in the outer retina absent in healthy retinas. Furthermore, specific antibodies have been employed to visualize oxidized lipids, proteins, and nucleic acids, particularly within the photoreceptor layer, across multiple models [349]. In human studies, ROS levels have been examined in aqueous humor, vitreous body, and peripheral blood samples. For instance, increased levels of 8-oxo-7,8-dihydro-2′-deoxyguanosine (8-oxo-dG), a marker for oxidative DNA damage, were observed in the vitreous humor, while antioxidant molecules like extracellular SOD3 and GSH, a crucial antioxidant, were diminished in aqueous humor samples [350,351].

Several factors are involved to the elevation of ROS levels in retinitis pigmentosa. As retinitis pigmentosa progresses, the degeneration of rods commences. Given that rods constitute approximately 95% of all photoreceptor cells in the outer retina, their oxygen consumption drastically declines. This reduction in oxygen consumption coupled with the persistently high oxygen exposure of remaining cones fosters heightened ROS production [352,353]. Cones, in turn, face oxidative stress surpassing their antioxidant capacity, contributing to cone cell demise [351].

Elevated oxygen tissue levels foster the accumulation of superoxide radicals, resulting from a mismatch in the mitochondrial electron transport chain and the stimulation of cytoplasmic NOX activity [354]. The electron transport chain generates superoxide radicals and becomes more efficient either when electron flow decreases, leading to a relative rise in electron donors, or when oxygen levels increase [355]. NOX facilitates the transfer of electrons across the plasma membrane towards molecular oxygen, generating the superoxide anion and consequently ROS, including H_2_O_2_ and hydroxyl radicals [356]. Elevated oxygen availability in hyperoxic states bolsters ROS generation through NOX activity [355].

Certain mutations contribute to increased ROS levels in specific patients. Some forms of retinitis pigmentosa are linked to inactivated genes responsible for parts of the endogenous antioxidant defense system, rendering these patients more susceptible to heightened ROS accumulation [357]. For instance, mutations in the CERKL (ceramide-kinase like) gene, which normally protects cells from oxidative stress and apoptosis, can exacerbate oxidative stress in certain autosomal-recessive forms of retinitis pigmentosa [358]. Additionally, specific retinitis pigmentosa-associated mutations can lead to the accumulation of unfolded or misfolded proteins within cells, triggering the unfolded protein response (UPR). This response, characterized by dysfunctional autophagy, calcium overload, and oxidative stress via PERK and IRE1 related pathways, can set in motion pro-apoptotic programs [359].

After highlighting different pathways that lead to increased oxidative stress it is essential to study the mechanisms resulting in retinal damage in retinitis pigmentosa to display possible therapeutic targets. As mentioned earlier, oxidized lipids, proteins and nucleic acids are increased in retinal probes [349]. Oxidative stress has been implicated as a primary cause of cone cell death, contributing to the decline of central visual acuity [352]. Retinitis pigmentosa not only leads to rod and cone cell death but also entails RPE degeneration. RPE cells are critical for maintaining retinal health, engaging in activities like daily phagocytosis of photoreceptor outer segments, light absorption, heat exchange, and vitamin A metabolism. Due to their heightened metabolic activity, RPE cells harbor an abundance of mitochondria and consequently represent a major source of ROS [360]. Furthermore, the daily phagocytosis of photoreceptor outer segments, which contain elevated ROS levels, leads to the accumulation of additional ROS within RPE cells [361]. This excessive ROS buildup significantly contributes to RPE degeneration [361]. Increased ROS levels have also been shown to upregulate VEGF, reduce the production of vasoactive factors like endothelial-derived NO, and trigger microvascular blood-retinal barrier remodeling. Consequently, elevated ROS levels encourage fluid leakage, fostering the development of cystoid macular edema, which occurs in 10–50% of retinitis pigmentosa patients and is a major contributor to central vision loss [293,346].

##### 2.4.4.3 Therapeutic approaches targeting oxidative stress in retinitis pigmentosa

Given the critical role of oxidative stress in the pathophysiology of retinitis pigmentosa, targeting oxidative stress holds potential therapeutic promise. In animal models, reducing light exposure has been shown to diminish photoreceptor degeneration. Antioxidant-rich dietary supplements, e.g., vitamin A, docosahexaenoic acid (DHA), and lutein are commonly used to combat oxidative stress. Some reports suggest a slower decline in ERG amplitudes, indicating a potential slowdown in disease progression [348]. However, systematic reviews of vitamin A and DHA supplementation have not consistently supported their efficacy. Berson et al. [362] noted a decline reduction in high baseline ERG amplitudes with high-dose vitamin A supplementation, although these results were not replicated in larger randomized studies [363]. Other antioxidant agents, including α-tocopherol (vitamin E), Mn(III)tetrakis (4-benzoic acid) porphyrin (a SOD mimetic), ascorbic acid (vitamin C), and α-lipoic acid, have been evaluated in murine models. Studies by Komeima et al. [364] demonstrated reduced oxidized lipid accumulation and cone cell death decline. Lee et al. [365] reported that oral N-acetylcysteine, which restores intracellular GSH, led to reduced rod and cone cell death and potentially displayed neuroprotective effects [366]. Although promising effects of antioxidant supplements have been observed in clinical studies and certain animal models, further comprehensive research is needed to establish broader recommendations.

#### 2.4.5 Retinopathy of prematurity

##### 2.4.5.1 Clinical insights into retinopathy of prematurity

Retinopathy of prematurity (ROP) is a common cause of childhood blindness in premature infants. The Early Treatment for Retinopathy of Prematurity (ET-ROP) study revealed a 68% incidence of any stage of ROP among infants weighing less than 1251 g, with 36.9% progressing to clinically significant ROP [367]. Birthweight and gestational age emerge as key risk factors. The multicenter study of cryotherapy for ROP (CRYO-ROP) demonstrated that a 100 g increase in birthweight corresponded to a 27% decrease in odds for developing threshold ROP, while each additional week of gestational age led to a 19% reduction in risk for threshold ROP development [368]. Premature infants are exposed to a relatively hyperoxic environment when requiring oxygen support. This results in decreased expression of HIFs, erythropoietin (EPO), insulin-like growth factor-1 (IGF-1), and VEGF. Consequently, retinal vessel growth is disrupted, with some formed vessels even regressing, defining the first phase of ROP. Phase 2 involves retinal ischemia, triggering an excessive release of growth factors that fuel abnormal neovascularization. The newly formed vessels grow into the vitreous, culminating in retinal detachment and consecutive vision loss [369]. This transition from phase 1 to phase 2 typically occurs at around 32–34 weeks of postmenstrual age [370]. The two phases are further classified into five stages:

Clinically, stage 1 and 2 are characterized by a demarcation line and ridge between the vascularized and non-vascularized retina, while stage 3 involves extraretinal fibrovascular proliferations. Stage 4 or 5 indicates retinal detachment severity. The retina is divided into three zones from center to periphery [371]. Management and treatment decisions depend on staging and the affected zone. Laser photocoagulation, which is less invasive than ablative cryotherapy, is the primary treatment for avascular retina. Anti-VEGF agents are particularly effective for zone 1 diseases, combined with laser photocoagulation or serving as monotherapy [372]. Gestational age and birth weight, as primary risk factors, often form the basis of ROP screening guidelines [373].

##### 2.4.5.2 Mechanisms of oxidative stress in retinopathy of prematurity

Oxidative stress in infants emerges from the transition between the relatively low oxygen levels of the intrauterine environment and the elevated oxygen pressure in the external environment, especially when supplemental ventilation is required [374]. Retinal oxygen levels become difficult to regulate due to the absence of autoregulatory mechanisms [375,376], resulting in excessive oxygen supply and subsequently increased ROS generation [356]. NO formation is also oxygen-dependent and interacts with ROS to produce nitrites, nitrates, and peroxynitrite, leading to nitro-oxidative stress. These metabolites contribute to retinal microvascular degeneration and apoptosis through nitro-oxidative stress [377,378]. Additionally, the experimental model of oxygen-induced retinopathy demonstrated elevated eNOS expression and activity in oxidative environments, resulting in increased NO production [377]. Nitro-oxidative stress promotes lipid peroxidation in the retina. Given the retinal abundance of polyunsaturated fatty acids like docosahexaenoic acid (DHA), cis-arachidonic acid, and choline phosphoglyceride, susceptibility to such effects is notable. Notably, cis-arachidonic acid can transform into trans-arachidonic acid, provoking vascular damage in murine models. Furthermore, lipid peroxidation elevates levels of platelet activation factor and lysophosphatidic acids, exacerbating microvascular injury and inflammation [379].

Importantly, it is assumed that infants possess reduced antioxidant capacities, further contributing to elevated ROS levels [378]. For instance, Buhimish et al. demonstrated compensatory upregulation of nonenzymatic antioxidant reserves, such as GSH in red blood cell lysate and plasma total free radical-trapping antioxidant potential, in term labor, which was not observed to the same extent in preterm births [380].

##### 2.4.5.3 Therapeutic approaches targeting oxidative stress in retinopathy of prematurity

Oxidative stress, a central component of ROP pathogenesis, is poised to receive greater attention in the future. Promising biomarkers for increased oxidative stress include MDA, 8-OHdG, the GSH/GSSG ratio, isoprostanes, and isofurans [379]. Numerous potential oxidative stress-related treatments are currently under debate, with well-studied or promising options outlined below:

Clinical studies of vitamin E supplementation have yielded conflicting results regarding its impact on ROP incidence and severity [381]. Vitamin C has been discussed as well, functioning as an electron scavenger to reduce free radical reactivity. It also contributes to regeneration of the antioxidant form of vitamin E. While promising results have been demonstrated in cell cultures, clinical studies have not consistently shown significant advantages [379,382]. SOD dismutates the highly reactive superoxide anion and acts as the first line of defense against mitochondrial oxidative stress. In a randomized controlled trial by Parad et al., a 53% reduction in severe ROP was observed for infants born at less than 25 weeks of gestation [383]. Omega-3 long-chain polyunsaturated fatty acids, known for their beneficial effects in various neurodegenerative diseases, inhibited apoptosis through reduction of oxidative stress [384]. A recent randomized clinical trial demonstrated that enteral lipid supplementation (arachidonic acid (AA) and DHA) lowered the risk of severe ROP by 50% for infants born at less than 28 weeks of gestational age [385]. However, the effects of potential antioxidant supplements are debated, and further investigation is clearly warranted.

### 2.5 The optic nerve

The optic nerve is composed of axons from RGCs, which form a superficial layer in the retina termed retinal nerve fiber layer (RNFL) [386]. These ganglion cell extensions converge at the optic disc, also referred to as the optic papilla, giving rise to the optic nerve head, which contains approximately 1.2 million RGC axons [387]. Notably, RGC axons have unmyelinated segments, spanning from the RNFL to the lamina cribrosa, a structure surrounding the optic nerve with around 200–300 porous openings through which the optic nerve exits the eye, acquiring glial coverage and myelination [[387], [388], [389]]. While unmyelinated regions transmit potential signals generated by visual stimuli, they lack the saltatory conduction seen in myelinated fibers due to the absence of myelin [390]. Consequently, these areas require greater energy in the form of ATP to repolarize the cell membrane, thereby enabling the propagation of electrical impulses [391]. In this context, mitochondria play a crucial role. They are primarily responsible for oxygen metabolism, producing energy substrates for intracellular homeostasis, and serving as “dynamic” organelles that move along axons in anterograde or retrograde directions, driven by ATP gradients. This movement is more pronounced in regions with high energy demands, such as the unmyelinated segments [392]. In case of mitochondrial dysfunction, RGCs become susceptible to oxygen deficiency, leading to reduced ATP production and uncurbed generation of ROS and RNS. These factors contribute to optic neuropathy, characterized by RNFL thinning, RGC loss, and optic nerve atrophy [391,393].

RGCs are particularly vulnerable in situations where mitochondrial aerobic respiration is compromised, whether due to genetic mitochondrial disorders, such as Leber’s hereditary optic neuropathy (LHON) or due to inadequate oxygen supply such as in glaucoma or anterior ischemic optic neuropathy (AION).

#### 2.5.1 Glaucoma

##### 2.5.1.1 Clinical insights into glaucoma

Glaucoma constitutes a diverse array of pathologies characterized by the death of RGCs leading to subsequent atrophy of the optic nerve head. This manifests as a reduction of the neuroretinal rim of the optic nerve head, enlargement of the optic nerve head excavation, and deformation of the lamina cribrosa, all indicative of a glaucomatous optic neuropathy [394]. Consequently, optic nerve damage gives rise to distinctive signs, including classic visual field defects that progressively take on an arcuate form [395].

Glaucoma can be classified into a primary form, not linked to co-existing diseases, and a secondary form, contingent upon the presence of pre-existing pathological conditions such as trauma or neovascularization [394]. Furthermore, glaucoma can be categorized as open-angle or angle-closure variants, referring to the iridocorneal angle in the anterior chamber. This angle, which is open in healthy eyes, facilitates aqueous humor (AH) outflow to episcleral veins (trabecular outflow) and to the uvea (uveoscleral outflow) within a normal “AH-turnover” scenario [396]. In contrast to primary open-angle glaucoma, where increase in IOP is detected with an open iridocorneal angle, in primary angle-closure glaucoma, anatomical contact between the iris and trabecular meshwork is seen, leading to closure of the iridocorneal angle, which typically results in highly elevated IOP [396]. Despite accounting for approximately 26% of total glaucoma cases, primary angle-closure glaucoma is particularly devastating and is estimated to contribute to about half of the global glaucoma-related blindness [397,398].

Epidemiologically, glaucoma stands as a leading cause of global visual impairment, with an estimated prevalence of around 3.54% in 2013, typically affecting a population aged 40–80 years, totaling about 64.3 million individuals. By 2040, the glaucoma-affected population is projected to rise to around 111.8 million [399]. A significant risk factor common to both primary open-angle and angle-closure glaucoma pathogenesis is elevated IOP, often defined as a pressure exceeding the 97.5th percentile in the population under consideration, with a typical threshold of >21 mmHg [394]. Elevated IOP plays an essential role in classic pathogenetic hypotheses, which endeavor to elucidate the mechanisms initiating RGC damage. According to this mechanical theory, elevated IOP leads to nerve fiber compression, hindering RGC axoplasmic transport, which culminates in energy substrate depletion and eventual RGC degeneration and cell death [400]. Alternatively, the vascular theory posits optic nerve head atrophy due to insufficient blood supply leading to RGC injury [400].

So far, the primary target of antiglaucomatous therapy is lowering the IOP [[401], [402], [403]].

##### 2.5.1.2 Mechanisms of oxidative stress in glaucoma

ROS play a pivotal role in the pathogenesis of glaucoma, triggering the degeneration and apoptotic or autophagic demise of RGCs, alongside glial activation, aberrant inflammation, optic nerve head remodeling, and subsequent optic nerve atrophy [131]. Multiple studies have reported on elevated oxidative stress markers in serum [404], aqueous humor [405,406], and trabecular meshwork of glaucoma patients [407,408]. Among the frequently studied biomarkers of oxidative damage are 8-OHdG, indicative of DNA damage, and MDA, reflecting lipid peroxidation. A myriad of investigations has demonstrated reduced levels of total antioxidant capacity, total antioxidant status, total reactive antioxidant potential, and biological antioxidant potential, coupled with increased MDA or 8-OHdG levels in blood, aqueous humor, or trabecular meshwork of glaucoma patients. These markers have been correlated with advanced visual field defects, elevated IOP, diminished RGC density, and increased cup-disc ratio (CDR) [[404], [405], [406], [407], [408], [409], [410], [411], [412]].

**Fig. 14:**
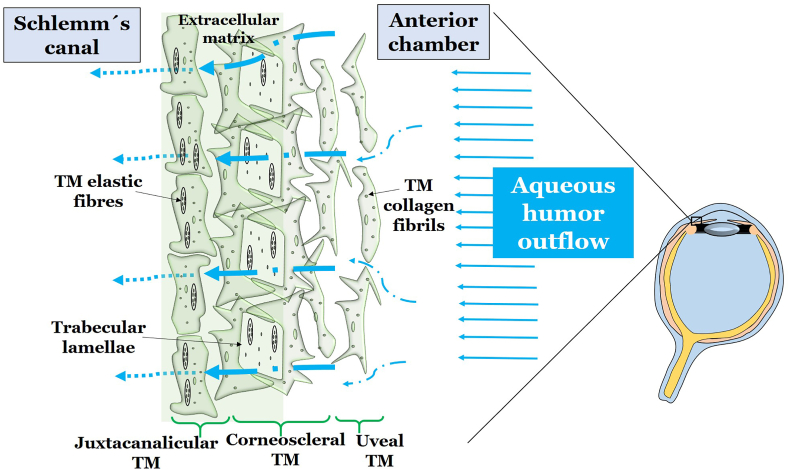
Model representing the trabecular outflow of aqueous humor to the Schlemm’s canal. The aqueous humor first flows through the large intercellular spaces of the uveal trabecular layer. Then, it passes via the intercellular as well as via transcellular route through the trabecular lamellae of the corneoscleral stratum. Subsequently, the aqueous humor reaches the juxtacanalicular trabecular layer, characterized by densely compacted extracellular matrix and reduced intercellular spaces. Finally, through the passage of this last layer, the aqueous humor arrives at the Schlemm’s canal. TM: trabecular meshwork.

Potential sources of ROS in glaucoma are located within various intracellular sites, including mitochondria, ER, and cellular membranes. ROS overproduction extends beyond the retina and optic nerve, affecting trabecular meshwork cells (TMCs), leading to their degeneration and functional impairment [413]. The trabecular meshwork (TM) is situated in the iridocorneal angle and is crucial for IOP regulation. It comprises three anatomical layers: the uveal-TM, corneoscleral-TM, and juxtacanalicular, also known as the cribriform-TM [414]. Among these layers, one notable characteristic is the variable size of intercellular spaces, contingent upon the specific layer under consideration (Fig. 14).

Considering that aqueous humor drainage occurs not only through intercellular pathways but also via transcellular routes, the permeability of the TM emerges as a primary factor in regulating IOP [414,415]. Consequently, any processes inducing alterations in the structure of TMCs may lead to increased resistance for aqueous humor outflow, thereby posing a crucial factor in elevating IOP.

Within this context, oxidative stress emerges as an important contributor to TM damage, particularly in the endothelial TMCs adjoining the Schlemm’s canal [131,413] (Fig. 14). ROS triggers apoptosis of TMCs, disrupts the extracellular matrix of the TM, and fosters TMC fusion and excessive thickness. All these outcomes culminate in increased resistance to aqueous humor outflow, ultimately leading to elevated IOP [413,416,417].

In previous studies involving transgenic mice, we demonstrated that even a moderate elevation in IOP can result in altered vascular autoregulation and endothelial impairment in the retina, coupled with overexpression of NOX2 [418,419]. Elevated IOP has been linked to impaired mitochondrial function, driving mitochondrial fission and impacting the expression of OPA-1 [420]. Furthermore, increased hydrostatic pressure and ischemia prompt the release of TNF-α from glial cells, initiating proapoptotic cascades in RGCs [421]. TNF-α exacerbates mitochondrial dysfunction, oxidative stress, caspase-8-dependent apoptosis and expression of NF-kB, subsequently amplifying neuroinflammation and activating glial cells [421,422]. Moreover, H_2_O_2_-mediated apoptosis plays a critical role by activating apoptosis signal-regulating kinase 1 (ASK-1) [423,424]. This activation leads to the subsequent activation of the p38/protein Janus kinase (pJNK)/protein extracellular signal-regulated kinase (pERK) axis. Consequently, cytochrome *c* is released from the mitochondria to the cytosol, facilitating the formation of the apoptosome, which comprises apoptotic protease-activating factor 1 (Apaf-1), caspase-9, and cytochrome *c*. Ultimately, caspase-3 is activated, responsible for cellular membrane disruption [425,426]. Furthermore, sustained exposure to H_2_O_2_ is believed to activate the phosphoinositide 3-kinase (PI3K)/Akt, also known as protein kinase B (PKB) axis, while concurrently dampening mammalian target of rapamycin (mTor) signaling. This dynamic creates a favorable environment for the activation of the transcription factor NF-kB, thus intensifying neuroinflammation and apoptosis [427].

In cases of normal tension glaucoma, a trigger for glaucomatous pathogenesis may be hypoxia, leading to optic nerve hypoperfusion, even in the absence of elevated IOP. HIF-1α has been identified in the retina and optic nerve of glaucoma patients [428]. This transcription factor instigates the upregulation of angiogenic inflammatory and prooxidative factors, such as VEGF, iNOS and NOX2 [310,[429], [430], [431]]. Interestingly, NOX-generated O_2_^•^ further stimulates HIF-1α expression, thus amplifying its own production [432,433]. Furthermore, also other ROS sources, such as XO and COX were reported in the context of ischemic injuries [434]. These ischemic events, induced by hypoxia, lead to the release of TNF-α by glial cells and the subsequent induction of NF-kB, initiating processes of neuroinflammation, glial activation, and apoptosis through both caspase-dependent [421,422] and caspase-independent pathways [435].

**Fig. 15:**
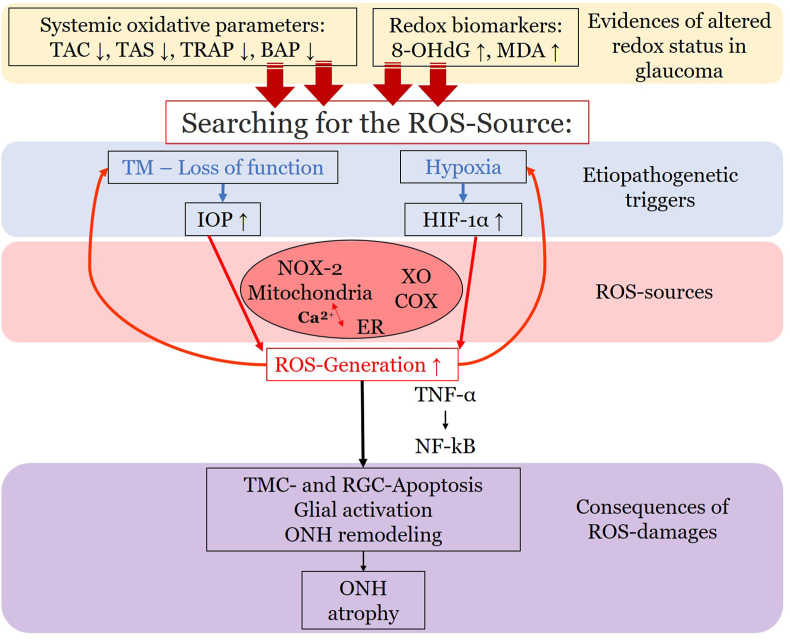
ROS sources and ROS-induced damage in glaucoma. Studies reported decreased systemic antioxidative parameters such as TAC or TAS and elevated redox biomarkers in patients with glaucoma, evidencing the role of the oxidative stress in this disorder. Possible pathophysiological initiators, which drive to an altered redox status, are suggested to be a loss of integrity and function of the trabecular meshwork, with subsequent elevation of IOP, and abnormal perfusion of the retina and optic nerve, causing hypoxia. These conditions trigger ROS-sources, such as NOX2, leading to oxidative stress. The ROS excess in turn exacerbates hypoxia and raised IOP, promoting the glial activation, TMC and RGC death and ONH remodeling. TAS: total antioxidant status; BAP: biological antioxidant potential; TRAP: total reactive antioxidant potential; TAC: total antioxidant capacity; MDA: malonyldialdehyde; 8-OHdG: 8'-hydroxy-2'-deoxyguanosine; ROS: reactive oxygen species; IOP: intraocular pressure; HIF-1α: hypoxia inducible factor 1α; NOX2: nicotinamide adenine dinucleotide phosphate oxidase 2; XO: xanthine oxidase; COX: cyclooxigenase; ER: endoplasmic reticulum; TNF-α: tumor necrosis factor α; NF-kB: nuclear factor ‘kappa-light-chain-enhancer’ of activated B-cells; TMC: trabecular meshwork cell; RGC: retinal ganglion cell; ONH: optic nerve head.

We recently observed that mice lacking the M_1_ muscarinic acetylcholine receptor developed RGC loss at advanced age, which went along with elevated ROS levels and increased mRNA expression for the prooxidative enzyme, NOX2, but decreased mRNA levels for the antioxidant enzymes, SOD1, GPx1 and HO1, suggesting that the M_1_ receptor is involved in modulating redox pathways in the retina [436]. Fig. 15 provides a schematic overview on potential sources of ROS and ROS-mediated effects in glaucoma.

**Fig. 16:**
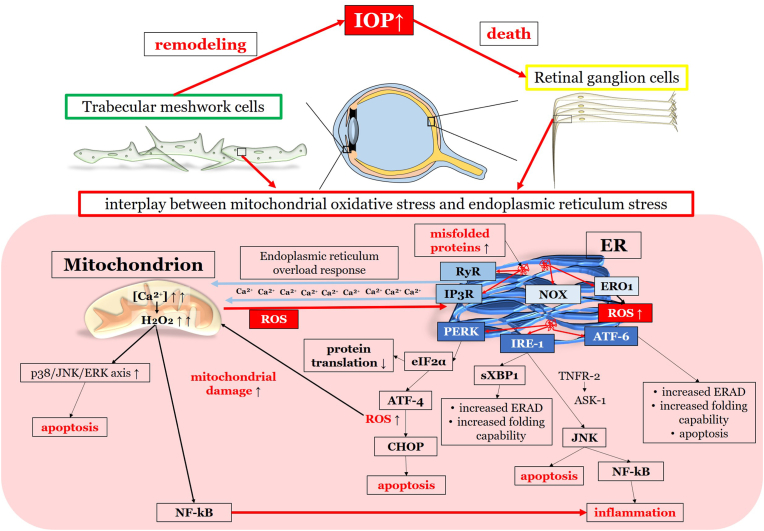
Proposed model of interrelation between mitochondria and endoplasmic reticulum in retinal ganglion cells and trabecular meshwork cells in glaucoma. In the context of glaucoma, TMCs and RGCs establish an interplay between the ER and mitochondrial stress. Under conditions of oxidative stress, the ER becomes oversaturated of misfolded proteins. Consequently, through activation and chronic stimulation of the unfolded protein response, some specific pathways, such as the PERK/ATF-4/CHOP pathway, lead to apoptosis, inflammation, and ROS generation. In addition, calcium ions are released by the stressed ER, and are indirectly gained by the mitochondria, therefore inducing a ROS overproduction and downstream an amplification of inflammation and apoptosis. IOP: intraocular pressure; ER: endoplasmic reticulum; TM: trabecular meshwork; NF-kB: nuclear factor ‘kappa-light-chain-enhancer’ of activated B-cells; ROS: reactive oxygen species; ERAD: ER-associated degradation; eIF2α: eukaryotic initiation factor 2α; PERK: protein kinase RNA-like ER-kinase; IRE-1: inositol-requiring protein 1; ATF: activating transcription factor; CHOP: CCAAT-enhancer-binding protein homologous protein; sXBP1: spliced X-box binding protein-1; TNFR2: tumor necrosis factor receptor 2; ASK-1: apoptosis signal-regulating kinase 1; JNK: Janus kinase; ERK: extracellular-signal-regulated kinase; NOX: nicotinamide adenine dinucleotide phosphate oxidase; ERO1: ER-oxidoreductin 1; RyR: Ryanodine receptor; IP3R: Inositol 1,4,5-trisphosphate receptor.

Another significant factor related to oxidative sources in glaucoma is the role of ER stress. This phenomenon is particularly relevant due to its intricate interplay with mitochondria, playing a role in processes that contribute to TMC degeneration [[437], [438], [439], [440]] and RGC apoptosis [[441], [442], [443], [444]]. Various factors, including hypoxia, oxidative stress, protein mutations, nutrient deficiency, or viral infections, can lead to an impairment of ER functionality reducing its capacity for protein folding, which causes accumulation of misfolded proteins [[445], [446], [447]]. The deposition of misfolded proteins within the ER lumen sets off a series of cascades aimed at restoring intracellular homeostasis (Fig. 16) [446,448]. These processes involve reduced protein synthesis, enhanced protein folding capacity, and the degradation of misfolded proteins through ER-associated degradation (ERAD). ERAD allows the retro-transportation of misfolded proteins from the ER lumen to the cytosol for subsequent removal via the ubiquitin-proteasome system [446,448,449]. However, prolonged activation of this “protein recovery system” can result in cellular damage and apoptosis [446].

Elevated concentrations of misfolded proteins within the ER activate the unfolded protein response, which encompasses three primary pathways: PERK-mediated transduction: This pathway involves protein kinase RNA-like ER kinase (PERK), which activates eukaryotic initiation factor 2α (eIF2α). This leads to reduced protein translation and the activation of the activating transcription factor 4 (ATF-4)/CCAAT-enhancer-binding protein homologous protein (CHOP) pathway. This results in increased ROS production, ATP depletion, and promotion of apoptosis [450].

IRE-1-mediated transduction: In this pathway, inositol-requiring protein 1 (IRE-1) is activated, leading to the stimulation of the tumor necrosis factor receptor 2 (TNRF2)/ASK1/JNK cascade, ultimately inducing apoptosis [447,451]. Additionally, IRE-1 triggers the spliced X-box binding protein-1 (sXBP1), which enhances ERAD and the folding capacity [448]. Activation of transcription factor 6 (ATF-6)–mediated transduction, which stimulates apoptosis, ERAD, and increases the protein folding capability [447,448,452]. Activation of this pathway is closely linked to the unfolded protein response and results in the activation of NOX and endoplasmic reticulum oxidoreductase 1 (ERO-1) on the ER membrane. This leads to an increase in ROS generation associated with ER-related processes [448,453].

Running concurrently with these intricate pathways, the high accumulation of misfolded proteins within the ER lumen prompts an ER overload response, potentially causing the release of Ca^2^⁺ from the ER [446]. This release may be mediated by the ryanodine receptor (RyR) or the inositol 1,4,5-trisphosphate receptor (IP3R) within the cytosol [448]. Consequently, Ca^2^⁺ uptake into the mitochondria is increased [448,454]. Elevated Ca^2^⁺ concentrations within the mitochondria can trigger an increase in H_2_O_2_ production, ultimately leading to mitochondrial dysfunction, excessive ROS generation, and initiation of apoptosis [448,454]. Fig. 16 summarizes the main cascades occurring during the interplay between mitochondrial and ER stress.

In summation, the sources of oxidative stress in glaucoma form an intricate web of interconnections that can be challenging to unravel. The dynamic redox status substantially influences apoptotic processes, where a compromised structural integrity of the trabecular meshwork can culminate in increased IOP and eventual RGC loss. In this intricate dance, the activation of glial cells assumes significance, triggering the release of proinflammatory and proapoptotic cytokines, including TNF-α. Moreover, contemporary research has brought into focus the role of ER stress and the regulation of intracellular calcium levels, shedding light on their impact on mitochondrial-mediated ROS generation, all governed by specific signaling pathways.

##### 2.5.1.3 Therapeutic approaches targeting oxidative stress in glaucoma

Diving into the realm of therapeutic targets for oxidative stress in glaucoma, various molecules with potent antioxidant capabilities have undergone meticulous preclinical scrutiny. These compounds, due to their ability to counteract ROS-induced pathways, hold promise in mitigating the damage sustained by TMCs and RGCs in glaucoma. The principal targets, as illuminated by existing literature, encompass:

1. Key components of ROS-induced proapoptotic cascades including ASK-1, the Bcl-2 associated X-protein (Bax), p53, p38/pJNK/pERK, mTor, Akt, caspase-9, and caspase-3.
2. Antioxidant enzymes such as SOD, GSH, CAT and HO1, whose upregulation augments the capacity to counteract ROS.
3. Nrf2, a transcriptional factor that, when activated, wields a potent dual function of anti-inflammation and antioxidation.
4. NOX, a robust prooxidative enzyme that, when inhibited, indirectly fosters an antioxidant effect.
5. Molecules intricately involved in cascades within the context of ER-stress.

Among these promising candidates, the class of flavonoids shines particularly bright. Having demonstrated success in preclinical assessments, they are now undergoing clinical investigations for glaucoma (NCT01544192; NCT03611530). Derived from plant extracts of Gingko biloba L., a well-known natural medicinal plant, flavonoids encompass more than 70 diverse types [455]. The active compounds from Gingko biloba L. have showcased their potential to curb RGC loss in glaucoma by impeding the action of multiple molecules contributing to ROS-associated apoptosis, including p53, Bax, caspase-9, and caspase-3 [456]. Another beacon of hope in the realm of flavonoids is coenzyme Q10 (CoQ₁₀), a naturally occurring coenzyme. Demonstrating encouraging outcomes in preclinical explorations, CoQ₁₀ exhibits the capacity to quell ROS-mediated proapoptotic cascades. It achieves this feat by diminishing Bax while elevating the presence of the Bcl-2 associated agonist of cell death (Bad) protein in murine models, particularly DBA/2J mice, which serves as a representative model of diffused glaucoma. In addition, CoQ₁₀ effectively counteracts oxidative stress-induced glutamate excitotoxicity [457].

Curcumin, an inherent constituent of turmeric spice with deep-rooted applications in traditional medicine, has been the focus of numerous investigations [458]. Research has unveiled its potential to mitigate apoptosis in microglia exposed to H_2_O_2_ and in murine models with elevated IOP, where it orchestrates a decrease in caspase 3, cytochrome *c*, and Bax, along with an elevation in Bcl-2 levels [459]. Demonstrating its efficacy, curcumin safeguards RGCs from apoptosis in these contexts. Further validation arises from ex vivo assessments, revealing curcumin’s proficiency in curbing RGC loss through the suppression of MAPK, caspase-9, and caspase-3 pathways in murine optic nerves [460].

Resveratrol is a natural ingredient found in berries, peanuts and grapes [461]. The compound has exhibited compelling potential in delaying RGC loss within murine glaucoma models [462]. [463]. Resveratrol shields RGCs from apoptosis caused by ROS, facilitated by enhanced antioxidant activities encompassing SOD, CAT, and GSH. This action involves the suppression of ROS levels and the inhibition of proapoptotic MAPK cascades including p38, pJNK, and pERK [464].

Valproic acid, a prominent antiepileptic medication, has showcased a proclivity for the pERK pathway, effectively reducing RGC loss within mouse normal tension glaucoma models [465]. Its antioxidant efficacy is reflected in ischemic rodent retinal tissues, where it amplifies enzymes such as GPX, SOD, and CAT, thereby quelling ROS-driven apoptotic cascades [466]. Edaravone is a free radical scavenger, utilized in the acute treatment of stroke, which can lead to RGC survival in glaucoma models [467,468] via inhibition of the ROS-induced proapoptotic pJNK/p38 MAPK pathway [469].

Pioneering research has pinpointed the Nrf2 pathway as a pivotal target for prospective antioxidants, owing to its potential to elicit the expression of antioxidant enzymes. Several agents have surfaced as activators of the Nrf2/HO1 pathway, culminating in the suppression of RGC apoptosis. Notable among these is trimetazidine, a drug targeting ischemia [470], astaxanthin, a carotenoid pigment present in microalgae and lobster [471] and α-lipoic acid, a naturally occurring compound in vegetables and fruits. The latter is celebrated for its propensity to upregulate HO1 and NOS in DBA/2J mice, potentially via Nrf2 activation [472].

Turning attention to a cutting-edge avenue, antagonists targeting ER stress emerge as a promising strategy to counteract ER-related ROS production. Numerous studies have reported the involvement of the PERK/eIF2α/ATF4/CHOP cascade in RGC loss [[441], [442], [443], [444]]. Similarly, investigations focusing on TMCs have illuminated the implication of the PERK-eIF2α-ATF4-CHOP transduction in the context of glaucomatous conditions in both human and murine cells [[437], [438], [439], [440]]. Notably, recent research showcased the efficacy of valdecoxib, a selective COX2 inhibitor commonly used to manage osteoarthritis and rheumatoid arthritis, in counteracting apoptosis due to ER stress. This compound effectively suppressed the ATF4/CHOP axis in “I/R-induced glaucoma-like” cells [473].

Further potentially therapeutic compounds targeting oxidative stress are listed in the Online Supplement (Table 2).

#### 2.5.2 Leber’s hereditary optic neuropathy

##### 2.5.2.1 Clinical insights into Leber’s hereditary optic neuropathy

Leber’s hereditary optic neuropathy (LHON) is recognized as the most prevalent inherited mitochondrial disorder. LHON follows a maternal inheritance and predominantly targets male adults aged 35 to 50 [474,475]. The underlying mutations manifest within the subunits of complex I, also referred to as NADH-ubiquinone oxidoreductase or NADH-dehydrogenase, a crucial component of the electron transport chain situated in the inner mitochondrial membrane [475].

LHON’s prevalence in Europe is estimated at approximately 2–4 in 100,000 individuals [[475], [476], [477], [478], [479], [480]]. Notably, LHON displays a pronounced gender bias, affecting males at a higher rate than females [481].

Initial clinical manifestations often start as an acute or subacute event characterized by sudden central vision blurriness. This often commences in one eye and extends to the contralateral eye over a few weeks. Symptomatic bilateral involvement concurrently occurs in only 25% of cases [475,482]. Color perception degradation emerges as an early symptom, with patients typically experiencing no pain, and maintaining intact pupillary reflexes [475]. Perimetry assessments typically unveil classic centrocaecal or central scotomas, accompanied by funduscopic examinations displaying nerve fiber layer swelling, vascular tortuosity, retinal hemorrhages, circumpapillary telangiectatic microangiopathy, and axonal loss within the papillomacular bundle region [475,482,483]. Over a span of 4–6 weeks, visual acuity notably declines. Following this acute phase, peripapillary retinal nerve fiber layer edema regresses initially, but within 6–12 months of symptom onset, visual field defects and acuity loss become more profound. This phase is characterized by the emergence of optic nerve head atrophy, marking the onset of the chronic stage of LHON. Optical coherence tomography (OCT) examinations at this juncture reveal retinal nerve fiber layer thinning [482].

##### 2.5.2.2 Mechanisms of oxidative stress in Leber’s hereditary optic neuropathy

The majority of LHON cases (90–95%) are attributed to one of three primary mutations within complex I: (1) m.3460G>A, affecting the mitochondrial encoded NADH-ubiquinone oxidoreductase chain (MTND) 1 subunit; (2) m.11778G>A, impacting MTND4; and (3) m.14484T>C, influencing MTND6 [484]. These mutations introduce critical structural and functional abnormalities in complex I, a pivotal constituent of the ETC responsible for a substantial portion of ATP synthesis. Despite this dysfunctional complex I and the subsequent reduction in complex I-associated ATP production [614], various tissues can often compensate and stabilize ATP synthesis through alternative energy pathways like glycolysis [485]. In fact, some studies have demonstrated that in individuals with LHON mtDNA mutations exhibit normal energy charge levels or ATP production in lymphocytes and fibroblasts [486,487].

**Fig. 17:**
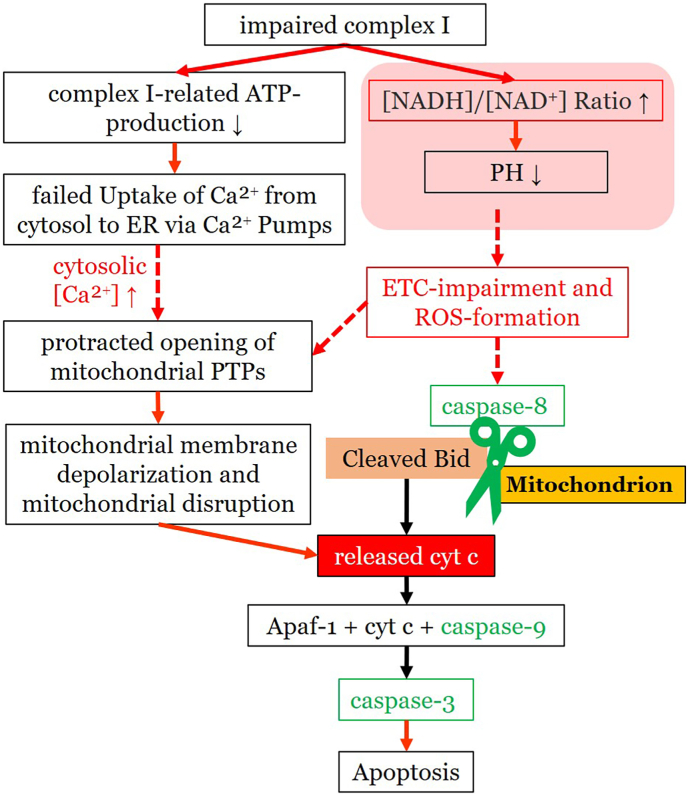
Model representing the LHON pathogenesis and the apoptotic cascade via cytochrome *c*. In LHON, a defected complex I, a main component of the mitochondrial ETC, causes an increased NADH/NAD⁺ ratio and subsequently a decreased PH, driving to an impairment of the oxidative phosphorylation and to ROS overproduction, which ultimately activates caspase-dependent apoptosis. Additionally, the complex I-related ATP production is reduced, therefore driving to a decreased activity of the ATP-dependent calcium pumps of endoplasmic reticulum and consequently to increased cytosolic concentrations of calcium ions. Hereby, a prolonged opening of the mitochondrial PTPs guides to a membrane depolarization and to a mitochondrial disruption, which finally amplifies the cytochrome *c* release and the caspase-dependent apoptosis. ROS: reactive oxygen species; NAD: nicotinamide adenine dinucleotide; NADH: reduced form of nicotinamide adenine dinucleotide; ATP: adenosine triphosphate; Bid: BH3 interacting-domain death agonist; cyt *c*: cytochrome *c*; Apaf-1: apoptotic protease-activating factor 1; PTP: permeability transition pore; LHON: Leber's hereditery optic neuropathy.

Beyond ATP depletion, other mechanisms likely contribute to the pathogenesis of LHON, elucidating its devastating clinical manifestation (Fig. 17). Altered oxidative status within the inner mitochondrial membrane emerges as a potential instigator. An elevated NADH/NAD^+^ ratio diminishes the levels of PH redox sites within the electron transport chain, potentially impairing its functionality and precipitating excessive ROS production [485]. Numerous pieces of evidence underscore an elevated ROS generation in LHON-affected cells, playing a pivotal role in the degeneration of RGCs [485,[488], [489], [490]]. Cells harboring LHON mutations exhibit elevated levels of redox biomarkers such as oxidized GSH and 8-OhdG, coupled with decreased antioxidant activity [[491], [492], [493]].

Moreover, investigations involving LHON mutations in murine models have demonstrated a chronic escalation in ROS generation within synaptosomes, even in the absence of ATP reduction. This highlights the potential dominance of elevated ROS formation over ATP depletion [494]. Remarkably, a single mutation in the ND4 subunit of complex I is adequate to trigger LHON, as elucidated in a study on mice. This cascade of events involves mitochondrial structural disruption, ROS overproduction, activation of ROS-related proapoptotic signaling, and optic nerve head swelling [495,496]. Interestingly, an exploration involving cybrid LHON cells revealed that dysfunctional complex I, marked by elevated ROS levels and augmented cytosolic Ca^2^⁺ concentrations, provokes prolonged opening of permeability transition pores, leading to mitochondrial membrane depolarization [497]. This, in turn, could lead to the release of cytochrome *c* from mitochondria to the cytosol, thereby activating proapoptotic pathways [498]. The potential intracellular Ca^2^⁺ overload could stem from ATP depletion attributed to defective complex I. This impairment might hinder the ability of Ca^2^⁺ pumps on the ER membrane to efficiently transport calcium from the cytosol to the ER [498].

In summary, RGC death via apoptosis may be a consequence of increased concentrations of cytosolic Ca^2^⁺ and of ROS. Apoptotic pathways in LHON were extensively investigated and include a caspase-dependent pathway, via caspase-8/cleaved Bid/cyt *c* and activation of the apoptosome, formed by Apaf-1/cyt *c*/caspase-9, with final induction of caspase-3 [499,500]; as well as caspase independent transductions via apoptosis-inducing factor (AIF) and endonuclease G (EndoG) [501]. However, a release of cytochrome *c* from mitochondria to cytosol represents a common essential event leading to RGC-death. Fig. 17 illustrates the apoptotic transduction occurring in LHON.

##### 2.5.2.3 Therapeutic approaches for oxidative stress in Leber’s hereditary optic neuropathy

The prevailing therapeutic option for LHON centers on idebenone (IDE), which gained approval from the European Medicine Agency in 2015 for young and adult patients at a dose of 300 mg, three times daily. IDE stands as the sole LHON disease-specific medication [502,503]. Structurally categorized as a synthetic hydrosoluble CoQ₁₀ analogue, IDE functions by donating electrons to neutralize free radicals or as an electron carrier within the electron transport chain. It facilitates electron transfer from complex II to complex III, circumventing the defective complex I [[504], [505], [506]].

Minocycline, an antibiotic belonging to the tetracycline class, and exogenous GSH were evaluated for their antioxidative potential on cybrid cells carrying LHON mutations. Both compounds exhibited the capacity to inhibit oxidative stress-mediated proapoptotic pathways, suggesting their utility in counteracting cellular damage [507,508].

EPI-743, also known as α-tocotrienol quinone, stands out as a synthetic drug with potent antioxidative properties. It replenishes glutathione levels by targeting NAD(P)H quinone oxidoreductase 1 (NQO1), thereby enhancing the cellular antioxidant defense mechanisms [502,509]. Clinical trials involving five [510] and twelve patients [511] afflicted with LHON revealed promising outcomes with EPI-743. It effectively halted disease progression and led to improved visual capacities compared to control groups. Nevertheless, more extensive trials assessing the efficacy of EPI-743 for LHON are still pending. Currently, this compound is under investigation in clinical trials for the treatment of primary mitochondrial diseases (NCT01370447; NCT04378075; NCT02352896) [512].

Bendavia, also known as SS-31, MTP-131, or elamipretide, represents a synthetic “mitochondria-targeted peptide” [513] that interacts with cardiolipin, a constituent of the mitochondrial membrane, thereby bolstering mitochondrial membrane stability [509]. This molecule has demonstrated the ability to shield RGCs from oxidative stress-induced apoptosis [513]. Another intriguing synthetic compound is KL-1333, classified as a NAD⁺ modulator. It amplifies the NAD^+^/NADH ratio, leading to decreased lactate and ROS levels, concurrently promoting increased ATP synthesis in fibroblasts affected by mitochondrial encephalopathy lactic acidosis and stroke-like episodes (MELAS) [514].

Collectively, since the clinical approval of IDE, diverse pathways targeting mitochondrial components or molecules capable of improving the redox equilibrium have been explored. These endeavors seek to curtail oxidative stress-induced apoptotic signaling. Novel approaches encompass the reinforcement of antioxidant defense mechanisms, the stabilization of mitochondrial membranes to prevent permeability alterations, and modulation of crucial redox molecules like NAD, culminating in a reduction of oxidative species and an enhancement of energetic substrates. These efforts to develop innovative medications aim to combat the severe onset of this rare yet profoundly debilitating disease.

#### 2.5.3 Anterior ischemic optic neuropathy

##### 2.5.3.1 Clinical insights into anterior ischemic optic neuropathy

Anterior ischemic optic neuropathy (AION) encompasses a spectrum of conditions characterized by ischemic events affecting the optic nerve head, resulting in hypoxic damage and the loss of RGCs. These pathologies are divided into arteritic AION (A-AION), primarily caused by giant cell arteritis, an autoimmune vasculitis, and into non-arteritic AION (NA-AION) [515].

Giant cell arteritis stands as the most prevalent vasculitis in individuals aged over 50 [516], and represents an ocular emergency due to its potential for irreversible visual loss if not promptly diagnosed and treated [517]. Classic symptoms include scalp tenderness, severe headaches in the temporal region, jaw claudication (found in 40% of patients), and unilateral visual loss [518]. Constitutional symptoms like high fever and weight loss may also manifest [518]. Temporal artery tenderness and protuberance are commonly observed during physical examinations [518]. Fundus evaluation reveals optic disc edema, while blood tests detect elevated levels of erythrocyte sedimentation rate (ESR) and C-reactive protein (CRP). Although temporal artery biopsy is considered a diagnostic gold standard, it may yield negative results in 30% of cases [519]. Additional diagnostic tools such as Doppler ultrasound and magnetic resonance imaging aid in identifying stenotic temporal areas, facilitating guided arterial biopsy [519].

Currently, the primary therapeutic approach for giant cell arteritis remains high-dosage intravenous steroid treatment (1000 mg/day for 3 days), followed by gradual dose reduction to 1 mg/kg [520]. An alternative to steroids is tocilizumab, an IL-6 receptor antibody recently approved by the National Institute for Health and Care Excellence and NHS England for refractory giant cell arteritis cases [521].

NA-AION, recognized as the most common form of ischemic optic neuropathy and the second most frequent optic neuropathy after glaucoma, carries an estimated annual incidence of 10.3 per 100,000, typically affecting individuals around the age of 72 [522,523]. Key risk factors for NA-AION include arterial hypertension, coronary heart disease, hyperlipidemia, male gender, sleep apnea, diabetes mellitus, factor V Leiden heterozygosity, and a history of cardiovascular medication use [524]. The pathophysiology involves blockage or vasoconstriction of short posterior ciliary arteries supplying the optic nerve head. Notably, transient nocturnal arterial hypotonia may serve as an initial trigger for NA-AION, leading to optic nerve head hypoperfusion, hypoxia of RGC axons, and subsequent axoplasmic flow stasis [525]. Swollen axons consequently emerge, resulting in clinically detectable optic disc edema. This edema exerts pressure on capillaries supplying the optic nerve head, perpetuating a cycle of optic nerve head hypoperfusion [526]. Clinical presentation includes painless unilateral visual loss accompanied by optic disc edema [527]. Diagnosis involves fundus evaluation for disc edema observation and perimetry for detecting central scotomas, identified in 50% of cases, or the most frequent visual field defect, absolute inferior nasal defect [528]. A noticeable absence of efficacious therapies for NA-AION is evident from the existing literature, urging the exploration of new research avenues, particularly within the antioxidative realm [529].

##### 2.5.3.2 Mechanisms of oxidative stress in anterior ischemic optic neuropathy

The role of altered redox status in the pathogenesis of giant cell arteritis is pronounced due to the prominent risk factor of aging [[530], [531], [532], [533], [534]]. Age-related mitochondrial dysfunction is believed to contribute to an imbalanced redox state, potentially leading to ROS formation in endothelial and vascular smooth muscle cells [530,535,536]. Patients with giant cell arteritis were shown to exhibit elevated systemic oxidative stress indicators, including total antioxidant capacity, oxidative stress biomarkers like MDA, and increased ROS levels in leukocytes [537]. The immune system, particularly neutrophils, is a significant source of ROS generation in giant cell arteritis, with activated neutrophils observed in the vascular lumen and tunica adventitia of temporal arteries in affected individuals [538]. Elevated ROS generation arises from a combination of abnormal age-associated mitochondrial dysfunction and autoimmune-mediated neutrophil activation, fostering significant damage and inflammation within the vascular wall. This cascade culminates in adventitia and intima hyperplasia, resulting in vascular stenosis [530].

Concerning NA-AION, the pivotal pathogenetic factor is hypoxia, which contributes to increased O_2_^•^ generation. Transient hypoperfusion and subsequent ischemic events affecting the optic nerve head are the precursors to hypoxia [526]. Hypoxia induces oxygen generation and ATP depletion, followed by reoxygenation that triggers ROS overproduction, indicative of an ischemia/reperfusion (I/R) injury [[539], [540], [541], [542]]. Our previous investigation in porcine ischemia/reperfusion (I/R) models revealed hypoxia-associated damage, characterized by the overexpression of VEGF, iNOS, HIF-1α, and NOX2, leading to detectable high levels of O_2_^•^ in retinal arterioles, ultimately causing endothelial dysfunction [307]. Studies involving NOX2-deficient mice (NOX2−/−) demonstrated a neuroprotective effect in response to ischemic injuries, favoring mice without NOX2 [310]. Beyond NOX2, other enzymes also play a role in ROS-mediated NA-AION pathogenesis. A loss-of-function mutation in the GSTM1 gene, encoding one of three isoforms of glutathione-S-transferase (GST), an antioxidant enzyme, was linked to NA-AION [543,544]. Additionally, mtDNA mutations were identified in individuals with NA-AION, implying that mitochondrial dysfunction may constitute a potential risk factor [545]. Interestingly, a study involving plasma samples from 18 NA-AION patients found no significant differences in systemic oxidative stress indicators like total antioxidant status and total oxidant status when compared to controls [546]. Reviewing the existing literature, it is reasonable to assert that hypoxia occurring in NA-AION is a primary trigger for ROS formation in this disorder, likely via HIF-1α and NOX2 pathways. Conditions marked by altered redox status, such as mutations in antioxidant enzymes or mitochondrial mutations, should also be considered potential risk factors for the disease.

##### 2.5.3.3 Therapeutic approaches targeting oxidative stress in anterior ischemic optic neuropathy

Giant cell arteritis is characterized by profound inflammatory activations driven by autoimmune mechanisms, making immunomodulation a central focus of research for decades. However, more recent exploration has delved into antioxidant strategies to counteract ROS generation within the highly inflamed blood vessel walls of giant cell arteritis patients. Nuclear sirtuins, particularly SIRT-1, an enzyme regulating gene expression to suppress inflammatory responses and oxidative stress under physiological conditions [547,548], have emerged as potential therapeutic targets for giant cell arteritis. Recent investigations indicate that SIRT-1 expression is downregulated in individuals with giant cell arteritis, suggesting that SIRT-1 activators could offer a pharmacological opportunity for treatment [537].

Numerous antioxidant compounds have been studied in animal models to assess their impact on NA-AION-related oxidative stress (online supplement, Table 3). However, their therapeutic suitability remains to be proven in human trials.

Overall, the lack of effective medications for the relatively prevalent and severe condition of NA-AION emphasizes the need for research into novel pharmacological strategies. Antioxidants offer diverse avenues for targeting oxidative and inflammatory pathways, ultimately suppressing proapoptotic cascades. Promising preclinical outcomes warrant further exploration of antioxidants as potential candidates for dedicated trials.

### 2.6 Future directions

Although antioxidative therapeutic strategies are being developed for years and tested in a myriad of preclinical investigations, the number of molecules that passed to clinical trials is quite small. Idebenone, which is used for the therapy of LHON, has been the first and only specific antioxidant to be approved for disease treatment, so far. It represented a breakthrough in the antioxidant research that opened a wide avenue for new potential antioxidant drugs. The way for a recognized clinical effectiveness of antioxidants in ophthalmology is yet to be fully achieved. An issue is certainly the essential role of ROS in physiological processes, like for example in mediating immune responses. Hence, maintaining a basal level of prooxidant molecules is vital for the homeostasis of the human body. Building on this concept, it is crucial to underline the challenge in designing drugs with antioxidant activity able to contrast the disease and parallelly not to interfere with basal concentrations of oxidative molecules. In fact, this fine balance between oxidant and antioxidant molecules needs to be addressed, to minimize the risk of detrimental side effects. These problematics may be addressed by challenging formulations or balanced dose regimens of antioxidants for human use. In this context, the eye has the advantage of being relatively easily accessible by local roots of application, such as eye drops or intraocular injections.

It is worth noting that in the realm of clinical trials, there exists a notable disparity between the abundance of promising preclinical findings and the relatively limited exploration of novel antioxidant molecules for human use. This discrepancy is underscored by the setbacks encountered in trials that progressed to phase II [[549], [550], [551]]. Delving into this issue, the investigations have provided potential explanations, including challenges in achieving efficient transport to target tissues, limited bioavailability, and even doses that proved too toxic for human use [552]. For example, the gradual and protracted nature of glaucoma progression, combined with the need for extended follow-up periods, places severe constraints on clinical trials, further accentuated by the inadequacy of currently employed biomarkers [549]. Consequently, a surge of recent endeavors has been dedicated to proposing solutions for bridging this gap. Efforts have been channeled towards exploring innovative drug delivery systems [552,553] and devising novel clinical trial designs that enhance adaptability [554,555]. The significance of advancing in these directions cannot be understated, as they hold the potential to significantly mitigate the translational discrepancies currently experienced. By embracing these advancements, the prospect of overcoming hurdles in the translation of promising preclinical outcomes to clinical success may be substantially enhanced.

## 3 Conclusion

In conclusion, an imbalance between ROS generation and impaired antioxidant defense with consequent oxidative stress represents a central aspect in the pathophysiology of various ocular diseases. The ocular surface, including the conjunctiva and the cornea are permanently exposed to UV radiation, which triggers ROS generation. Induction of an imbalance between oxidative and antioxidative mechanisms may promote ocular diseases. During aging and inflammation, instability and hyperosmolarity of the ocular tear film occur, initiating the first step in the vicious circle of DED with further aggravation of inflammation and oxidative stress. In patients with pterygium, reduced antioxidant capacities were observed, and UV radiation as a main risk factor has been shown to cause oxidative DNA damage with consequent induction of apoptosis and uncontrolled cell proliferation.

Studies of corneal diseases, such as keratoconus or FECD also revealed impaired antioxidant capacities and elevated ROS generation inducing apoptosis and loss of keratocytes or endothelial cells leading to further disease progression. Under diabetic conditions, AGEs may aggravate ROS production and activate inflammatory pathways, which consequently impair functions of corneal epithelial cells leading to diabetic keratopathy.

Expression of antioxidant enzymes remarkably declines during aging. Additional metabolic or environmental trigger factors for oxidative stress may accelerate formation of age-related cataract.

The posterior segment of the eye is characterized by high oxygen consumption with elevated generation of ROS. The retina and the optic nerve are very vulnerable to oxidative stress. In case of insufficient antioxidant capacities, retinal and optic nerve diseases can establish. Main risk factors in age-related macular degeneration are aging with a decline of antioxidant functions. UV radiation and blue light, which promote apoptosis and inflammation with consecutive dysfunction of the retinal pigment epithelium, represent main risk factors. Retinal and optic nerve disorders of vascular origin, such as DR, vessel occlusion, retinopathy of prematurity or anterior ischemic optic neuropathies are also characterized by oxidative damage, inflammation and hypoxia, further aggravating ROS generation and disease progression. Moreover, these oxidative processes induce a complex interplay between neuronal and vascular endothelial dysfunction. Elevated IOP is the major risk factor in the pathogenesis of glaucoma. In this context, ROS excess is involved in the vicious circle of neuroinflammation contributing to RGC loss. The trabecular meshwork is also affected by ROS abundance, resulting in impaired IOP regulation.

Various studies aimed to find therapeutic agents to face oxidative stress in ocular diseases. Supplementation of antioxidants, such as vitamin A or E, was discussed in various studies with contradictory results. Idebenone, a synthetic CoQ₁₀ analogue, which gained approval from the European Medicine Agency in 2015, is the only disease-specific medication for LHON. Disturbed function of Nrf2, an important transcription factor in the regulation of antioxidant enzymes, has been reported to play a crucial role in several ocular diseases, and regulation of this pathway represents a promising therapeutic approach. Although, numerous other agents were analyzed in experimental studies in the context of oxidative stress in the eye with promising results, clinical studies are mandatory in this field.

## Authorship

EWB, FB, AV, PB, TS and AG wrote the manuscript. NP reviewed the manuscript.

## Declaration of competing interest

The authors declare that they do not have financial or other conflicts of interest. All authors made substantial contributions to the conception and design of the article, drafting or revising the article for critically important intellectual content and approved the final version of the article.

## Data Availability

No data was used for the research described in the article.

## References

[1] Wang J., Li M., Geng Z., Khattak S., Ji X., Wu D. (2022). Role of oxidative stress in retinal disease and the early intervention strategies: a review. Oxid. Med. Cell. Longev..

[2] Lushchak V.I. (2014). Classification of oxidative stress based on its intensity. Excli j.

[3] Ruan Y., Jiang S., Musayeva A., Gericke A. (2020). Oxidative stress and vascular dysfunction in the retina: therapeutic strategies. Antioxidants (Basel).

[4] Taurone S., Ralli M., Artico M., Madia V.N., Scarpa S., Nottola S.A. (2022). Oxidative stress and visual system: a review. Excli j.

[5] Weng M., Xie X., Liu C., Lim K.L., Zhang C.W., Li L. (2018). The sources of reactive oxygen species and its possible role in the pathogenesis of Parkinson’s disease. Parkinsons Dis..

[6] Brieger K., Schiavone S., Miller F.J., Krause K.H. (2012). Reactive oxygen species: from health to disease. Swiss Med. Wkly..

[7] Dickinson B.C., Chang C.J. (2011). Chemistry and biology of reactive oxygen species in signaling or stress responses. Nat. Chem. Biol..

[8] Manea S.A., Constantin A., Manda G., Sasson S., Manea A. (2015). Regulation of Nox enzymes expression in vascular pathophysiology: focusing on transcription factors and epigenetic mechanisms. Redox Biol..

[9] Förstermann U., Xia N., Li H. (2017). Roles of vascular oxidative stress and nitric oxide in the pathogenesis of atherosclerosis. Circ. Res..

[10] Coon M.J., Vaz A.D., McGinnity D.F., Peng H.M. (1998). Multiple activated oxygen species in P450 catalysis: contributions to specificity in drug metabolism. Drug Metab. Dispos..

[11] Wang Y., Zhang S.X., Gozal D. (2010). Reactive oxygen species and the brain in sleep apnea. Respir. Physiol. Neurobiol..

[12] Dammak A., Pastrana C., Martin-Gil A., Carpena-Torres C., Peral Cerda A., Simovart M. (2023). Oxidative stress in the anterior ocular diseases: diagnostic and treatment. Biomedicines.

[13] Kuntic M., Kuntic I., Krishnankutty R., Gericke A., Oelze M., Junglas T. (2023). Co-exposure to urban particulate matter and aircraft noise adversely impacts the cerebro-pulmonary-cardiovascular axis in mice. Redox Biol..

[14] Hahad O., Kuntic M., Kuntic I., Daiber A., Münzel T. (2023). Tobacco smoking and vascular biology and function: evidence from human studies. Pflügers Archiv.

[15] Guo C., Ning X., Zhang J., Zhang C., Wang J., Su L. (2023). Ultraviolet B radiation induces oxidative stress and apoptosis in human lens epithelium cells by activating NF-κB signaling to down-regulate sodium vitamin C transporter 2 (SVCT2) expression. Cell Cycle.

[16] Bayo Jimenez M.T., Gericke A., Frenis K., Rajlic S., Kvandova M., Kröller-Schön S. (2023). Effects of aircraft noise cessation on blood pressure, cardio- and cerebrovascular endothelial function, oxidative stress, and inflammation in an experimental animal model. Sci. Total Environ..

[17] Lakey P.S., Berkemeier T., Tong H., Arangio A.M., Lucas K., Pöschl U. (2016). Chemical exposure-response relationship between air pollutants and reactive oxygen species in the human respiratory tract. Sci. Rep..

[18] Caliri A.W., Tommasi S., Besaratinia A. (2021). Relationships among smoking, oxidative stress, inflammation, macromolecular damage, and cancer. Mutat. Res. Rev. Mutat. Res..

[19] de Jager T.L., Cockrell A.E., Du Plessis S.S. (2017). Ultraviolet light induced generation of reactive oxygen species. Adv. Exp. Med. Biol..

[20] Marchitti S.A., Chen Y., Thompson D.C., Vasiliou V. (2011). Ultraviolet radiation: cellular antioxidant response and the role of ocular aldehyde dehydrogenase enzymes. Eye Contact Lens.

[21] Hybertson B.M., Gao B., Bose S.K., McCord J.M. (2011). Oxidative stress in health and disease: the therapeutic potential of Nrf2 activation. Mol. Aspect. Med..

[22] Lu J., Holmgren A. (2014). The thioredoxin antioxidant system. Free Radic. Biol. Med..

[23] Franco R., Navarro G., Martínez-Pinilla E. (2019). Antioxidant defense mechanisms in erythrocytes and in the central nervous system. Antioxidants (Basel).

[24] Kirkman H.N., Rolfo M., Ferraris A.M., Gaetani G.F. (1999). Mechanisms of protection of catalase by NADPH. Kinetics and stoichiometry. J. Biol. Chem..

[25] Kirsch M., De Groot H. (2001). NAD(P)H, a directly operating antioxidant?. Faseb. J..

[26] Ho H.Y., Cheng M.L., Chiu D.T. (2007). Glucose-6-phosphate dehydrogenase--from oxidative stress to cellular functions and degenerative diseases. Redox Rep..

[27] Ringvold A., Anderssen E., Kjønniksen I. (1998). Ascorbate in the corneal epithelium of diurnal and nocturnal species. Invest. Ophthalmol. Vis. Sci..

[28] Liebler D.C. (1993). The role of metabolism in the antioxidant function of vitamin E. Crit. Rev. Toxicol..

[29] Glantzounis G.K., Tsimoyiannis E.C., Kappas A.M., Galaris D.A. (2005). Uric acid and oxidative stress. Curr. Pharmaceut. Des..

[30] Palace V.P., Khaper N., Qin Q., Singal P.K. (1999). Antioxidant potentials of vitamin A and carotenoids and their relevance to heart disease. Free Radic. Biol. Med..

[31] Mikhed Y., Daiber A., Steven S. (2015). Mitochondrial oxidative stress, mitochondrial DNA damage and their role in age-related vascular dysfunction. Int. J. Mol. Sci..

[32] Li Y., Zhao T., Li J., Xia M., Li Y., Wang X. (2022). Oxidative stress and 4-hydroxy-2-nonenal (4-HNE): implications in the pathogenesis and treatment of aging-related diseases. J. Immunol. Res..

[33] Di Gioia M., Zanoni I. (2021). Dooming phagocyte responses: inflammatory effects of endogenous oxidized phospholipids. Front. Endocrinol..

[34] Reuter S., Gupta S.C., Chaturvedi M.M., Aggarwal B.B. (2010). Oxidative stress, inflammation, and cancer: how are they linked?. Free Radic. Biol. Med..

[35] Hsueh Y.J., Meir Y.J., Yeh L.K., Wang T.K., Huang C.C., Lu T.T. (2020). Topical ascorbic acid ameliorates oxidative stress-induced corneal endothelial damage via suppression of apoptosis and autophagic flux blockage. Cells.

[36] Hsueh Y.J., Chen Y.N., Tsao Y.T., Cheng C.M., Wu W.C., Chen H.C. (2022). The pathomechanism, antioxidant biomarkers, and treatment of oxidative stress-related eye diseases. Int. J. Mol. Sci..

[37] Mursu J., Robien K., Harnack L.J., Park K., Jacobs D.R. (2011). Dietary supplements and mortality rate in older women: the Iowa Women’s Health Study. Arch. Intern. Med..

[38] Forman H.J., Zhang H. (2021). Targeting oxidative stress in disease: promise and limitations of antioxidant therapy. Nat. Rev. Drug Discov..

[39] Chen Y., Mehta G., Vasiliou V. (2009). Antioxidant defenses in the ocular surface. Ocul. Surf..

[40] Vallabh N.A., Romano V., Willoughby C.E. (2017). Mitochondrial dysfunction and oxidative stress in corneal disease. Mitochondrion.

[41] Böhm E.W., Pfeiffer N., Wagner F.M., Gericke A. (2022). Methods to measure blood flow and vascular reactivity in the retina. Front. Med..

[42] Incalza M.A., D’Oria R., Natalicchio A., Perrini S., Laviola L., Giorgino F. (2018). Oxidative stress and reactive oxygen species in endothelial dysfunction associated with cardiovascular and metabolic diseases. Vasc. Pharmacol..

[43] Sridhar M.S. (2018). Anatomy of cornea and ocular surface. Indian J. Ophthalmol..

[44] Gipson I.K. (2016). Goblet cells of the conjunctiva: a review of recent findings. Prog. Retin. Eye Res..

[45] Ambroziak A.M., Szaflik J., Szaflik J.P., Ambroziak M., Witkiewicz J., Skopiński P. (2016). Immunomodulation on the ocular surface: a review. Cent. Eur. J. Immunol..

[46] Stahl U., Willcox M., Stapleton F. (2012). Osmolality and tear film dynamics. Clin. Exp. Optom..

[47] McCulley J.P., Shine W.E. (2003). Meibomian gland function and the tear lipid layer. Ocul. Surf..

[48] Dilly P.N. (1994). Structure and function of the tear film. Adv. Exp. Med. Biol..

[49] Gogia R., Richer S.P., Rose R.C. (1998). Tear fluid content of electrochemically active components including water soluble antioxidants. Curr. Eye Res..

[50] Behndig A., Svensson B., Marklund S.L., Karlsson K. (1998). Superoxide dismutase isoenzymes in the human eye. Invest. Ophthalmol. Vis. Sci..

[51] O’Neil E.C., Henderson M., Massaro-Giordano M., Bunya V.Y. (2019). Advances in dry eye disease treatment. Curr. Opin. Ophthalmol..

[52] Tsubota K., Pflugfelder S.C., Liu Z., Baudouin C., Kim H.M., Messmer E.M. (2020). Defining dry eye from a clinical perspective. Int. J. Mol. Sci..

[53] Chan T.C.Y., Chow S.S.W., Wan K.H.N., Yuen H.K.L. (2019). Update on the association between dry eye disease and meibomian gland dysfunction. Hong Kong Med. J..

[54] Navel V., Sapin V., Henrioux F., Blanchon L., Labbé A., Chiambaretta F. (2022). Oxidative and antioxidative stress markers in dry eye disease: a systematic review and meta-analysis. Acta Ophthalmol..

[55] Cejková J., Ardan T., Simonová Z., Cejka C., Malec J., Dotrelová D. (2008). Decreased expression of antioxidant enzymes in the conjunctival epithelium of dry eye (Sjögren’s syndrome) and its possible contribution to the development of ocular surface oxidative injuries. Histol. Histopathol..

[56] Lin C.C., Chiu C.C., Lee P.Y., Chen K.J., He C.X., Hsu S.K. (2022). The adverse effects of air pollution on the eye: a review. Int. J. Environ. Res. Publ. Health.

[57] Jung S.J., Mehta J.S., Tong L. (2018). Effects of environment pollution on the ocular surface. Ocul. Surf..

[58] Saccà S.C., Roszkowska A.M., Izzotti A. (2013). Environmental light and endogenous antioxidants as the main determinants of non-cancer ocular diseases. Mutat. Res..

[59] Saccà S.C., Cutolo C.A., Ferrari D., Corazza P., Traverso C.E. (2018). The eye, oxidative damage and polyunsaturated fatty acids. Nutrients.

[60] Zhang H., Davies K.J.A., Forman H.J. (2015). Oxidative stress response and Nrf2 signaling in aging. Free Radic. Biol. Med..

[61] Sohal R.S., Orr W.C. (2012). The redox stress hypothesis of aging. Free Radic. Biol. Med..

[62] Lu C.Y., Lee H.C., Fahn H.J., Wei Y.H. (1999). Oxidative damage elicited by imbalance of free radical scavenging enzymes is associated with large-scale mtDNA deletions in aging human skin. Mutat. Res..

[63] Kojima T., Wakamatsu T.H., Dogru M., Ogawa Y., Igarashi A., Ibrahim O.M. (2012). Age-related dysfunction of the lacrimal gland and oxidative stress: evidence from the Cu,Zn-superoxide dismutase-1 (Sod1) knockout mice. Am. J. Pathol..

[64] Rocha E.M., Alves M., Rios J.D., Dartt D.A. (2008). The aging lacrimal gland: changes in structure and function. Ocul. Surf..

[65] Biswas S.K. (2016). Does the interdependence between oxidative stress and inflammation explain the antioxidant paradox?. Oxid. Med. Cell. Longev..

[66] Ahmad A., Ahsan H. (2020). Biomarkers of inflammation and oxidative stress in ophthalmic disorders. J. Immunoassay Immunochem..

[67] Enríquez-de-Salamanca A., Castellanos E., Stern M.E., Fernández I., Carreño E., García-Vázquez C. (2010). Tear cytokine and chemokine analysis and clinical correlations in evaporative-type dry eye disease. Mol. Vis..

[68] Yoon K.C., Jeong I.Y., Park Y.G., Yang S.Y. (2007). Interleukin-6 and tumor necrosis factor-alpha levels in tears of patients with dry eye syndrome. Cornea.

[69] Enríquez-de-Salamanca A., Calder V., Gao J., Galatowicz G., García-Vázquez C., Fernández I. (2008). Cytokine responses by conjunctival epithelial cells: an in vitro model of ocular inflammation. Cytokine.

[70] Loetscher P., Pellegrino A., Gong J.H., Mattioli I., Loetscher M., Bardi G. (2001). The ligands of CXC chemokine receptor 3, I-TAC, Mig, and IP10, are natural antagonists for CCR3. J. Biol. Chem..

[71] Uchino Y., Kawakita T., Ishii T., Ishii N., Tsubota K. (2012). A new mouse model of dry eye disease: oxidative stress affects functional decline in the lacrimal gland. Cornea.

[72] Baudouin C., Aragona P., Messmer E.M., Tomlinson A., Calonge M., Boboridis K.G. (2013). Role of hyperosmolarity in the pathogenesis and management of dry eye disease: proceedings of the OCEAN group meeting. Ocul. Surf..

[73] Luo L., Li D.Q., Corrales R.M., Pflugfelder S.C. (2005). Hyperosmolar saline is a proinflammatory stress on the mouse ocular surface. Eye Contact Lens.

[74] Deng R., Hua X., Li J., Chi W., Zhang Z., Lu F. (2015). Oxidative stress markers induced by hyperosmolarity in primary human corneal epithelial cells. PLoS One.

[75] Seen S., Tong L. (2018). Dry eye disease and oxidative stress. Acta Ophthalmol..

[76] Ibrahim O.M., Dogru M., Matsumoto Y., Igarashi A., Kojima T., Wakamatsu T.H. (2014). Oxidative stress induced age dependent meibomian gland dysfunction in Cu, Zn-superoxide dismutase-1 (Sod1) knockout mice. PLoS One.

[77] Nezzar H., Mbekeani J.N., Noblanc A., Chiambaretta F., Drevet J.R., Kocer A. (2017). Investigation of antioxidant systems in human meibomian gland and conjunctival tissues. Exp. Eye Res..

[78] Uchino Y., Kawakita T., Miyazawa M., Ishii T., Onouchi H., Yasuda K. (2012). Oxidative stress induced inflammation initiates functional decline of tear production. PLoS One.

[79] Musayeva A., Jiang S., Ruan Y., Zadeh J.K., Chronopoulos P., Pfeiffer N. (2021). Aged mice devoid of the M(3) muscarinic acetylcholine receptor develop mild dry eye disease. Int. J. Mol. Sci..

[80] Batista T.M., Tomiyoshi L.M., Dias A.C., Roma L.P., Módulo C.M., Malki L.T. (2012). Age-dependent changes in rat lacrimal gland anti-oxidant and vesicular related protein expression profiles. Mol. Vis..

[81] Shu D.Y., Chaudhary S., Cho K.S., Lennikov A., Miller W.P., Thorn D.C. (2023). Role of oxidative stress in ocular diseases: a balancing act. Metabolites.

[82] Labetoulle M., Benitez-Del-Castillo J.M., Barabino S., Herrero Vanrell R., Daull P., Garrigue J.S. (2022). Artificial tears: biological role of their ingredients in the management of dry eye disease. Int. J. Mol. Sci..

[83] Regueiro U., López-López M., Varela-Fernández R., Otero-Espinar F.J., Lema I. (2023). Biomedical applications of lactoferrin on the ocular surface. Pharmaceutics.

[84] Versura P., Bavelloni A., Grillini M., Fresina M., Campos E.C. (2013). Diagnostic performance of a tear protein panel in early dry eye. Mol. Vis..

[85] Higuchi A., Inoue H., Kaneko Y., Oonishi E., Tsubota K. (2016). Selenium-binding lactoferrin is taken into corneal epithelial cells by a receptor and prevents corneal damage in dry eye model animals. Sci. Rep..

[86] Pattamatta U., Willcox M., Stapleton F., Garrett Q. (2013). Bovine lactoferrin promotes corneal wound healing and suppresses IL-1 expression in alkali wounded mouse cornea. Curr. Eye Res..

[87] Fujihara T., Nagano T., Nakamura M., Shirasawa E. (1998). Lactoferrin suppresses loss of corneal epithelial integrity in a rabbit short-term dry eye model. J. Ocul. Pharmacol. Therapeut..

[88] Dogru M., Matsumoto Y., Yamamoto Y., Goto E., Saiki M., Shimazaki J. (2007). Lactoferrin in Sjögren’s syndrome. Ophthalmology.

[89] Devendra J., Singh S. (2015). Effect of oral lactoferrin on cataract surgery induced dry eye: a randomised controlled trial. J. Clin. Diagn. Res..

[90] Pastori V., Tavazzi S., Lecchi M. (2015). Lactoferrin-loaded contact lenses: eye protection against oxidative stress. Cornea.

[91] Park Y., Hwang H.B., Kim H.S. (2016). Observation of influence of cataract surgery on the ocular surface. PLoS One.

[92] Brignole-Baudouin F., Riancho L., Liang H., Baudouin C. (2011). Comparative in vitro toxicology study of travoprost polyquad-preserved, travoprost BAK-preserved, and latanoprost BAK-preserved ophthalmic solutions on human conjunctival epithelial cells. Curr. Eye Res..

[93] Jee D., Park M., Lee H.J., Kim M.S., Kim E.C. (2015). Comparison of treatment with preservative-free versus preserved sodium hyaluronate 0.1% and fluorometholone 0.1% eyedrops after cataract surgery in patients with preexisting dry-eye syndrome. J. Cataract Refract. Surg..

[94] Jee D., Park S.H., Kim M.S., Kim E.C. (2014). Antioxidant and inflammatory cytokine in tears of patients with dry eye syndrome treated with preservative-free versus preserved eye drops. Invest. Ophthalmol. Vis. Sci..

[95] Horwath-Winter J., Schmut O., Haller-Schober E.M., Gruber A., Rieger G. (2005). Iodide iontophoresis as a treatment for dry eye syndrome. Br. J. Ophthalmol..

[96] Rieger G., Klieber M., Schimetta W., Pölz W., Griebenow S., Winkler R. (2010). The effect of iodide iontophoresis on the antioxidative capacity of the tear fluid. Graefes Arch. Clin. Exp. Ophthalmol..

[97] Shibata Y., Tanaka Y., Tomita T., Taogoshi T., Kimura Y., Chikama T. (2014). Evaluation of corneal damage caused by iodine preparations using human corneal epithelial cells. Jpn. J. Ophthalmol..

[98] Ghosh A.K., Thapa R., Hariani H.N., Volyanyuk M., Ogle S.D., Orloff K.A. (2021). Poly(lactic-co-glycolic acid) nanoparticles encapsulating the prenylated flavonoid, xanthohumol, protect corneal epithelial cells from dry eye disease-associated oxidative stress. Pharmaceutics.

[99] Chu W.K., Choi H.L., Bhat A.K., Jhanji V. (2020). Pterygium: new insights. Eye (Lond).

[100] Marmamula S., Khanna R.C., Rao G.N. (2013). Population-based assessment of prevalence and risk factors for pterygium in the South Indian state of Andhra Pradesh: the Andhra Pradesh Eye Disease Study. Invest. Ophthalmol. Vis. Sci..

[101] Kilic-Toprak E., Toprak I., Caliskan S., Ozdemir Y., Demirtas O., Altintas F. (2019). Oxidative stress and genotoxicity in pterygium: a systemic investigation. Eye Contact Lens.

[102] Sano I., Kaidzu S., Tanito M., Hara K., Okuno T., Ohira A. (2013). 4-Hydroxyhexenal- and 4-hydroxynonenal-modified proteins in pterygia. Oxid. Med. Cell. Longev..

[103] Elgouhary S.M., Elmazar H.F., Naguib M.I., Bayomy N.R. (2020). Role of oxidative stress and vascular endothelial growth factor expression in pterygium pathogenesis and prevention of pterygium recurrence after surgical excision. Int. Ophthalmol..

[104] Chiang C.C., Cheng Y.W., Lin C.L., Lee H., Tsai F.J., Tseng S.H. (2007). Cyclooxygenase 2 expression in pterygium. Mol. Vis..

[105] Karadag R., Bayram N., Oguztuzun S., Bozer B., Bayramlar H., Simsek G.G. (2016). Investigation of glutathione S-transferase isoenzyme protein expression in patients with pterygium. Cornea.

[106] Wanzeler A.C.V., Barbosa I.A.F., Duarte B., Borges D., Barbosa E.B., Kamiji D. (2019). Mechanisms and biomarker candidates in pterygium development. Arq. Bras. Oftalmol..

[107] Kau H.C., Tsai C.C., Lee C.F., Kao S.C., Hsu W.M., Liu J.H. (2006). Increased oxidative DNA damage, 8-hydroxydeoxy- guanosine, in human pterygium. Eye (Lond).

[108] Kim S.W., Lee J., Lee B., Rhim T. (2014). Proteomic analysis in pterygium; upregulated protein expression of ALDH3A1, PDIA3, and PRDX2. Mol. Vis..

[109] Weinstein O., Rosenthal G., Zirkin H., Monos T., Lifshitz T., Argov S. (2002). Overexpression of p53 tumor suppressor gene in pterygia. Eye (Lond)..

[110] Van Acker S.I., Van den Bogerd B., Haagdorens M., Siozopoulou V., S N.D., Pintelon I. (2021). Pterygium-the good, the bad, and the ugly. Cells.

[111] Di Girolamo N., Coroneo M., Wakefield D. (2005). Epidermal growth factor receptor signaling is partially responsible for the increased matrix metalloproteinase-1 expression in ocular epithelial cells after UVB radiation. Am. J. Pathol..

[112] Dushku N., John M.K., Schultz G.S., Reid T.W. (2001). Pterygia pathogenesis: corneal invasion by matrix metalloproteinase expressing altered limbal epithelial basal cells. Arch. Ophthalmol..

[113] Li D.Q., Lee S.B., Gunja-Smith Z., Liu Y., Solomon A., Meller D. (2001). Overexpression of collagenase (MMP-1) and stromelysin (MMP-3) by pterygium head fibroblasts. Arch. Ophthalmol..

[114] Di Girolamo N., Kumar R.K., Coroneo M.T., Wakefield D. (2002). UVB-mediated induction of interleukin-6 and -8 in pterygia and cultured human pterygium epithelial cells. Invest. Ophthalmol. Vis. Sci..

[115] Martín-López J., Pérez-Rico C., García-Honduvilla N., Buján J., Pascual G. (2019). Elevated blood/lymphatic vessel ratio in pterygium and its relationship with vascular endothelial growth factor (VEGF) distribution. Histol. Histopathol..

[116] Lee D.H., Kim J.K., Joo C.K. (2005). Translocation of nuclear factor-kappaB on corneal epithelial cells induced by ultraviolet B irradiation. Ophthalmic Res..

[117] Morgan M.J., Liu Z.G. (2011). Crosstalk of reactive oxygen species and NF-κB signaling. Cell Res..

[118] Siak J.J., Ng S.L., Seet L.F., Beuerman R.W., Tong L. (2011). The nuclear-factor kappaB pathway is activated in pterygium. Invest. Ophthalmol. Vis. Sci..

[119] Torres J., Enríquez-de-Salamanca A., Fernández I., Rodríguez-Ares M.T., Quadrado M.J., Murta J. (2011). Activation of MAPK signaling pathway and NF-kappaB activation in pterygium and ipsilateral pterygium-free conjunctival specimens. Invest. Ophthalmol. Vis. Sci..

[120] Zaheryani S.M.S., Ebrahimi M.E., Kasaei A., Roointan A., Nejabat M., Dianatpour M. (2018). Expression of inflammatory-related NFκB genes in Iranian patients with pterygium: a case-control study. Int. J. Mol. Cell Med..

[121] Kormanovski A., Parra F., Jarillo-Luna A., Lara-Padilla E., Pacheco-Yépez J., Campos-Rodriguez R. (2014). Oxidant/antioxidant state in tissue of primary and recurrent pterygium. BMC Ophthalmol..

[122] Baheran S.S., Alany R.G., Schwikkard S., Muen W., Salman L.N., Freestone N. (2023). Pharmacological treatment strategies of pterygium: drugs, biologics, and novel natural products. Drug Discov. Today.

[123] Lu C.W., Hao J.L., Yao L., Li H.J., Zhou D.D. (2017). Efficacy of curcumin in inducing apoptosis and inhibiting the expression of VEGF in human pterygium fibroblasts. Int. J. Mol. Med..

[124] Nava-Castañeda A., Olvera-Morales O., Ramos-Castellon C., Garnica-Hayashi L., Garfias Y. (2014). Randomized, controlled trial of conjunctival autografting combined with subconjunctival bevacizumab for primary pterygium treatment: 1-year follow-up. Clin. Exp. Ophthalmol..

[125] Yang H.K., Lee Y.J., Hyon J.Y., Kim K.G., Han S.B. (2020). Efficacy of bevacizumab injection after pterygium excision and limbal conjunctival autograft with limbal fixation suture. Graefes Arch. Clin. Exp. Ophthalmol..

[126] Sayadi J., Gouider D., Henchiri M., Choura R., Boujelbene N., Abbes I. (2022). Preoperative intralesional bevacizumab injection in primary pterygium in Tunisian patients: a randomized controlled prospective study. J. Curr. Ophthalmol..

[127] Kim Y.H., Jung J.C., Jung S.Y., Kim Y.I., Lee K.W., Park Y.J. (2015). Cyclosporine A downregulates MMP-3 and MMP-13 expression in cultured pterygium fibroblasts. Cornea.

[128] Özülken K., Koç M., Ayar O., Hasiripi H. (2012). Topical cyclosporine A administration after pterygium surgery. Eur. J. Ophthalmol..

[129] Yalcin Tok O., Burcu Nurozler A., Ergun G., Akbas Kocaoglu F., Duman S. (2008). Topical cyclosporine A in the prevention of pterygium recurrence. Ophthalmologica.

[130] Shoham A., Hadziahmetovic M., Dunaief J.L., Mydlarski M.B., Schipper H.M. (2008). Oxidative stress in diseases of the human cornea. Free Radical Biol. Med..

[131] Nita M., Grzybowski A. (2016). The role of the reactive oxygen species and oxidative stress in the pathomechanism of the age-related ocular diseases and other pathologies of the anterior and posterior eye segments in adults. Oxid. Med. Cell. Longev..

[132] Araie M., Shirasawa E., Hikita M. (1988). Effect of oxidized glutathione on the barrier function of the corneal endothelium. Invest. Ophthalmol. Vis. Sci..

[133] Suh M.H., Kwon J.W., Wee W.R., Han Y.K., Kim J.H., Lee J.H. (2008). Protective effect of ascorbic Acid against corneal damage by ultraviolet B irradiation: a pilot study. Cornea.

[134] Santodomingo-Rubido J., Carracedo G., Suzaki A., Villa-Collar C., Vincent S.J., Wolffsohn J.S. (2022). Keratoconus: an updated review. Cont Lens Anterior Eye.

[135] Gorskova E.N., Sevost’ianov E.N. (1998). [Epidemiology of keratoconus in the urals]. Vestn. Oftalmol..

[136] Torres Netto E.A., Al-Otaibi W.M., Hafezi N.L., Kling S., Al-Farhan H.M., Randleman J.B. (2018). Prevalence of keratoconus in paediatric patients in Riyadh, Saudi Arabia. Br. J. Ophthalmol..

[137] Hashemi H., Heydarian S., Hooshmand E., Saatchi M., Yekta A., Aghamirsalim M. (2020). The prevalence and risk factors for keratoconus: a systematic review and meta-analysis. Cornea.

[138] Rathi V.M., Mandathara P.S., Dumpati S. (2013). Contact lens in keratoconus. Indian J. Ophthalmol..

[139] Bui A.D., Truong A., Pasricha N.D., Indaram M. (2023). Keratoconus diagnosis and treatment: recent advances and future directions. Clin. Ophthalmol..

[140] Navel V., Malecaze J., Pereira B., Baker J.S., Malecaze F., Sapin V. (2021). Oxidative and antioxidative stress markers in keratoconus: a systematic review and meta-analysis. Acta Ophthalmol..

[141] Toprak I., Kucukatay V., Yildirim C., Kilic-Toprak E., Kilic-Erkek O. (2014). Increased systemic oxidative stress in patients with keratoconus. Eye (Lond)..

[142] Liu R., Yan X. (2021). Oxidative stress in corneal stromal cells contributes to the development of keratoconus in a rabbit model. Eur. J. Ophthalmol..

[143] Cristina Kenney M., Brown D.J. (2003). The cascade hypothesis of keratoconus. Cont Lens Anterior Eye.

[144] Moura G.S., Santos A., Cenedeze M.A., Hiyane M.I., Camara N.O.S., Barbosa de Sousa L. (2021). Increased lacrimal inflammatory mediators in patients with keratoconus. Mol. Vis..

[145] Peyman A., Namgar M., Feizi A., Hakemi M.G., Nasab F.H., Pourazizi M. (2021). Interleukin-6 and tumor necrosis factor-α levels in tear film of Keratoconus patients. J. Res. Med. Sci.: Off. J. Isfahan Univ. Med. Sci..

[146] Sorkhabi R., Ghorbanihaghjo A., Taheri N., Ahoor M.H. (2015). Tear film inflammatory mediators in patients with keratoconus. Int. Ophthalmol..

[147] Chwa M., Atilano S.R., Reddy V., Jordan N., Kim D.W., Kenney M.C. (2006). Increased stress-induced generation of reactive oxygen species and apoptosis in human keratoconus fibroblasts. Invest. Ophthalmol. Vis. Sci..

[148] Karamichos D., Zieske J.D., Sejersen H., Sarker-Nag A., Asara J.M., Hjortdal J. (2015). Tear metabolite changes in keratoconus. Exp. Eye Res..

[149] Chwa M., Atilano S.R., Hertzog D., Zheng H., Langberg J., Kim D.W. (2008). Hypersensitive response to oxidative stress in keratoconus corneal fibroblasts. Invest. Ophthalmol. Vis. Sci..

[150] Kao W.W., Vergnes J.P., Ebert J., Sundar-Raj C.V., Brown S.I. (1982). Increased collagenase and gelatinase activities in keratoconus. Biochem. Biophys. Res. Commun..

[151] McKay T.B., Karamichos D. (2017). Quercetin and the ocular surface: what we know and where we are going. Exp. Biol. Med..

[152] McKay T.B., Sarker-Nag A., Lyon D., Asara J.M., Karamichos D. (2015). Quercetin modulates keratoconus metabolism in vitro. Cell Biochem. Funct..

[153] McKay T.B., Lyon D., Sarker-Nag A., Priyadarsini S., Asara J.M., Karamichos D. (2015). Quercetin attenuates lactate production and extracellular matrix secretion in keratoconus. Sci. Rep..

[154] Pastori V., Tavazzi S., Lecchi M. (2019). Lactoferrin-loaded contact lenses counteract cytotoxicity caused in vitro by keratoconic tears. Cont Lens Anterior Eye.

[155] Lasagni Vitar R.M., Fonteyne P., Knutsson K.A., Bertuzzi F., Galli L., Rama P. (2022). Vitamin D supplementation impacts systemic biomarkers of collagen degradation and copper metabolism in patients with keratoconus. Transl. Vis. Sci. Technol..

[156] Peris-Martínez C., Piá-Ludeña J.V., Rog-Revert M.J., Fernández-López E., Domingo J.C. (2023). Antioxidant and anti-inflammatory effects of oral supplementation with a highly-concentrated docosahexaenoic acid (DHA) triglyceride in patients with keratoconus: a randomized controlled preliminary study. Nutrients.

[157] Liu R., Yan X. (2018). Sulforaphane protects rabbit corneas against oxidative stress injury in keratoconus through activation of the Nrf-2/HO-1 antioxidant pathway. Int. J. Mol. Med..

[158] Joyce N.C., Harris D.L. (2010). Decreasing expression of the G1-phase inhibitors, p21Cip1 and p16INK4a, promotes division of corneal endothelial cells from older donors. Mol. Vis..

[159] Bonanno J.A. (2003). Identity and regulation of ion transport mechanisms in the corneal endothelium. Prog. Retin. Eye Res..

[160] Harris J.E., Nordquist L.T. (1955). The hydration of the cornea. I. The transport of water from the cornea. Am. J. Ophthalmol..

[161] Jurkunas U.V., Bitar M.S., Funaki T., Azizi B. (2010). Evidence of oxidative stress in the pathogenesis of fuchs endothelial corneal dystrophy. Am. J. Pathol..

[162] Wilson S.E., Bourne W.M. (1988). Fuchs' dystrophy. Cornea.

[163] Ong Tone S., Kocaba V., Bohm M., Wylegala A., White T.L., Jurkunas U.V. (2021). Fuchs endothelial corneal dystrophy: the vicious cycle of Fuchs pathogenesis. Prog. Retin. Eye Res..

[164] Gain P., Jullienne R., He Z., Aldossary M., Acquart S., Cognasse F. (2016). Global survey of corneal transplantation and eye banking. JAMA Ophthalmol..

[165] Wong-Riley M.T.T. (2010). Energy metabolism of the visual system. Eye Brain.

[166] Bitar M.S., Liu C., Ziaei A., Chen Y., Schmedt T., Jurkunas U.V. (2012). Decline in DJ-1 and decreased nuclear translocation of Nrf2 in Fuchs endothelial corneal dystrophy. Invest. Ophthalmol. Vis. Sci..

[167] Monteiro de Barros M.R., Chakravarti S. (2022). Pathogenesis of keratoconus: NRF2-antioxidant, extracellular matrix and cellular dysfunctions. Exp. Eye Res..

[168] Zhang J., McGhee C.N.J., Patel D.V. (2019). The molecular basis of fuchs' endothelial corneal dystrophy. Mol. Diagn. Ther..

[169] Halilovic A., Schmedt T., Benischke A.S., Hamill C., Chen Y., Santos J.H. (2016). Menadione-induced DNA damage leads to mitochondrial dysfunction and fragmentation during rosette formation in fuchs endothelial corneal dystrophy. Antioxidants Redox Signal..

[170] Ziaei A., Schmedt T., Chen Y., Jurkunas U.V. (2013). Sulforaphane decreases endothelial cell apoptosis in fuchs endothelial corneal dystrophy: a novel treatment. Invest. Ophthalmol. Vis. Sci..

[171] Kim E.C., Meng H., Jun A.S. (2013). Lithium treatment increases endothelial cell survival and autophagy in a mouse model of Fuchs endothelial corneal dystrophy. Br. J. Ophthalmol..

[172] Kim E.C., Meng H., Jun A.S. (2014). N-Acetylcysteine increases corneal endothelial cell survival in a mouse model of Fuchs endothelial corneal dystrophy. Exp. Eye Res..

[173] Ceravolo I., Mannino F., Irrera N., Minutoli L., Arcoraci V., Altavilla D. (2022). Beneficial effects of polydeoxyribonucleotide (PDRN) in an in vitro model of fuchs endothelial corneal dystrophy. Pharmaceuticals (Basel)..

[174] Wang X., Dong C., Zhou Q., Duan H., Zou D., Gong Y. (2021). Poly(ADP-ribose) polymerase inhibitor PJ34 protects against UVA-induced oxidative damage in corneal endothelium. Apoptosis.

[175] Guo S.P., Chang H.C., Lu L.S., Liu D.Z., Wang T.J. (2021). Activation of kelch-like ECH-associated protein 1/nuclear factor erythroid 2-related factor 2/antioxidant response element pathway by curcumin enhances the anti-oxidative capacity of corneal endothelial cells. Biomed. Pharmacother..

[176] Talpan D., Salla S., Meusel L., Walter P., Kuo C.C., Franzen J. (2023). Cytoprotective effects of human platelet lysate during the xeno-free culture of human donor corneas. Int. J. Mol. Sci..

[177] Priyadarsini S., Whelchel A., Nicholas S., Sharif R., Riaz K., Karamichos D. (2020). Diabetic keratopathy: insights and challenges. Surv. Ophthalmol..

[178] Markoulli M., Flanagan J., Tummanapalli S.S., Wu J., Willcox M. (2018). The impact of diabetes on corneal nerve morphology and ocular surface integrity. Ocul. Surf..

[179] Murphy P.J., Patel S., Kong N., Ryder R.E.J., Marshall J. (2004). Noninvasive assessment of corneal sensitivity in young and elderly diabetic and nondiabetic subjects. Invest. Ophthalmol. Vis. Sci..

[180] Chen W.-L., Lin C.-T., Ko P.-S., Yeh P.-T., Kuan Y.-H., Hu F.-R. (2009). In vivo confocal microscopic findings of corneal wound healing after corneal epithelial debridement in diabetic vitrectomy. Ophthalmology.

[181] Chen H.-F., Yeung L., Yang K.-J., Sun C.-C. (2016). Persistent corneal epithelial defect after pars plana vitrectomy. Retina (Philadelphia, Pa).

[182] Skarbez K., Priestley Y., Hoepf M., Koevary S.B. (2010). Comprehensive review of the effects of diabetes on ocular health. Expet Rev. Ophthalmol..

[183] Karamichos D., Guo X.Q., Hutcheon A.E.K., Zieske J.D. (2010). Human corneal fibrosis: an in vitro model. Invest. Ophthalmol. Vis. Sci..

[184] Kotecha A., Oddone F., Sinapis C., Elsheikh A., Sinapis D., Sinapis A. (2010). Corneal biomechanical characteristics in patients with diabetes mellitus. J. Cataract Refract. Surg..

[185] Schulze S.D., Sekundo W., Kroll P. (2006). Autologous serum for the treatment of corneal epithelial abrasions in diabetic patients undergoing vitrectomy. Am. J. Ophthalmol..

[186] Leong C.Y., Naffi A.A., Wan Abdul Halim W.H., Bastion M.-L.C. (2023). Usage of topical insulin for the treatment of diabetic keratopathy, including corneal epithelial defects. World J. Diabetes.

[187] Shi L., Chen H., Yu X., Wu X. (2013). Advanced glycation end products delay corneal epithelial wound healing through reactive oxygen species generation. Mol. Cell. Biochem..

[188] Suzuki K., Saito J., Yanai R., Yamada N., Chikama T-i, Seki K. (2003). Cell-matrix and cell-cell interactions during corneal epithelial wound healing. Prog. Retin. Eye Res..

[189] Kabosova A., Kramerov A.A., Aoki A.M., Murphy G., Zieske J.D., Ljubimov A.V. (2003). Human diabetic corneas preserve wound healing, basement membrane, integrin and MMP-10 differences from normal corneas in organ culture. Exp. Eye Res..

[190] Wang J., Chen S., Zhao X., Guo Q., Yang R., Zhang C. (2023). Effect of PPARγ on oxidative stress in diabetes-related dry eye. Exp. Eye Res..

[191] Yu F.-S.X., Lee P.S.Y., Yang L., Gao N., Zhang Y., Ljubimov A.V. (2022). The impact of sensory neuropathy and inflammation on epithelial wound healing in diabetic corneas. Prog. Retin. Eye Res..

[192] Mamun A.A., Wu Y., Nasrin F., Akter A., Taniya M.A., Munir F. (2021). Role of pyroptosis in diabetes and its therapeutic implications. J. Inflamm. Res..

[193] Boucek P. (2011). Observing diabetic neuropathy with corneal confocal microscopy: the effect of improvement of risk factors. Expet Rev. Endocrinol. Metabol..

[194] Dang D.H., Riaz K.M., Karamichos D. (2022). Treatment of non-infectious corneal injury: review of diagnostic agents, therapeutic medications, and future targets. Drugs.

[195] Sosne G., Rimmer D., Kleinman H.K., Ousler G. (2016). Thymosin beta 4: a potential novel therapy for neurotrophic keratopathy, dry eye, and ocular surface diseases. Vitam. Horm..

[196] Sosne G., Kleinman H.K. (2015). Primary mechanisms of thymosin β4 repair activity in dry eye disorders and other tissue injuries. Invest. Ophthalmol. Vis. Sci..

[197] Wang Y., Wan L., Zhang Z., Li J., Qu M., Zhou Q. (2021). Topical calcitriol application promotes diabetic corneal wound healing and reinnervation through inhibiting NLRP3 inflammasome activation. Exp. Eye Res..

[198] Liu X., Liu H., Lu X., Zhao S. (2021). N-acetylcysteine alleviates ocular surface damage in STZ-induced diabetic mice by inhibiting the ROS/NLRP3/Caspase-1/IL-1β signaling pathway. Exp. Eye Res..

[199] Abdul-Hamid M., Moustafa N. (2014). Amelioration of alloxan-induced diabetic keratopathy by beta-carotene. Exp. Toxicol. Pathol..

[200] Hamed M.A., Farag A., Zahran I.S., Hafez A., Rizk M.A., Abass M. (2022). Pycnogenol a promising remedy for diabetic keratopathy in experimentally induced corneal alkali burns in diabetic rats. BMC Vet. Res..

[201] Alvarez-Rivera F., Fernández-Villanueva D., Concheiro A., Alvarez-Lorenzo C. (2016). α-Lipoic acid in Soluplus(®) polymeric nanomicelles for ocular treatment of diabetes-associated corneal diseases. J. Pharmaceut. Sci..

[202] Lovicu F.J., McAvoy J.W. (2005). Growth factor regulation of lens development. Dev. Biol..

[203] Foster A., Resnikoff S. (2005). The impact of Vision 2020 on global blindness. Eye (Lond).

[204] Gupta V.B., Rajagopala M., Ravishankar B. (2014). Etiopathogenesis of cataract: an appraisal. Indian J. Ophthalmol..

[205] Lampi K.J., Wilmarth P.A., Murray M.R., David L.L. (2014). Lens β-crystallins: the role of deamidation and related modifications in aging and cataract. Prog. Biophys. Mol. Biol..

[206] Truscott R.J.W., Friedrich M.G. (2019). Molecular processes implicated in human age-related nuclear cataract. Invest. Ophthalmol. Vis. Sci..

[207] Gakamsky A., Duncan R.R., Howarth N.M., Dhillon B., Buttenschon K.K., Daly D.J. (2017). Tryptophan and non-tryptophan fluorescence of the eye lens proteins provides diagnostics of cataract at the molecular level. Sci. Rep..

[208] Modenese A., Gobba F. (2018). Cataract frequency and subtypes involved in workers assessed for their solar radiation exposure: a systematic review. Acta Ophthalmol..

[209] Michael R., Bron A.J. (2011). The ageing lens and cataract: a model of normal and pathological ageing. Phil. Trans. Biol. Sci..

[210] Jahngen-Hodge J., Cyr D., Laxman E., Taylor A. (1992). Ubiquitin and ubiquitin conjugates in human lens. Exp. Eye Res..

[211] Spector A. (1995). Oxidative stress-induced cataract: mechanism of action. Faseb. J.: Off. Pub. Feder. Am. Soc. Exp. Biol..

[212] Giblin F.J. (2000). Glutathione: a vital lens antioxidant. J. Ocul. Pharmacol. Therapeut..

[213] Harding J.J. (2002). Viewing molecular mechanisms of ageing through a lens. Ageing Res. Rev..

[214] Lou M.F., Dickerson J.E. (1992). Protein-thiol mixed disulfides in human lens. Exp. Eye Res..

[215] Takemoto L. (1996). Increase in the intramolecular disulfide bonding of alpha-A crystallin during aging of the human lens. Exp. Eye Res..

[216] Lou M.F. (2003). Redox regulation in the lens. Prog. Retin. Eye Res..

[217] Fan Q., Li D., Zhao Z., Jiang Y., Lu Y. (2022). Protective effect of Glutaredoxin 1 against oxidative stress in lens epithelial cells of age-related nuclear cataracts. Mol. Vis..

[218] Wei M., Xing K.-Y., Fan Y.-C., Libondi T., Lou M.F. (2014). Loss of thiol repair systems in human cataractous lenses. Invest. Ophthalmol. Vis. Sci..

[219] Sweeney M.H.J., Truscott R.J.W. (1998). An impediment to glutathione diffusion in older normal human lenses: a possible precondition for nuclear cataract. Exp. Eye Res..

[220] Hains P.G., Truscott R.J.W. (2007). Post-translational modifications in the nuclear region of young, aged, and cataract human lenses. J. Proteome Res..

[221] Vetter C.J., Thorn D.C., Wheeler S.G., Mundorff C.C., Halverson K.A., Wales T.E. (2020). Cumulative deamidations of the major lens protein gammaS-crystallin increase its aggregation during unfolding and oxidation. Protein Sci..

[222] Norton-Baker B., Mehrabi P., Kwok A.O., Roskamp K.W., Rocha M.A., Sprague-Piercy M.A. (2022). Deamidation of the human eye lens protein gammaS-crystallin accelerates oxidative aging. Structure.

[223] Zhao W.-J., Yan Y.-B. (2018). Increasing susceptibility to oxidative stress by cataract-causing crystallin mutations. Int. J. Biol. Macromol..

[224] Shu D.Y., Ong K., Lovicu F.J. (2017). Histopathology of subcapsular cataract in a patient with atopic dermatitis. Optom. Vis. Sci..

[225] Chamberlain C.G., Mansfield K.J., Cerra A. (2009). Glutathione and catalase suppress TGFbeta-induced cataract-related changes in cultured rat lenses and lens epithelial explants. Mol. Vis..

[226] Shu D.Y., Wojciechowski M., Lovicu F.J. (2019). ERK1/2-mediated EGFR-signaling is required for TGFβ-induced lens epithelial-mesenchymal transition. Exp. Eye Res..

[227] Wang H.J., Zhu J., Zheng G.Y. (2014). Role of glutathione and other antioxidants in the inhibition of apoptosis and mesenchymal transition in rabbit lens epithelial cells. Genet. Mol. Res..

[228] Wang R., Li J., Zhang X., Zhang X., Zhang X., Zhu Y. (2021). Extracellular vesicles promote epithelial-to-mesenchymal transition of lens epithelial cells under oxidative stress. Exp. Cell Res..

[229] Thompson B., Davidson E.A., Chen Y., Orlicky D.J., Thompson D.C., Vasiliou V. (2022). Oxidative stress induces inflammation of lens cells and triggers immune surveillance of ocular tissues. Chem. Biol. Interact..

[230] Braakhuis A.J., Donaldson C.I., Lim J.C., Donaldson P.J. (2019). Nutritional strategies to prevent lens cataract: current status and future strategies. Nutrients.

[231] Tan J.S., Wang J.J., Younan C., Cumming R.G., Rochtchina E., Mitchell P. (2008). Smoking and the long-term incidence of cataract: the blue mountains eye study. Ophthalmic Epidemiol..

[232] Gong Y., Feng K., Yan N., Xu Y., Pan C.W. (2015). Different amounts of alcohol consumption and cataract: a meta-analysis. Optom. Vis. Sci..

[233] Ravindran R.D., Vashist P., Gupta S.K., Young I.S., Maraini G., Camparini M. (2011). Inverse association of vitamin C with cataract in older people in India. Ophthalmology.

[234] Ishikawa Y., Hashizume K., Kishimoto S., Tezuka Y., Nishigori H., Yamamoto N. (2012). Effect of vitamin C depletion on UVR-B induced cataract in SMP30/GNL knockout mice. Exp. Eye Res..

[235] Zhang Y., Jiang W., Xie Z., Wu W., Zhang D. (2015). Vitamin E and risk of age-related cataract: a meta-analysis. Publ. Health Nutr..

[236] Thiagarajan R., Manikandan R. (2013). Antioxidants and cataract. Free Radic. Res..

[237] Jiang H., Yin Y., Wu C.R., Liu Y., Guo F., Li M. (2019). Dietary vitamin and carotenoid intake and risk of age-related cataract. Am. J. Clin. Nutr..

[238] Xu J., Fu Q., Chen X., Yao K. (2020). Advances in pharmacotherapy of cataracts. Ann. Transl. Med..

[239] Mathew M.C., Ervin A.M., Tao J., Davis R.M. (2012). Antioxidant vitamin supplementation for preventing and slowing the progression of age-related cataract. Cochrane Database Syst. Rev..

[240] Rahman S.T., Waterhouse M., Romero B.D., Baxter C., English D., Mackey D.A. (2023). Vitamin D supplementation and the incidence of cataract surgery in older Australian adults. Ophthalmology.

[241] Christen W.G., Glynn R.J., Chew E.Y., Albert C.M., Manson J.E. (2016). Folic acid, vitamin B6, and vitamin B12 in combination and age-related cataract in a randomized trial of women. Ophthalmic Epidemiol..

[242] Christen W.G., Glynn R.J., Sesso H.D., Kurth T., MacFadyen J., Bubes V. (2010). Age-related cataract in a randomized trial of vitamins E and C in men. Arch. Ophthalmol..

[243] Liu X.F., Hao J.L., Xie T., Malik T.H., Lu C.B., Liu C. (2017). Nrf2 as a target for prevention of age-related and diabetic cataracts by against oxidative stress. Aging Cell.

[244] Mi Y., Wei C., Sun L., Liu H., Zhang J., Luo J. (2023). Melatonin inhibits ferroptosis and delays age-related cataract by regulating SIRT6/p-Nrf2/GPX4 and SIRT6/NCOA4/FTH1 pathways. Biomed. Pharmacother..

[245] Yang H., Cui Y., Tang Y., Tang X., Yu X., Zhou J. (2020). Cytoprotective role of humanin in lens epithelial cell oxidative stress-induced injury. Mol. Med. Rep..

[246] Hoon M., Okawa H., Della Santina L., Wong R.O. (2014). Functional architecture of the retina: development and disease. Prog. Retin. Eye Res..

[247] Hurley J.B. (2021). Retina metabolism and metabolism in the pigmented epithelium: a busy intersection. Annu. Rev. Vis. Sci..

[248] Reinhard K., Münch T.A. (2021). Visual properties of human retinal ganglion cells. PLoS One.

[249] Caprara C., Thiersch M., Lange C., Joly S., Samardzija M., Grimm C. (2011). HIF1A is essential for the development of the intermediate plexus of the retinal vasculature. Invest. Ophthalmol. Vis. Sci..

[250] Böhm E.W., Stoffelns B., Gericke A. (2023). β-Adrenoreceptors as therapeutic targets for ocular tumors and other eye diseases-historical aspects and nowadays understanding. Int. J. Mol. Sci..

[251] Markitantova Y., Simirskii V. (2023). Endogenous and exogenous regulation of redox homeostasis in retinal pigment epithelium cells: an updated antioxidant perspective. Int. J. Mol. Sci..

[252] Crabtree M.J., Channon K.M. (2011). Synthesis and recycling of tetrahydrobiopterin in endothelial function and vascular disease. Nitric Oxide.

[253] (2021). Causes of blindness and vision impairment in 2020 and trends over 30 years, and prevalence of avoidable blindness in relation to VISION 2020: the Right to Sight: an analysis for the Global Burden of Disease Study. Lancet Global Health.

[254] Wong W.L., Su X., Li X., Cheung C.M.G., Klein R., Cheng C.-Y. (2014). Global prevalence of age-related macular degeneration and disease burden projection for 2020 and 2040: a systematic review and meta-analysis. Lancet Global Health.

[255] Chakravarthy U., Wong T.Y., Fletcher A., Piault E., Evans C., Zlateva G. (2010). Clinical risk factors for age-related macular degeneration: a systematic review and meta-analysis. BMC Ophthalmol..

[256] Ruan Y., Jiang S., Gericke A. (2021). Age-related macular degeneration: role of oxidative stress and blood vessels. Int. J. Mol. Sci..

[257] Thomas C.J., Mirza R.G., Gill M.K. (2021). Age-related macular degeneration. Med. Clin..

[258] Akyol E., Lotery A. (2020). Gene, cell and antibody-based therapies for the treatment of age-related macular degeneration. Biologics.

[259] Handa J.T. (2012). How does the macula protect itself from oxidative stress?. Mol. Aspect. Med..

[260] Monaghan-Benson E., Hartmann J., Vendrov A.E., Budd S., Byfield G., Parker A. (2010). The role of vascular endothelial growth factor-induced activation of NADPH oxidase in choroidal endothelial cells and choroidal neovascularization. Am. J. Pathol..

[261] Bellezza I. (2018). Oxidative stress in age-related macular degeneration: Nrf2 as therapeutic target. Front. Pharmacol..

[262] Chalam K.V., Khetpal V., Rusovici R., Balaiya S. (2011). A review: role of ultraviolet radiation in age-related macular degeneration. Eye Contact Lens.

[263] Balaiya S., Murthy R.K., Brar V.S., Chalam K.V. (2010). Evaluation of ultraviolet light toxicity on cultured retinal pigment epithelial and retinal ganglion cells. Clin. Ophthalmol..

[264] Glickman R.D. (2011). Ultraviolet phototoxicity to the retina. Eye Contact Lens.

[265] Roehlecke C., Schumann U., Ader M., Brunssen C., Bramke S., Morawietz H. (2013). Stress reaction in outer segments of photoreceptors after blue light irradiation. PLoS One.

[266] Grimm C., Wenzel A., Williams T., Rol P., Hafezi F., Remé C. (2001). Rhodopsin-mediated blue-light damage to the rat retina: effect of photoreversal of bleaching. Invest. Ophthalmol. Vis. Sci..

[267] Mainster M.A., Findl O., Dick H.B., Desmettre T., Ledesma-Gil G., Curcio C.A. (2022). The blue light hazard versus blue light hype. Am. J. Ophthalmol..

[268] Wang L., Cano M., Handa J.T. (2014). p62 provides dual cytoprotection against oxidative stress in the retinal pigment epithelium. Biochim. Biophys. Acta.

[269] Zhao Z., Chen Y., Wang J., Sternberg P., Freeman M.L., Grossniklaus H.E. (2011). Age-related retinopathy in NRF2-deficient mice. PLoS One.

[270] Cano M., Thimmalappula R., Fujihara M., Nagai N., Sporn M., Wang A.L. (2010). Cigarette smoking, oxidative stress, the anti-oxidant response through Nrf2 signaling, and Age-related Macular Degeneration. Vis. Res..

[271] Apte R.S. (2021). Age-related macular degeneration. N. Engl. J. Med..

[272] Panos G.D., Lakshmanan A., Dadoukis P., Ripa M., Motta L., Amoaku W.M. (2023). Faricimab: transforming the future of macular diseases treatment - a comprehensive review of clinical studies. Drug Des. Dev. Ther..

[273] Canonica J., Foxton R., Garrido M.G., Lin C.M., Uhles S., Shanmugam S. (2023). Delineating effects of angiopoietin-2 inhibition on vascular permeability and inflammation in models of retinal neovascularization and ischemia/reperfusion. Front. Cell. Neurosci..

[274] Li L.H., Lee J.C., Leung H.H., Lam W.C., Fu Z., Lo A.C.Y. (2020). Lutein supplementation for eye diseases. Nutrients.

[275] Gao X., Talalay P. (2004). Induction of phase 2 genes by sulforaphane protects retinal pigment epithelial cells against photooxidative damage. Proc. Natl. Acad. Sci. U.S.A..

[276] Tsujinaka H., Itaya-Hironaka A., Yamauchi A., Sakuramoto-Tsuchida S., Makino M.A.I., Shobatake R. (2018). Statins decrease VEGF expression in retinal pigment epithelial cells by downregulation of receptor for AGE (RAGE). Diabetes.

[277] Wagner A.H., Köhler T., Rückschloss U., Just I., Hecker M. (2000). Improvement of nitric oxide-dependent vasodilatation by HMG-CoA reductase inhibitors through attenuation of endothelial superoxide anion formation. Arterioscler. Thromb. Vasc. Biol..

[278] Lin K.Y., Hsih W.H., Lin Y.B., Wen C.Y., Chang T.J. (2021). Update in the epidemiology, risk factors, screening, and treatment of diabetic retinopathy. J. Diabetes Invest..

[279] Saeedi P., Petersohn I., Salpea P., Malanda B., Karuranga S., Unwin N. (2019).

[280] Calderon G.D., Juarez O.H., Hernandez G.E., Punzo S.M., De la Cruz Z.D. (2017). Oxidative stress and diabetic retinopathy: development and treatment. Eye (Lond)..

[281] Dehdashtian E., Mehrzadi S., Yousefi B., Hosseinzadeh A., Reiter R.J., Safa M. (2018). Diabetic retinopathy pathogenesis and the ameliorating effects of melatonin; involvement of autophagy, inflammation and oxidative stress. Life Sci..

[282] Wilkinson C.P., Ferris F.L., Klein R.E., Lee P.P., Agardh C.D., Davis M. (2003). Proposed international clinical diabetic retinopathy and diabetic macular edema disease severity scales. Ophthalmology.

[283] Arabi A., Tadayoni R., Ahmadieh H., Shahraki T., Nikkhah H. (2022). Update on management of non-proliferative diabetic retinopathy without diabetic macular edema; is there a paradigm shift?. J. Ophthalmic Vis. Res..

[284] Wu M.Y., Yiang G.T., Lai T.T., Li C.J. (2018). The oxidative stress and mitochondrial dysfunction during the pathogenesis of diabetic retinopathy. Oxid. Med. Cell. Longev..

[285] Lorenzi M. (2007). The polyol pathway as a mechanism for diabetic retinopathy: attractive, elusive, and resilient. Exp. Diabetes Res..

[286] Rodríguez M.L., Pérez S., Mena-Mollá S., Desco M.C., Ortega Á L. (2019). Oxidative stress and microvascular alterations in diabetic retinopathy: future therapies. Oxid. Med. Cell. Longev..

[287] Yamagishi S., Matsui T. (2011). Advanced glycation end products (AGEs), oxidative stress and diabetic retinopathy. Curr. Pharmaceut. Biotechnol..

[288] Kang Q., Yang C. (2020). Oxidative stress and diabetic retinopathy: molecular mechanisms, pathogenetic role and therapeutic implications. Redox Biol..

[289] Andrade A.S., Salomon T.B., Behling C.S., Mahl C.D., Hackenhaar F.S., Putti J. (2014). Alpha-lipoic acid restores tear production in an animal model of dry eye. Exp. Eye Res..

[290] Kumari N., Karmakar A., Ganesan S.K. (2020). Targeting epigenetic modifications as a potential therapeutic option for diabetic retinopathy. J. Cell. Physiol..

[291] Perrone L., Matrone C., Singh L.P. (2014). Epigenetic modifications and potential new treatment targets in diabetic retinopathy. J. Ophthalmol..

[292] Mishra M., Zhong Q., Kowluru R.A. (2014). Epigenetic modifications of Keap1 regulate its interaction with the protective factor Nrf2 in the development of diabetic retinopathy. Invest. Ophthalmol. Vis. Sci..

[293] Sun J., Xu Y., Sun S., Sun Y., Wang X. (2010). Intermittent high glucose enhances cell proliferation and VEGF expression in retinal endothelial cells: the role of mitochondrial reactive oxygen species. Mol. Cell. Biochem..

[294] Bradley M.A., Xiong-Fister S., Markesbery W.R., Lovell M.A. (2012). Elevated 4-hydroxyhexenal in Alzheimer’s disease (AD) progression. Neurobiol. Aging.

[295] Sharma A., Sharma R., Chaudhary P., Vatsyayan R., Pearce V., Jeyabal P.V. (2008). 4-Hydroxynonenal induces p53-mediated apoptosis in retinal pigment epithelial cells. Arch. Biochem. Biophys..

[296] Kowluru R.A., Koppolu P. (2002). Diabetes-induced activation of caspase-3 in retina: effect of antioxidant therapy. Free Radic. Res..

[297] Pearsall E.A., Cheng R., Matsuzaki S., Zhou K., Ding L., Ahn B. (2019). Neuroprotective effects of PPARα in retinopathy of type 1 diabetes. PLoS One.

[298] Hass D.T., Barnstable C.J. (2021). Uncoupling proteins in the mitochondrial defense against oxidative stress. Prog. Retin. Eye Res..

[299] Payne A., Nahashon S., Taka E., Adinew G.M., Soliman K.F.A. (2022). Epigallocatechin-3-Gallate (EGCG): new therapeutic perspectives for neuroprotection, aging, and neuroinflammation for the modern age. Biomolecules.

[300] Meng J.M., Cao S.Y., Wei X.L., Gan R.Y., Wang Y.F., Cai S.X. (2019). Effects and mechanisms of tea for the prevention and management of diabetes mellitus and diabetic complications: an updated review. Antioxidants (Basel).

[301] Dauth A., Breborowicz A., Ruan Y., Tang Q., Zadeh J.K., Bohm E.W. (2023). Sulodexide prevents hyperglycemia-induced endothelial dysfunction and oxidative stress in porcine retinal arterioles. Antioxidants.

[302] Hayreh S.S. (2018). Central retinal artery occlusion. Indian J. Ophthalmol..

[303] Varma D.D., Cugati S., Lee A.W., Chen C.S. (2013). A review of central retinal artery occlusion: clinical presentation and management. Eye (Lond)..

[304] Feltgen N., Neubauer A., Jurklies B., Schmoor C., Schmidt D., Wanke J. (2006). Multicenter study of the European Assessment Group for Lysis in the Eye (EAGLE) for the treatment of central retinal artery occlusion: design issues and implications. EAGLE Study report no. 1 : EAGLE Study report no. 1. Graefes Arch. Clin. Exp. Ophthalmol..

[305] Nicholson L., Talks S.J., Amoaku W., Talks K., Sivaprasad S. (2022). Retinal vein occlusion (RVO) guideline: executive summary. Eye (Lond)..

[306] Jonas J.B., Monés J., Glacet-Bernard A., Coscas G. (2017). Retinal vein occlusions. Dev. Ophthalmol..

[307] Zadeh J.K., Garcia-Bardon A., Hartmann E.K., Pfeiffer N., Omran W., Ludwig M. (2019). Short-time ocular ischemia induces vascular endothelial dysfunction and ganglion cell loss in the pig retina. Int. J. Mol. Sci..

[308] Musayeva A., Unkrig J.C., Zhutdieva M.B., Manicam C., Ruan Y., Laspas P. (2021). Betulinic acid protects from ischemia-reperfusion injury in the mouse retina. Cells.

[309] Chronopoulos P., Manicam C., Zadeh J.K., Laspas P., Unkrig J.C., Göbel M.L. (2023). Effects of resveratrol on vascular function in retinal ischemia-reperfusion injury. Antioxidants (Basel).

[310] Yokota H., Narayanan S.P., Zhang W., Liu H., Rojas M., Xu Z. (2011). Neuroprotection from retinal ischemia/reperfusion injury by NOX2 NADPH oxidase deletion. Invest. Ophthalmol. Vis. Sci..

[311] Prasad S.S., Kojic L., Wen Y.H., Chen Z., Xiong W., Jia W. (2010). Retinal gene expression after central retinal artery ligation: effects of ischemia and reperfusion. Invest. Ophthalmol. Vis. Sci..

[312] Joo C.K., Choi J.S., Ko H.W., Park K.Y., Sohn S., Chun M.H. (1999). Necrosis and apoptosis after retinal ischemia: involvement of NMDA-mediated excitotoxicity and p53. Invest. Ophthalmol. Vis. Sci..

[313] Lam T.T., Abler A.S., Tso M.O. (1999). Apoptosis and caspases after ischemia-reperfusion injury in rat retina. Invest. Ophthalmol. Vis. Sci..

[314] Xu Z., Cho H., Hartsock M.J., Mitchell K.L., Gong J., Wu L. (2015). Neuroprotective role of Nrf2 for retinal ganglion cells in ischemia-reperfusion. J. Neurochem..

[315] Ulbrich F., Lerach T., Biermann J., Kaufmann K.B., Lagreze W.A., Buerkle H. (2016). Argon mediates protection by interleukin-8 suppression via a TLR2/TLR4/STAT3/NF-κB pathway in a model of apoptosis in neuroblastoma cells in vitro and following ischemia-reperfusion injury in rat retina in vivo. J. Neurochem..

[316] Chen H., Song Z., Ying S., Yang X., Wu W., Tan Q. (2018). Myeloid differentiation protein 2 induced retinal ischemia reperfusion injury via upregulation of ROS through a TLR4-NOX4 pathway. Toxicol. Lett..

[317] Chen K.H., Hsiang E.L., Hsu M.Y., Chou Y.C., Lin T.C., Chang Y.L. (2019). Elevation of serum oxidative stress in patients with retina vein occlusions. Acta Ophthalmol..

[318] Altinisik M., Koytak A., Elbay A., Toklu E., Sezer T., Kocyigit A. (2018). Oxidant-antioxidant balance in the aqueous humor of patients with retinal vein occlusion. Semin. Ophthalmol..

[319] Becatti M., Marcucci R., Gori A.M., Mannini L., Grifoni E., Alessandrello Liotta A. (2016). Erythrocyte oxidative stress is associated with cell deformability in patients with retinal vein occlusion. J. Thromb. Haemostasis.

[320] Bharathi Devi S.R., Suganeswari G., Sharma T., Thennarasu M., Angayarkanni N. (2012). Homocysteine induces oxidative stress in young adult central retinal vein occlusion. Br. J. Ophthalmol..

[321] Noma H., Funatsu H., Mimura T., Harino S., Hori S. (2009). Vitreous levels of interleukin-6 and vascular endothelial growth factor in macular edema with central retinal vein occlusion. Ophthalmology.

[322] Suzuki Y., Nakazawa M., Suzuki K., Yamazaki H., Miyagawa Y. (2011). Expression profiles of cytokines and chemokines in vitreous fluid in diabetic retinopathy and central retinal vein occlusion. Jpn. J. Ophthalmol..

[323] Yu H., Huang X., Ma Y., Gao M., Wang O., Gao T. (2013). Interleukin-8 regulates endothelial permeability by down-regulation of tight junction but not dependent on integrins induced focal adhesions. Int. J. Biol. Sci..

[324] Noma H., Yasuda K., Shimura M. (2020). Cytokines and pathogenesis of central retinal vein occlusion. J. Clin. Med..

[325] Zhang X.Y., Xiao Y.Q., Zhang Y., Ye W. (2013). Protective effect of pioglitazone on retinal ischemia/reperfusion injury in rats. Invest. Ophthalmol. Vis. Sci..

[326] Fang I.M., Yang C.M., Yang C.H. (2015). Chitosan oligosaccharides prevented retinal ischemia and reperfusion injury via reduced oxidative stress and inflammation in rats. Exp. Eye Res..

[327] Wang J., Sun Z., Shen J., Wu D., Liu F., Yang R. (2015). Octreotide protects the mouse retina against ischemic reperfusion injury through regulation of antioxidation and activation of NF-κB. Oxid. Med. Cell. Longev..

[328] Qin Q., Yu N., Gu Y., Ke W., Zhang Q., Liu X. (2022). Inhibiting multiple forms of cell death optimizes ganglion cells survival after retinal ischemia reperfusion injury. Cell Death Dis..

[329] Ozaki T., Yamashita T., Tomita H., Sugano E., Ishiguro S. (2016). The protection of rat retinal ganglion cells from ischemia/reperfusion injury by the inhibitory peptide of mitochondrial μ-calpain. Biochem. Biophys. Res. Commun..

[330] Kim B.J., Silverman S.M., Liu Y., Wordinger R.J., Pang I.H., Clark A.F. (2016). In vitro and in vivo neuroprotective effects of cJun N-terminal kinase inhibitors on retinal ganglion cells. Mol. Neurodegener..

[331] Shima C., Adachi Y., Minamino K., Okigaki M., Shi M., Imai Y. (2012). Neuroprotective effects of granulocyte colony-stimulating factor on ischemia-reperfusion injury of the retina. Ophthalmic Res..

[332] Lee D., Kim K.Y., Shim M.S., Kim S.Y., Ellisman M.H., Weinreb R.N. (2014). Coenzyme Q10 ameliorates oxidative stress and prevents mitochondrial alteration in ischemic retinal injury. Apoptosis.

[333] Lee D., Kim K.Y., Noh Y.H., Chai S., Lindsey J.D., Ellisman M.H. (2012). Brimonidine blocks glutamate excitotoxicity-induced oxidative stress and preserves mitochondrial transcription factor a in ischemic retinal injury. PLoS One.

[334] Hui Q., Karlstetter M., Xu Z., Yang J., Zhou L., Eilken H.M. (2020). Inhibition of the Keap1-Nrf2 protein-protein interaction protects retinal cells and ameliorates retinal ischemia-reperfusion injury. Free Radic. Biol. Med..

[335] Cho H., Hartsock M.J., Xu Z., He M., Duh E.J. (2015). Monomethyl fumarate promotes Nrf2-dependent neuroprotection in retinal ischemia-reperfusion. J. Neuroinflammation.

[336] Xia J.P., Wang S., Zhang J.S. (2019). The anti-inflammatory and anti-oxidative effects of conbercept in treatment of macular edema secondary to retinal vein occlusion. Biochem. Biophys. Res. Commun..

[337] Neo T., Gozawa M., Takamura Y., Inatani M., Oki M. (2020). Gene expression profile analysis of the rabbit retinal vein occlusion model. PLoS One.

[338] Ambwani S., Dolma R., Sharma R., Kaur A., Singh H., Ruj A. (2023). Modulation of inflammatory and oxidative stress biomarkers due to dexamethasone exposure in chicken splenocytes. Vet. Immunol. Immunopathol..

[339] Adachi T., Teramachi M., Yasuda H., Kamiya T., Hara H. (2012). Contribution of p38 MAPK, NF-κB and glucocorticoid signaling pathways to ER stress-induced increase in retinal endothelial permeability. Arch. Biochem. Biophys..

[340] Pagon R.A. (1988). Retinitis pigmentosa. Surv. Ophthalmol..

[341] Daiger S.P., Sullivan L.S., Bowne S.J. (2013). Genes and mutations causing retinitis pigmentosa. Clin. Genet..

[342] Liu W., Liu S., Li P., Yao K. (2022). Retinitis pigmentosa: progress in molecular pathology and biotherapeutical strategies. Int. J. Mol. Sci..

[343] Hamel C. (2006). Retinitis pigmentosa. Orphanet J. Rare Dis..

[344] Colombo L., Maltese P.E., Castori M., El Shamieh S., Zeitz C., Audo I. (2021). Molecular epidemiology in 591 Italian probands with nonsyndromic retinitis pigmentosa and usher syndrome. Invest. Ophthalmol. Vis. Sci..

[345] Fujiwara K., Ikeda Y., Murakami Y., Nakatake S., Tachibana T., Yoshida N. (2016). Association between aqueous flare and epiretinal membrane in retinitis pigmentosa. Invest. Ophthalmol. Vis. Sci..

[346] Strong S., Liew G., Michaelides M. (2017). Retinitis pigmentosa-associated cystoid macular oedema: pathogenesis and avenues of intervention. Br. J. Ophthalmol..

[347] Verbakel S.K., van Huet R.A.C., Boon C.J.F., den Hollander A.I., Collin R.W.J., Klaver C.C.W. (2018). Non-syndromic retinitis pigmentosa. Prog. Retin. Eye Res..

[348] Wu K.Y., Kulbay M., Toameh D., Xu A.Q., Kalevar A., Tran S.D. (2023). Retinitis pigmentosa: novel therapeutic targets and drug development. Pharmaceutics.

[349] Murakami Y., Nakabeppu Y., Sonoda K.H. (2020). Oxidative stress and microglial response in retinitis pigmentosa. Int. J. Mol. Sci..

[350] Murakami Y., Ikeda Y., Yoshida N., Notomi S., Hisatomi T., Oka S. (2012). MutT homolog-1 attenuates oxidative DNA damage and delays photoreceptor cell death in inherited retinal degeneration. Am. J. Pathol..

[351] Martínez-Fernández de la Cámara C., Salom D., Sequedo M.D., Hervás D., Marín-Lambíes C., Aller E. (2013). Altered antioxidant-oxidant status in the aqueous humor and peripheral blood of patients with retinitis pigmentosa. PLoS One.

[352] Campochiaro P.A., Mir T.A. (2018). The mechanism of cone cell death in Retinitis Pigmentosa. Prog. Retin. Eye Res..

[353] Yu D.Y., Cringle S., Valter K., Walsh N., Lee D., Stone J. (2004). Photoreceptor death, trophic factor expression, retinal oxygen status, and photoreceptor function in the P23H rat. Invest. Ophthalmol. Vis. Sci..

[354] Usui S., Oveson B.C., Iwase T., Lu L., Lee S.Y., Jo Y.-J. (2011). Overexpression of SOD in retina: need for increase in H2O2-detoxifying enzyme in same cellular compartment. Free Radic. Biol. Med..

[355] Usui S., Oveson B.C., Lee S.Y., Jo Y.-J., Yoshida T., Miki A. (2009). NADPH oxidase plays a central role in cone cell death in retinitis pigmentosa. J. Neurochem..

[356] Tarafdar A., Pula G. (2018). The role of NADPH oxidases and oxidative stress in neurodegenerative disorders. Int. J. Mol. Sci..

[357] Gallenga C.E., Lonardi M., Pacetti S., Violanti S.S., Tassinari P., Di Virgilio F. (2021). Molecular mechanisms related to oxidative stress in retinitis pigmentosa. Antioxidants (Basel).

[358] Tuson M., Garanto A., Gonzàlez-Duarte R., Marfany G. (2009). Overexpression of CERKL, a gene responsible for retinitis pigmentosa in humans, protects cells from apoptosis induced by oxidative stress. Mol. Vis..

[359] Gorbatyuk M.S., Starr C.R., Gorbatyuk O.S. (2020). Endoplasmic reticulum stress: new insights into the pathogenesis and treatment of retinal degenerative diseases. Prog. Retin. Eye Res..

[360] Datta S., Cano M., Ebrahimi K., Wang L., Handa J.T. (2017). The impact of oxidative stress and inflammation on RPE degeneration in non-neovascular AMD. Prog. Retin. Eye Res..

[361] BD E., Marfany G. (2020). The relevance of oxidative stress in the pathogenesis and therapy of retinal dystrophies. Antioxidants (Basel).

[362] Berson E.L., Rosner B., Sandberg M.A., Hayes K.C., Nicholson B.W., Weigel-DiFranco C. (1993). A randomized trial of vitamin A and vitamin E supplementation for retinitis pigmentosa. Arch. Ophthalmol..

[363] Rayapudi S., Schwartz S.G., Wang X., Chavis P. (2013). Vitamin A and fish oils for retinitis pigmentosa. Cochrane Database Syst. Rev..

[364] Komeima K., Rogers B.S., Lu L., Campochiaro P.A. (2006). Antioxidants reduce cone cell death in a model of retinitis pigmentosa. Proc. Natl. Acad. Sci. U.S.A..

[365] Lee S.Y., Usui S., Zafar A.B., Oveson B.C., Jo Y.J., Lu L. (2011). N-Acetylcysteine promotes long-term survival of cones in a model of retinitis pigmentosa. J. Cell. Physiol..

[366] Yoshida N., Ikeda Y., Notomi S., Ishikawa K., Murakami Y., Hisatomi T. (2013). Laboratory evidence of sustained chronic inflammatory reaction in retinitis pigmentosa. Ophthalmology.

[367] Good W.V., Hardy R.J., Dobson V., Palmer E.A., Phelps D.L., Quintos M. (2005). The incidence and course of retinopathy of prematurity: findings from the early treatment for retinopathy of prematurity study. Pediatrics.

[368] Schaffer D.B., Palmer E.A., Plotsky D.F., Metz H.S., Flynn J.T., Tung B. (1993). Prognostic factors in the natural course of retinopathy of prematurity. The cryotherapy for retinopathy of prematurity cooperative group. Ophthalmology.

[369] Fevereiro-Martins M., Marques-Neves C., Guimarães H., Bicho M. (2023). Retinopathy of prematurity: a review of pathophysiology and signaling pathways. Surv. Ophthalmol..

[370] Palmer E.A., Flynn J.T., Hardy R.J., Phelps D.L., Phillips C.L., Schaffer D.B. (2020). Incidence and early course of retinopathy of prematurity. Ophthalmology.

[371] Hellström A., Smith L.E., Dammann O. (2013). Retinopathy of prematurity. Lancet.

[372] Bancalari A., Schade R. (2022). Update in the treatment of retinopathy of prematurity. Am. J. Perinatol..

[373] Kim S.J., Port A.D., Swan R., Campbell J.P., Chan R.V.P., Chiang M.F. (2018). Retinopathy of prematurity: a review of risk factors and their clinical significance. Surv. Ophthalmol..

[374] Banjac L., Banjac G., Kotur-Stevuljević J., Spasojević-Kalimanovska V., Gojković T., Bogavac-Stanojević N. (2018). PRO-OXIDANTS and antioxidants in retinopathy of prematurity. Acta Clin. Croat..

[375] Yu D.-Y., Cringle S.J., Su E.-N., Yu P.K. (2000). Intraretinal oxygen levels before and after photoreceptor loss in the RCS rat. Invest. Ophthalmol. Vis. Sci..

[376] Padnick-Silver L., Derwent J.J.K., Giuliano E., Narfstrom K., Linsenmeier R.A. (2006). Retinal oxygenation and oxygen metabolism in abyssinian cats with a hereditary retinal degeneration. Invest. Ophthalmol. Vis. Sci..

[377] Rivera J.C., Dabouz R., Noueihed B., Omri S., Tahiri H., Chemtob S. (2017). Ischemic retinopathies: oxidative stress and inflammation. Oxid. Med. Cell. Longev..

[378] Hartnett M.E. (2017). Advances in understanding and management of retinopathy of prematurity. Surv. Ophthalmol..

[379] Graziosi A., Perrotta M., Russo D., Gasparroni G., D’Egidio C., Marinelli B. (2020). Oxidative stress markers and the retinopathy of prematurity. J. Clin. Med..

[380] Buhimschi I.A., Buhimschi C.S., Pupkin M., Weiner C.P. (2003). Beneficial impact of term labor: nonenzymatic antioxidant reserve in the human fetus. Am. J. Obstet. Gynecol..

[381] Beharry K.D., Valencia G.B., Lazzaro D.R., Aranda J.V. (2016). Pharmacologic interventions for the prevention and treatment of retinopathy of prematurity. Semin. Perinatol..

[382] Darlow B.A., Buss H., McGill F., Fletcher L., Graham P., Winterbourn C.C. (2005). Vitamin C supplementation in very preterm infants: a randomised controlled trial. Arch. Dis. Child. Fetal Neonatal Ed..

[383] Parad R.B., Allred E.N., Rosenfeld W.N., Davis J.M. (2012). Reduction of retinopathy of prematurity in extremely low gestational age newborns treated with recombinant human Cu/Zn superoxide dismutase. Neonatology.

[384] Tsang J.K.W., Liu J., Lo A.C.Y. (2019). Vascular and neuronal protection in the developing retina: potential therapeutic targets for retinopathy of prematurity. Int. J. Mol. Sci..

[385] Hellström A., Nilsson A.K., Wackernagel D., Pivodic A., Vanpee M., Sjöbom U. (2021). Effect of enteral lipid supplement on severe retinopathy of prematurity: a randomized clinical trial. JAMA Pediatr..

[386] De Moraes C.G. (2013). Anatomy of the visual pathways. J. Glaucoma.

[387] Selhorst J.B., Chen Y. (2009). The optic nerve. Semin. Neurol..

[388] Wilczek M. (1947). The lamina cribrosa and its nature. Br. J. Ophthalmol..

[389] Quigley H.A., Hohman R.M., Addicks E.M., Massof R.W., Green W.R. (1983). Morphologic changes in the lamina cribrosa correlated with neural loss in open-angle glaucoma. Am. J. Ophthalmol..

[390] Wang L., Dong J., Cull G., Fortune B., Cioffi G.A. (2003). Varicosities of intraretinal ganglion cell axons in human and nonhuman primates. Invest. Ophthalmol. Vis. Sci..

[391] Bristow E.A., Griffiths P.G., Andrews R.M., Johnson M.A., Turnbull D.M. (2002). The distribution of mitochondrial activity in relation to optic nerve structure. Arch. Ophthalmol..

[392] Yu D.Y., Cringle S.J., Balaratnasingam C., Morgan W.H., Yu P.K., Su E.N. (2013). Retinal ganglion cells: energetics, compartmentation, axonal transport, cytoskeletons and vulnerability. Prog. Retin. Eye Res..

[393] Kang E.Y., Liu P.K., Wen Y.T., Quinn P.M.J., Levi S.R., Wang N.K. (2021). Role of oxidative stress in ocular diseases associated with retinal ganglion cells degeneration. Antioxidants (Basel).

[394] Casson R.J., Chidlow G., Wood J.P., Crowston J.G., Goldberg I. (2012). Definition of glaucoma: clinical and experimental concepts. Clin. Exp. Ophthalmol..

[395] Anderson D.R., Patella V.M. (1999). Automated Static Perimetry.

[396] Khazaeni B., Khazaeni L. (2022).

[397] Wright C., Tawfik M.A., Waisbourd M., Katz L.J. (2016). Primary angle-closure glaucoma: an update. Acta Ophthalmol..

[398] Quigley H.A., Broman A.T. (2006). The number of people with glaucoma worldwide in 2010 and 2020. Br. J. Ophthalmol..

[399] Tham Y.C., Li X., Wong T.Y., Quigley H.A., Aung T., Cheng C.Y. (2014). Global prevalence of glaucoma and projections of glaucoma burden through 2040: a systematic review and meta-analysis. Ophthalmology.

[400] Flammer J., Orgül S., Costa V.P., Orzalesi N., Krieglstein G.K., Serra L.M. (2002). The impact of ocular blood flow in glaucoma. Prog. Retin. Eye Res..

[401] Heijl A., Leske M.C., Bengtsson B., Hyman L., Bengtsson B., Hussein M. (2002). Reduction of intraocular pressure and glaucoma progression: results from the Early Manifest Glaucoma Trial. Arch. Ophthalmol..

[402] Garway-Heath D.F., Crabb D.P., Bunce C., Lascaratos G., Amalfitano F., Anand N. (2015). Latanoprost for open-angle glaucoma (UKGTS): a randomised, multicentre, placebo-controlled trial. Lancet.

[403] Anderson D.R. (2003). Collaborative normal tension glaucoma study. Curr. Opin. Ophthalmol..

[404] Nucci C., Di Pierro D., Varesi C., Ciuffoletti E., Russo R., Gentile R. (2013). Increased malondialdehyde concentration and reduced total antioxidant capacity in aqueous humor and blood samples from patients with glaucoma. Mol. Vis..

[405] Zanon-Moreno V., Garcia-Medina J.J., Gallego-Pinazo R., Vinuesa-Silva I., Moreno-Nadal M.A., Pinazo-Duran M.D. (2009). Antioxidant status modifications by topical administration of dorzolamide in primary open-angle glaucoma. Eur. J. Ophthalmol..

[406] Ferreira S.M., Lerner S.F., Brunzini R., Evelson P.A., Llesuy S.F. (2004). Oxidative stress markers in aqueous humor of glaucoma patients. Am. J. Ophthalmol..

[407] Izzotti A., Saccà S.C., Longobardi M., Cartiglia C. (2010). Mitochondrial damage in the trabecular meshwork of patients with glaucoma. Arch. Ophthalmol..

[408] Saccà S.C., Pascotto A., Camicione P., Capris P., Izzotti A. (2005). Oxidative DNA damage in the human trabecular meshwork: clinical correlation in patients with primary open-angle glaucoma. Arch. Ophthalmol..

[409] Abu-Amero K.K., Kondkar A.A., Mousa A., Osman E.A., Al-Obeidan S.A. (2013). Decreased total antioxidants in patients with primary open angle glaucoma. Curr. Eye Res..

[410] Asano Y., Himori N., Kunikata H., Yamazaki M., Shiga Y., Omodaka K. (2017). Age- and sex-dependency of the association between systemic antioxidant potential and glaucomatous damage. Sci. Rep..

[411] Tanito M., Kaidzu S., Takai Y., Ohira A. (2016). Association between systemic oxidative stress and visual field damage in open-angle glaucoma. Sci. Rep..

[412] Tanito M., Kaidzu S., Takai Y., Ohira A. (2015). Correlation between systemic oxidative stress and intraocular pressure level. PLoS One.

[413] Saccà S.C., Izzotti A., Rossi P., Traverso C. (2007). Glaucomatous outflow pathway and oxidative stress. Exp. Eye Res..

[414] Llobet A., Gasull X., Gual A. (2003). Understanding trabecular meshwork physiology: a key to the control of intraocular pressure?. News Physiol. Sci..

[415] Johnson M. (2006). What controls aqueous humour outflow resistance?. Exp. Eye Res..

[416] Hogg P., Calthorpe M., Batterbury M., Grierson I. (2000). Aqueous humor stimulates the migration of human trabecular meshwork cells in vitro. Invest. Ophthalmol. Vis. Sci..

[417] Zhou L., Li Y., Yue B.Y. (1999). Oxidative stress affects cytoskeletal structure and cell-matrix interactions in cells from an ocular tissue: the trabecular meshwork. J. Cell. Physiol..

[418] Gericke A., Mann C., Zadeh J.K., Musayeva A., Wolff I., Wang M. (2019). Elevated intraocular pressure causes abnormal reactivity of mouse retinal arterioles. Oxid. Med. Cell. Longev..

[419] Wang M., Liu H., Xia N., Li H., van Beers T., Gericke A. (2022). Intraocular pressure-induced endothelial dysfunction of retinal blood vessels is persistent, but does not trigger retinal ganglion cell loss. Antioxidants.

[420] Ju W.K., Kim K.Y., Lindsey J.D., Angert M., Duong-Polk K.X., Scott R.T. (2008). Intraocular pressure elevation induces mitochondrial fission and triggers OPA1 release in glaucomatous optic nerve. Invest. Ophthalmol. Vis. Sci..

[421] Tezel G., Wax M.B. (2000). Increased production of tumor necrosis factor-alpha by glial cells exposed to simulated ischemia or elevated hydrostatic pressure induces apoptosis in cocultured retinal ganglion cells. J. Neurosci..

[422] Tezel G. (2008). TNF-alpha signaling in glaucomatous neurodegeneration. Prog. Brain Res..

[423] Mantzaris M.D., Bellou S., Skiada V., Kitsati N., Fotsis T., Galaris D. (2016). Intracellular labile iron determines H2O2-induced apoptotic signaling via sustained activation of ASK1/JNK-p38 axis. Free Radic. Biol. Med..

[424] Harada C., Nakamura K., Namekata K., Okumura A., Mitamura Y., Iizuka Y. (2006). Role of apoptosis signal-regulating kinase 1 in stress-induced neural cell apoptosis in vivo. Am. J. Pathol..

[425] Di Marzo N., Chisci E., Giovannoni R. (2018). The role of hydrogen peroxide in redox-dependent signaling: homeostatic and pathological responses in mammalian cells. Cells.

[426] Kitsati N., Mantzaris M.D., Galaris D. (2016). Hydroxytyrosol inhibits hydrogen peroxide-induced apoptotic signaling via labile iron chelation. Redox Biol..

[427] Chang Y.-S., Chang Y.-C., Chen P.-H., Li C.-Y., Wu W.-C., Kao Y.-H. (2021). MicroRNA-100 mediates hydrogen peroxide-induced apoptosis of human retinal pigment epithelium ARPE-19 cells. Pharmaceuticals.

[428] Tezel G., Wax M.B. (2004). Hypoxia-inducible factor 1alpha in the glaucomatous retina and optic nerve head. Arch. Ophthalmol..

[429] Guillemin K., Krasnow M.A. (1997). The hypoxic response: huffing and HIFing. Cell.

[430] Rupin A., Paysant J., Sansilvestri-Morel P., Lembrez N., Lacoste J.M., Cordi A. (2004). Role of NADPH oxidase-mediated superoxide production in the regulation of E-selectin expression by endothelial cells subjected to anoxia/reoxygenation. Cardiovasc. Res..

[431] Mittal M., Roth M., König P., Hofmann S., Dony E., Goyal P. (2007). Hypoxia-dependent regulation of nonphagocytic NADPH oxidase subunit NOX4 in the pulmonary vasculature. Circ. Res..

[432] Kleikers P.W., Wingler K., Hermans J.J., Diebold I., Altenhöfer S., Radermacher K.A. (2012). NADPH oxidases as a source of oxidative stress and molecular target in ischemia/reperfusion injury. J Mol Med (Berl).

[433] Kietzmann T., Görlach A. (2005). Reactive oxygen species in the control of hypoxia-inducible factor-mediated gene expression. Semin. Cell Dev. Biol..

[434] Rieger J.M., Shah A.R., Gidday J.M. (2002). Ischemia-reperfusion injury of retinal endothelium by cyclooxygenase- and xanthine oxidase-derived superoxide. Exp. Eye Res..

[435] Tezel G., Yang X. (2004). Caspase-independent component of retinal ganglion cell death, in vitro. Invest. Ophthalmol. Vis. Sci..

[436] Laspas P., Zhutdieva M.B., Brochhausen C., Musayeva A., Zadeh J.K., Pfeiffer N. (2019). The M(1) muscarinic acetylcholine receptor subtype is important for retinal neuron survival in aging mice. Sci. Rep..

[437] Ying Y., Xue R., Yang Y., Zhang S.X., Xiao H., Zhu H. (2021). Activation of ATF4 triggers trabecular meshwork cell dysfunction and apoptosis in POAG. Aging (Albany NY).

[438] Kasetti R.B., Patel P.D., Maddineni P., Patil S., Kiehlbauch C., Millar J.C. (2020). ATF4 leads to glaucoma by promoting protein synthesis and ER client protein load. Nat. Commun..

[439] Peters J.C., Bhattacharya S., Clark A.F., Zode G.S. (2015). Increased endoplasmic reticulum stress in human glaucomatous trabecular meshwork cells and tissues. Invest. Ophthalmol. Vis. Sci..

[440] Chai F., Yan H., Zhao X., Li J., Pei C. (2022). The role of GRP78 in oxidative stress induced by tunicamycin in trabecular meshwork cells. Acta Biochim. Pol..

[441] Doh S.H., Kim J.H., Lee K.M., Park H.Y., Park C.K. (2010). Retinal ganglion cell death induced by endoplasmic reticulum stress in a chronic glaucoma model. Brain Res..

[442] Marola O.J., Syc-Mazurek S.B., Libby R.T. (2019). DDIT3 (CHOP) contributes to retinal ganglion cell somal loss but not axonal degeneration in DBA/2J mice. Cell Death Dis..

[443] Sato K., Sato T., Ohno-Oishi M., Ozawa M., Maekawa S., Shiga Y. (2021). CHOP deletion and anti-neuroinflammation treatment with hesperidin synergistically attenuate NMDA retinal injury in mice. Exp. Eye Res..

[444] Lin B., Zhang X., Xu X. (2022). Nerve growth factor protects retinal ganglion cells related to inhibiting endoplasmic reticulum stress by inhibiting IRE1-JNK-CHOP signaling pathway. Ocul. Immunol. Inflamm..

[445] Hiramatsu N., Chiang W.C., Kurt T.D., Sigurdson C.J., Lin J.H. (2015). Multiple mechanisms of unfolded protein response-induced cell death. Am. J. Pathol..

[446] Hurley D.J., Normile C., Irnaten M., O’Brien C. (2022). The intertwined roles of oxidative stress and endoplasmic reticulum stress in glaucoma. Antioxidants (Basel).

[447] Lin J.H., Walter P., Yen T.S.B. (2008). Endoplasmic reticulum stress in disease pathogenesis. Annu. Rev. Pathol..

[448] Bhattarai K.R., Riaz T.A., Kim H.-R., Chae H.-J. (2021). The aftermath of the interplay between the endoplasmic reticulum stress response and redox signaling. Exp. Mol. Med..

[449] Hwang J., Qi L. (2018). Quality control in the endoplasmic reticulum: crosstalk between ERAD and UPR pathways. Trends Biochem. Sci..

[450] Han J., Back S.H., Hur J., Lin Y.H., Gildersleeve R., Shan J. (2013). ER-stress-induced transcriptional regulation increases protein synthesis leading to cell death. Nat. Cell Biol..

[451] Kaneko M., Niinuma Y., Nomura Y. (2003). Activation signal of nuclear factor-κb in response to endoplasmic reticulum stress is transduced via IRE1 and tumor necrosis factor receptor-associated factor 2. Biol. Pharm. Bull..

[452] Chen A.C.-H., Burr L., McGuckin M.A. (2018). Oxidative and endoplasmic reticulum stress in respiratory disease. Clin. Transl. Immunol..

[453] Sciarretta S., Zhai P., Shao D., Zablocki D., Nagarajan N., Terada L.S. (2013). Activation of NADPH oxidase 4 in the endoplasmic reticulum promotes cardiomyocyte autophagy and survival during energy stress through the protein kinase RNA-activated-like endoplasmic reticulum kinase/eukaryotic initiation factor 2α/activating transcription factor 4 pathway. Circ. Res..

[454] Booth D.M., Enyedi B., Geiszt M., Várnai P., Hajnóczky G. (2016). Redox nanodomains are induced by and control calcium signaling at the ER-mitochondrial interface. Mol. Cell.

[455] Liu X.G., Wu S.Q., Li P., Yang H. (2015). Advancement in the chemical analysis and quality control of flavonoid in Ginkgo biloba. J. Pharm. Biomed. Anal..

[456] Yu H., Dong L.-H., Zhang Y., Liu Q. (2022). A network pharmacology-based strategy for predicting the protective mechanism of Ginkgo biloba on damaged retinal ganglion cells. Chin. J. Nat. Med..

[457] Lee D., Shim M.S., Kim K.Y., Noh Y.H., Kim H., Kim S.Y. (2014). Coenzyme Q10 inhibits glutamate excitotoxicity and oxidative stress-mediated mitochondrial alteration in a mouse model of glaucoma. Invest. Ophthalmol. Vis. Sci..

[458] Nelson K.M., Dahlin J.L., Bisson J., Graham J., Pauli G.F., Walters M.A. (2017). The essential medicinal chemistry of curcumin. J. Med. Chem..

[459] Yue Y.K., Mo B., Zhao J., Yu Y.J., Liu L., Yue C.L. (2014). Neuroprotective effect of curcumin against oxidative damage in BV-2 microglia and high intraocular pressure animal model. J. Ocul. Pharmacol. Therapeut..

[460] Buccarello L., Dragotto J., Hassanzadeh K., Maccarone R., Corbo M., Feligioni M. (2021). Retinal ganglion cell loss in an ex vivo mouse model of optic nerve cut is prevented by curcumin treatment. Cell Death Dis..

[461] Galiniak S., Aebisher D., Bartusik-Aebisher D. (2019). Health benefits of resveratrol administration. Acta Biochim. Pol..

[462] Pirhan D., Yüksel N., Emre E., Cengiz A., Kürşat Yıldız D. (2016). Riluzole- and resveratrol-induced delay of retinal ganglion cell death in an experimental model of glaucoma. Curr. Eye Res..

[463] Avotri S., Eatman D., Russell-Randall K. (2019). Effects of resveratrol on inflammatory biomarkers in glaucomatous human trabecular meshwork cells. Nutrients.

[464] Ye M.J., Meng N. (2021). Resveratrol acts via the mitogen-activated protein kinase (MAPK) pathway to protect retinal ganglion cells from apoptosis induced by hydrogen peroxide. Bioengineered.

[465] Kimura A., Guo X., Noro T., Harada C., Tanaka K., Namekata K. (2015). Valproic acid prevents retinal degeneration in a murine model of normal tension glaucoma. Neurosci. Lett..

[466] Zhang Z., Qin X., Zhao X., Tong N., Gong Y., Zhang W. (2012). Valproic acid regulates antioxidant enzymes and prevents ischemia/reperfusion injury in the rat retina. Curr. Eye Res..

[467] Aksar A.T., Yuksel N., Gok M., Cekmen M., Caglar Y. (2015). Neuroprotective effect of edaravone in experimental glaucoma model in rats: a immunofluorescence and biochemical analysis. Int. J. Ophthalmol..

[468] Akaiwa K., Namekata K., Azuchi Y., Guo X., Kimura A., Harada C. (2017). Edaravone suppresses retinal ganglion cell death in a mouse model of normal tension glaucoma. Cell Death Dis..

[469] Masuda T., Shimazawa M., Hara H. (2017). Retinal diseases associated with oxidative stress and the effects of a free radical scavenger (edaravone). Oxid. Med. Cell. Longev..

[470] Wan P., Su W., Zhang Y., Li Z., Deng C., Zhuo Y. (2017). Trimetazidine protects retinal ganglion cells from acute glaucoma via the Nrf2/Ho-1 pathway. Clin. Sci. (Lond.).

[471] Li Y., Wang Q., Chu C., Liu S. (2020). Astaxanthin protects retinal ganglion cells from acute glaucoma via the Nrf2/HO-1 pathway. J. Chem. Neuroanat..

[472] Inman D.M., Lambert W.S., Calkins D.J., Horner P.J. (2013). α-Lipoic acid antioxidant treatment limits glaucoma-related retinal ganglion cell death and dysfunction. PLoS One.

[473] Gao Z., Li M., Yao F., Xia X., Duan T., Meng J. (2022). Valdecoxib protects against cell apoptosis induced by endoplasmic reticulum stress via the inhibition of PERK-ATF4-CHOP pathway in experimental glaucoma. Int. J. Mol. Sci..

[474] Roda M., di Geronimo N., Pellegrini M., Schiavi C. (2020). Nutritional optic neuropathies: state of the art and emerging evidences. Nutrients.

[475] Yu-Wai-Man P., Turnbull D.M., Chinnery P.F. (2002). Leber hereditary optic neuropathy. J. Med. Genet..

[476] Yu-Wai-Man P., Griffiths P.G., Brown D.T., Howell N., Turnbull D.M., Chinnery P.F. (2003). The epidemiology of Leber hereditary optic neuropathy in the North East of England. Am. J. Hum. Genet..

[477] Mascialino B., Leinonen M., Meier T. (2012). Meta-analysis of the prevalence of Leber hereditary optic neuropathy mtDNA mutations in Europe. Eur. J. Ophthalmol..

[478] Rosenberg T., Nørby S., Schwartz M., Saillard J., Magalhães P.J., Leroy D. (2016). Prevalence and genetics of leber hereditary optic neuropathy in the Danish population. Invest. Ophthalmol. Vis. Sci..

[479] Puomila A., Hämäläinen P., Kivioja S., Savontaus M.L., Koivumäki S., Huoponen K. (2007). Epidemiology and penetrance of Leber hereditary optic neuropathy in Finland. Eur. J. Hum. Genet..

[480] Spruijt L., Kolbach D.N., de Coo R.F., Plomp A.S., Bauer N.J., Smeets H.J. (2006). Influence of mutation type on clinical expression of Leber hereditary optic neuropathy. Am. J. Ophthalmol..

[481] Man P.Y.W., Turnbull D.M., Chinnery P.F. (2002). Leber hereditary optic neuropathy. J. Med. Genet..

[482] Stramkauskaitė A., Povilaitytė I., Glebauskienė B., Liutkevičienė R. (2022). Clinical overview of leber hereditary optic neuropathy. Acta Med. Litu..

[483] Nikoskelainen E., Hoyt W.F., Nummelin K. (1983). Ophthalmoscopic findings in Leber’s hereditary optic neuropathy. II. The fundus findings in the affected family members. Arch. Ophthalmol..

[484] Harvey J.P., Sladen P.E., Yu-Wai-Man P., Cheetham M.E. (2022). Induced pluripotent stem cells for inherited optic neuropathies-disease modeling and therapeutic development. J. Neuro Ophthalmol..

[485] Carelli V., Rugolo M., Sgarbi G., Ghelli A., Zanna C., Baracca A. (2004). Bioenergetics shapes cellular death pathways in Leber’s hereditary optic neuropathy: a model of mitochondrial neurodegeneration. Biochim. Biophys. Acta Bioenerg..

[486] Yen M.-Y., Lee J.-F., Liu J.-H., Wei Y.-H. (1998). Energy charge is not decreased in lymphocytes of patients with Leber’s hereditary optic neuropathy with the 11,778 mutation. J. Neuro Ophthalmol.: Off. J. North Am. Neuro-ophthalmol. Soc..

[487] Cock H., Cooper J., Schapira A. (1999). Functional consequences of the 3460-bp mitochondrial DNA mutation associated with Leber’s hereditary optic neuropathy. J. Neurol. Sci..

[488] Yen M.-Y., Wang A.-G., Wei Y.-H. (2006). Leber’s hereditary optic neuropathy: a multifactorial disease. Prog. Retin. Eye Res..

[489] Pitkanen S., Robinson B.H. (1996). Mitochondrial complex I deficiency leads to increased production of superoxide radicals and induction of superoxide dismutase. J. Clin. Invest..

[490] Lin C.S., Sharpley M.S., Fan W., Waymire K.G., Sadun A.A., Carelli V. (2012). Mouse mtDNA mutant model of Leber hereditary optic neuropathy. Proc. Natl. Acad. Sci. USA.

[491] Yen M.Y., Kao S.H., Wang A.G., Wei Y.H. (2004). Increased 8-hydroxy-2'-deoxyguanosine in leukocyte DNA in Leber’s hereditary optic neuropathy. Invest. Ophthalmol. Vis. Sci..

[492] Ghelli A., Porcelli A.M., Zanna C., Martinuzzi A., Carelli V., Rugolo M. (2008). Protection against oxidant-induced apoptosis by exogenous glutathione in Leber hereditary optic neuropathy cybrids. Invest. Ophthalmol. Vis. Sci..

[493] Floreani M., Napoli E., Martinuzzi A., Pantano G., De Riva V., Trevisan R. (2005). Antioxidant defences in cybrids harboring mtDNA mutations associated with Leber’s hereditary optic neuropathy. FEBS J..

[494] Lin C.S., Sharpley M.S., Fan W., Waymire K.G., Sadun A.A., Carelli V. (2012). Mouse mtDNA mutant model of Leber hereditary optic neuropathy. Proc. Natl. Acad. Sci. U.S.A..

[495] Kirches E. (2011). LHON: mitochondrial mutations and more. Curr. Genom..

[496] Qi X., Sun L., Lewin A.S., Hauswirth W.W., Guy J. (2007). The mutant human ND4 subunit of complex I induces optic neuropathy in the mouse. Invest. Ophthalmol. Vis. Sci..

[497] Barrientos A., Moraes C.T. (1999). Titrating the effects of mitochondrial complex I impairment in the cell physiology. J. Biol. Chem..

[498] Porcelli A.M., Angelin A., Ghelli A., Mariani E., Martinuzzi A., Carelli V. (2009). Respiratory complex I dysfunction due to mitochondrial DNA mutations shifts the voltage threshold for opening of the permeability transition pore toward resting levels. J. Biol. Chem..

[499] Ghelli A., Zanna C., Porcelli A.M., Schapira A.H.V., Martinuzzi A., Carelli V. (2003). Leber’s hereditary optic neuropathy (LHON) pathogenic mutations induce mitochondrial-dependent apoptotic death in transmitochondrial cells incubated with galactose medium. J. Biol. Chem..

[500] Danielson S.R., Wong A., Carelli V., Martinuzzi A., Schapira A.H.V., Cortopassi G.A. (2002). Cells bearing mutations causing Leber’s hereditary optic neuropathy are sensitized to fas-induced apoptosis. J. Biol. Chem..

[501] Zanna C., Ghelli A., Porcelli A.M., Martinuzzi A., Carelli V., Rugolo M. (2005). Caspase-independent death of Leber’s hereditary optic neuropathy cybrids is driven by energetic failure and mediated by AIF and Endonuclease G. Apoptosis.

[502] Hage R., Vignal-Clermont C. (2021). Leber hereditary optic neuropathy: review of treatment and management. Front. Neurol..

[503] Lyseng-Williamson K.A., Idebenone (2016). A review in Leber’s hereditary optic neuropathy. Drugs.

[504] Gueven N., Ravishankar P., Eri R., Rybalka E. (2021). Idebenone: when an antioxidant is not an antioxidant. Redox Biol..

[505] Gueven N., Woolley K., Smith J. (2015). Border between natural product and drug: comparison of the related benzoquinones idebenone and coenzyme Q10. Redox Biol..

[506] Giorgio V., Petronilli V., Ghelli A., Carelli V., Rugolo M., Lenaz G. (2012). The effects of idebenone on mitochondrial bioenergetics. Biochim. Biophys. Acta.

[507] Haroon M.F., Fatima A., Schöler S., Gieseler A., Horn T.F.W., Kirches E. (2007). Minocycline, a possible neuroprotective agent in Leber’s hereditary optic neuropathy (LHON): studies of cybrid cells bearing 11778 mutation. Neurobiol. Dis..

[508] Ghelli A., Porcelli A.M., Zanna C., Martinuzzi A., Carelli V., Rugolo M. (2008). Protection against oxidant-induced apoptosis by exogenous glutathione in leber hereditary optic neuropathy cybrids. Invest. Ophthalmol. Vis. Sci..

[509] Ng W.S.V., Trigano M., Freeman T., Varrichio C., Kandaswamy D.K., Newland B. (2021). New avenues for therapy in mitochondrial optic neuropathies. Ther. Adv. Respir. Dis..

[510] Sadun A.A., Chicani C.F., Ross-Cisneros F.N., Barboni P., Thoolen M., Shrader W.D. (2012). Effect of EPI-743 on the clinical course of the mitochondrial disease Leber hereditary optic neuropathy. Arch. Neurol..

[511] Chicani C., Chu E., Ross-Cisneros F., Rockwell S., Murase K., Thoolen M. (2013). Treatment of Leber’s hereditary optic neuropathy (LHON): results using a novel quinone, EPI-743. Invest. Ophthalmol. Vis. Sci..

[512] Pitceathly R.D.S., Keshavan N., Rahman J., Rahman S. (2021). Moving towards clinical trials for mitochondrial diseases. J. Inherit. Metab. Dis..

[513] Chen M., Liu B., Ma J., Ge J., Wang K. (2017). Protective effect of mitochondria-targeted peptide MTP-131 against oxidative stress-induced apoptosis in RGC-5 cells. Mol. Med. Rep..

[514] Seo K.S., Kim J.H., Min K.N., Moon J.A., Roh T.C., Lee M.J. (2018). KL1333, a novel NAD(+) modulator, improves energy metabolism and mitochondrial dysfunction in MELAS fibroblasts. Front. Neurol..

[515] Patel H.R., Margo C.E. (2017). Pathology of ischemic optic neuropathy. Arch. Pathol. Lab Med..

[516] Farina N., Tomelleri A., Campochiaro C., Dagna L. (2023). Giant cell arteritis: update on clinical manifestations, diagnosis, and management. Eur. J. Intern. Med..

[517] Hayreh S.S. (2021). Giant cell arteritis: its ophthalmic manifestations. Indian J. Ophthalmol..

[518] Winkler A., True D. (2018). Giant cell arteritis: 2018 review. Mo. Med..

[519] Ninan J., Lester S., Hill C. (2016). Giant cell arteritis. Best Pract. Res. Clin. Rheumatol..

[520] Mackie S.L., Dejaco C., Appenzeller S., Camellino D., Duftner C., Gonzalez-Chiappe S. (2020). British Society for Rheumatology guideline on diagnosis and treatment of giant cell arteritis. Rheumatology (Oxford).

[521] Lyons H.S., Quick V., Sinclair A.J., Nagaraju S., Mollan S.P. (2020). A new era for giant cell arteritis. Eye.

[522] Berry S., Lin W.V., Sadaka A., Lee A.G. (2017). Nonarteritic anterior ischemic optic neuropathy: cause, effect, and management. Eye Brain.

[523] Patil A.D., Biousse V., Newman N.J. (2022). Ischemic optic neuropathies: current concepts. Ann. Indian Acad. Neurol..

[524] Liu B., Yu Y., Liu W., Deng T., Xiang D. (2021). Risk factors for non-arteritic anterior ischemic optic neuropathy: a large scale meta-analysis. Front. Med..

[525] Hayreh S.S., Zimmerman M.B., Podhajsky P., Alward W.L.M. (1994). Nocturnal arterial hypotension and its role in optic nerve head and ocular ischemic disorders. Am. J. Ophthalmol..

[526] Hayreh S.S. (2009). Ischemic optic neuropathy. Prog. Retin. Eye Res..

[527] Espino Barros A., Amram A.L., Derham A.M., Smith S.V., Lee A.G. (2017). Management of ischemic optic neuropathies. Expet Rev. Ophthalmol..

[528] Hayreh S.S., Zimmerman B. (2005). Visual field abnormalities in nonarteritic anterior ischemic optic neuropathy: their pattern and prevalence at initial examination. Arch. Ophthalmol..

[529] Katz D.M., Trobe J.D. (2015). Is there treatment for nonarteritic anterior ischemic optic neuropathy. Curr. Opin. Ophthalmol..

[530] Watanabe R., Hashimoto M. (2022). Aging-related vascular inflammation: giant cell arteritis and neurological disorders. Front. Aging Neurosci..

[531] Bilton E.J., Mollan S.P. (2023). Giant cell arteritis: reviewing the advancing diagnostics and management. Eye.

[532] Larsson K., Mellström D., Nordborg E., Odén A., Nordborg E. (2006). Early menopause, low body mass index, and smoking are independent risk factors for developing giant cell arteritis. Ann. Rheum. Dis..

[533] Weyand C.M., Goronzy J.J. (2014). Clinical practice. Giant-cell arteritis and polymyalgia rheumatica. N. Engl. J. Med..

[534] Li L., Neogi T., Jick S. (2017). Giant cell arteritis and vascular disease-risk factors and outcomes: a cohort study using UK Clinical Practice Research Datalink. Rheumatology (Oxford).

[535] Tyrrell D.J., Blin M.G., Song J., Wood S.C., Zhang M., Beard D.A. (2020). Age-associated mitochondrial dysfunction accelerates atherogenesis. Circ. Res..

[536] Ungvari Z., Tarantini S., Donato A.J., Galvan V., Csiszar A. (2018). Mechanisms of vascular aging. Circ. Res..

[537] Ianni A., Kumari P., Tarighi S., Argento F.R., Fini E., Emmi G. (2021). An insight into giant cell arteritis pathogenesis: evidence for oxidative stress and SIRT1 downregulation. Antioxidants (Basel).

[538] Wang L., Ai Z., Khoyratty T., Zec K., Eames H.L., van Grinsven E. (2020). ROS-producing immature neutrophils in giant cell arteritis are linked to vascular pathologies. JCI Insight.

[539] Ophir A., Berenshtein E., Kitrossky N., Berman E.R., Photiou S., Rothman Z. (1993). Hydroxyl radical generation in the cat retina during reperfusion following ischemia. Exp. Eye Res..

[540] Piantadosi C.A., Zhang J. (1996). Mitochondrial generation of reactive oxygen species after brain ischemia in the rat. Stroke.

[541] Osborne N.N., Casson R.J., Wood J.P.M., Chidlow G., Graham M., Melena J. (2004). Retinal ischemia: mechanisms of damage and potential therapeutic strategies. Prog. Retin. Eye Res..

[542] Li C., Jackson R.M. (2002). Reactive species mechanisms of cellular hypoxia-reoxygenation injury. Am. J. Physiol. Cell Physiol..

[543] Wan W., Peng T., Jin X., Li Q., Zhang F., Zheng G. (2016). Glutathione-S-Transferase deletions and non-arteritic anterior ischemic optic neuropathy. Mol. Neurobiol..

[544] Abu-Amero K.K., Milcarek B., Bosley T.M. (2009). GSTM1 and GSTT1 deletion genotypes in various spontaneous optic neuropathies in Arabs. Br. J. Ophthalmol..

[545] Bosley T.M., Abu-Amero K.K., Ozand P.T. (2004). Mitochondrial DNA nucleotide changes in non-arteritic ischemic optic neuropathy. Neurology.

[546] Birer S., Arda H., Kilic D., Baskol G. (2019). Systemic oxidative stress in non-arteritic anterior ischemic optic neuropathy. Eye (Lond)..

[547] Xie J., Zhang X., Zhang L. (2013). Negative regulation of inflammation by SIRT1. Pharmacol. Res..

[548] Singh C.K., Chhabra G., Ndiaye M.A., Garcia-Peterson L.M., Mack N.J., Ahmad N. (2018). The role of sirtuins in antioxidant and redox signaling. Antioxidants Redox Signal..

[549] Fang C.E.H., Guo L., Hill D., Yap T.E., Cordeiro M.F. (2020). Neuroprotective strategies in glaucoma - translation to clinical trials. OBM Neurobiol..

[550] Wubben T.J., Besirli C.G., Johnson M.W., Zacks D.N. (2018). Retinal neuroprotection: overcoming the translational roadblocks. Am. J. Ophthalmol..

[551] Almasieh M., Levin L.A. (2017). Neuroprotection in glaucoma: animal models and clinical trials. Ann. Rev. Vision Sci..

[552] Ashok A., Andrabi S.S., Mansoor S., Kuang Y., Kwon B.K., Labhasetwar V. (2022). Antioxidant therapy in oxidative stress-induced neurodegenerative diseases: role of nanoparticle-based drug delivery systems in clinical translation. Antioxidants.

[553] Hill D., Compagnoni C., Cordeiro M.F. (2021). Investigational neuroprotective compounds in clinical trials for retinal disease. Expet Opin. Invest. Drugs.

[554] Normando E.M., Yap T.E., Maddison J., Miodragovic S., Bonetti P., Almonte M. (2020). A CNN-aided method to predict glaucoma progression using DARC (Detection of Apoptosing Retinal Cells). Expert Rev. Mol. Diagn..

[555] Bhatt D.L., Mehta C. (2016). Adaptive designs for clinical trials. N. Engl. J. Med..

