# Supplementary material for: Oxidative stress in the eye and its role in the pathophysiology of ocular diseases
Source: Redox Biol. 2023 Nov 18;68:102967. doi: 10.1016/j.redox.2023.102967 (PMC10701459; doi:10.1016/j.redox.2023.102967)
Supplement: Multimedia component 1 [file mmc1.docx]

**Online Supplement**

#### **Table 1.** Therapeutic approaches for oxidative stress in dry eye disease.

| **Antioxidant agent** | **Species/**  **Animal model** | **Application** | **Effect** | **Reference** |
| --- | --- | --- | --- | --- |
| Vitamin A (retinyl palmitate) and cyclosporine A 0,05% | Human | Topical | Improvement in blurred vision, tear film BUT and Schirmer I score results | [1] |
| Vitamin A (retinol palmitate) | Rabbits with lacrimal gland resection | Topical | Recovery of the corneal epithelium, increase in density of conjunctival goblet cells | [2] |
| Vitamin A | Human | Oral versus intramuscular | Effective treatment of severe xerophthalmia | [3] |
| Vitamin B12 | Human | Nebulization | Elevation of BUT, higher basal epithelial cell density | [4] |
| Coenzyme Q10 and vitamin E | Human | Topical | Faster nerve regeneration, better stability of ocular surface | [5] |
| Vitamin C and vitamin E | Human | Oral | Higher goblet cell density; reduced stage of metaplasia; improved values for Schirmer test, BUT and ocular ferning | [6] |
| Vitamin D3 | Mouse model of Sjögren’s syndrome | Oral | Preservation of the corneal epithelium morphology and thickness, reduced levels and activity of corneal TNF-α | [7] |
| Epigallocatechin gallate (polyphenol) | Mouse | Topical | Decreased corneal fluorescein staining, decreased inflammatory markers (interleukin-1β, chemokine ligand 2, VEGF-A, VEGF-D) | [8] |
| Green tea extract | Human | Topical | Improved score of clinical symptoms, improved BUT, improved health of Meibomian glands | [9] |
| Alpha-lipoic acid | Ovariectomized rats | Oral | Altered metabolism of RNS, increased activity of lacrimal peroxidase, improved lacrimal production | [10] |
| Omega-3 fatty acids | Human | Topical | Improved clinical signs of DED (total corneal staining, OSDI, BUT, visual analog scales) | [11] |
| Hyaluronic acid 0.15 % and vitamin B12 | Human | Topical | Reduced levels of oxidative stress, reduced OSDI and FCT scores, increased Schirmer’s test and BUT scores | [12] |

**Table 2.** Therapeutic approaches targeting oxidative stress in glaucoma.

| **Antioxidant agent** | **Species/**  **Animal model** | **Application** | **Effect** | **Reference** |
| --- | --- | --- | --- | --- |
| Spermidine | Mouse model of NTG | Oral | Ameliorated retinal degeneration, improved visual function, antioxidant effects by reduced increase of 4-HNE | [13] |
| Spermidine | Mouse model of optic nerve injury | Oral | Inhibition of the ROS-mediated ASK-1/p38/MAPK apoptotic axis, inhibition of iNOS expression in microglia, decreased production of chemokines | [14] |
| Rapamycin | Rat model with chronic ocular hypertension | Intraperitoneal | Direct suppression of apoptosis in retinal ganglion cells, inhibition of the production of NO and TNF-α, modulation of NF-kB signaling | [15] |
| Brimonidine | Rats with induced intraocular hypertension | Topical | Reversed oxidative stress created by high intraocular pressure | [16] |
| N-acetyl cysteine | Rat model with induced ocular hypertension | Intraperitoneal | Reversed oxidative stress created by high intraocular pressure | [16] |
| N-acetyl cysteine | Mouse model of NTG | Intraperitoneal | Prevention of RGC degeneration and visual impairment, suppression of oxidative stress and autophagy | [17] |
| Geranylgeranylacetone | Mouse model of NTG | Oral | Suppression of RGC loss, reduced activity of caspase-9 and -3 | [18] |
| Valproic acid | Human | Oral | Improved visual acuity | [19] |
| Nicotinamide (vitamin B3) | Human | Oral | Improvement of inner retinal functions, improved visual field mean deviation | [20] |
| Nicotinamide | Rat models of glaucoma | Oral | RGC neuroprotection, prevention of metabolic disruption, prevention of metabolic stress | [21] |
| EPO-R76E | Mouse model of glaucoma | Intravitreal | Prevention of increased retinal superoxide levels, phosphorylation of Nrf2, upregulation of antioxidants | [22] |
| Phenylbutyric acid (PBA) | Mouse model of glaucoma | Oral | Reduced ER stress, prevention of TM cell death | [23] |
| Sodium-4-phenylbutyrate (PBA) | Mouse model of ocular hypertension | Topical | Decreased glucocorticoid-induced elevated intraocular pressure, degradation of abnormal extracellular matrix in TM by induction of MMP9 | [24] |

**Table 3.** Therapeutic approaches targeting oxidative stress in non-arteritic anterior ischemic optic neuropathy.

| **Antioxidant agent** | **Species/**  **Animal model** | **Application** | **Effect** | **Reference** |
| --- | --- | --- | --- | --- |
| Astaxanthin | Rat model of ischemic optic nerve | Oral | Higher expression of antioxidants (Nrf2, SOD), reduced RGC apoptosis, preservation of visual functions | [25] |
| Resveratrol | Rat model of I/R injury | Intraperitoneal | Reduced retinal damage and RGC apoptosis, reversion of downregulation of Opa1 and reduced SOD activity | [26] |
| Resveratrol | Mouse model of I/R injury | Oral | Preservation of vascular endothelial function and autoregulation, reduced ROS/RNS formation, reduced expression of NOX2 | [27] |
| Geranylgeranylacetone | Mouse model of I/R injury | Oral | Increased number of surviving retinal neurons, suppression of apoptosis in RGC | [28] |
| N-butylidenephthalide (BP) | Rat model of NAION | Intraperitoneal | Improved RGC survival, prevention of apoptotic processes, reduced inflammatory response by inhibition of NF-κB pathway | [29] |
| Vitamin B3 | Rat model of rAION | Oral | Higher amplitudes of flash visual-evoked potentials and RGC densities, decreased levels of ROS, reduction in apoptotic cells | [30] |
| Oroxylin A | Rat model of rAION | Subcutaneous | Reduced optic disc edema, reduced apoptosis of RGC, reduced infiltration of inflammatory cells, activation of Nrf2 and downstream antioxidant enzymes, preservation of visual function | [31] |
| Betulinic acid | Mouse model of I/R injury | Oral | Prevention of vascular endothelial dysfunction, reduced ROS formation, increased expression of antioxidant enzymes | [32] |
| E2121 (ROCK inhibitor) | Rat model of ischemic optic neuropathy | Intravitreal | Increased activity of SOD, decreased RGC-produced ROS levels, reduced number of apoptotic RGCs and macrophages | [33] |

**References**

1 Kim EC, Choi JS, Joo CK. A comparison of vitamin a and cyclosporine a 0.05% eye drops for treatment of dry eye syndrome. Am J Ophthalmol. **2009**;147(2):206-13.e3. doi: 10.1016/j.ajo.2008.08.015

2 Odaka A, Toshida H, Ohta T, Tabuchi N, Koike D, Suto C, et al. Efficacy of retinol palmitate eye drops for dry eye in rabbits with lacrimal gland resection. Clin Ophthalmol. **2012**;6:1585-93. doi: 10.2147/opth.s35584

3 Sommer A, Muhilal, Tarwotjo I, Djunaedi E, Glover J. Oral versus intramuscular vitamin A in the treatment of xerophthalmia. Lancet. **1980**;1(8168 Pt 1):557-9. doi: 10.1016/s0140-6736(80)91053-3

4 Yang J, Liu Y, Xu Y, Li X, Fu J, Jiang X, et al. A new approach of ocular nebulization with vitamin B12 versus oxytocin for the treatment of dry eye disease: an in vivo confocal microscopy study. Drug Des Devel Ther. **2019**;13:2381-91. doi: 10.2147/dddt.s203464

5 Fogagnolo P, Sacchi M, Ceresara G, Paderni R, Lapadula P, Orzalesi N, et al. The effects of topical coenzyme Q10 and vitamin E D-α-tocopheryl polyethylene glycol 1000 succinate after cataract surgery: a clinical and in vivo confocal study. Ophthalmologica. **2013**;229(1):26-31. doi: 10.1159/000342196

6 Peponis V, Bonovas S, Kapranou A, Peponi E, Filioussi K, Magkou C, et al. Conjunctival and tear film changes after vitamin C and E administration in non-insulin dependent diabetes mellitus. Med Sci Monit. **2004**;10(5):Cr213-7. doi:

7 Trotta MC, Herman H, Balta C, Rosu M, Ciceu A, Mladin B, et al. Oral Administration of Vitamin D3 Prevents Corneal Damage in a Knock-Out Mouse Model of Sjögren's Syndrome. Biomedicines. **2023**;11(2). doi: 10.3390/biomedicines11020616

8 Lee HS, Chauhan SK, Okanobo A, Nallasamy N, Dana R. Therapeutic efficacy of topical epigallocatechin gallate in murine dry eye. Cornea. **2011**;30(12):1465-72. doi: 10.1097/ICO.0b013e31821c9b5a

9 Nejabat M, Reza SA, Zadmehr M, Yasemi M, Sobhani Z. Efficacy of Green Tea Extract for Treatment of Dry Eye and Meibomian Gland Dysfunction; A Double-blind Randomized Controlled Clinical Trial Study. J Clin Diagn Res. **2017**;11(2):Nc05-nc8. doi: 10.7860/jcdr/2017/23336.9426

10 Andrade AS, Salomon TB, Behling CS, Mahl CD, Hackenhaar FS, Putti J, et al. Alpha-lipoic acid restores tear production in an animal model of dry eye. Exp Eye Res. **2014**;120:1-9. doi: 10.1016/j.exer.2013.12.014

11 Jacobi C, Angstmann-Mehr S, Lange A, Kaercher T. A Water-Free Omega-3 Fatty Acid Eye Drop Formulation for the Treatment of Evaporative Dry Eye Disease: A Prospective, Multicenter Noninterventional Study. J Ocul Pharmacol Ther. **2022**;38(5):348-53. doi: 10.1089/jop.2021.0102

12 Macri A, Scanarotti C, Bassi AM, Giuffrida S, Sangalli G, Traverso CE, et al. Evaluation of oxidative stress levels in the conjunctival epithelium of patients with or without dry eye, and dry eye patients treated with preservative-free hyaluronic acid 0.15 % and vitamin B12 eye drops. Graefes Arch Clin Exp Ophthalmol. **2015**;253(3):425-30. doi: 10.1007/s00417-014-2853-6

13 Noro T, Namekata K, Azuchi Y, Kimura A, Guo X, Harada C, et al. Spermidine Ameliorates Neurodegeneration in a Mouse Model of Normal Tension Glaucoma. Invest Ophthalmol Vis Sci. **2015**;56(8):5012-9. doi: 10.1167/iovs.15-17142

14 Noro T, Namekata K, Kimura A, Guo X, Azuchi Y, Harada C, et al. Spermidine promotes retinal ganglion cell survival and optic nerve regeneration in adult mice following optic nerve injury. Cell Death Dis. **2015**;6(4):e1720. doi: 10.1038/cddis.2015.93

15 Su W, Li Z, Jia Y, Zhuo Y. Rapamycin is neuroprotective in a rat chronic hypertensive glaucoma model. PLoS One. **2014**;9(6):e99719. doi: 10.1371/journal.pone.0099719

16 Ozdemir G, Tolun FI, Gul M, Imrek S. Retinal Oxidative Stress Induced by Intraocular Hypertension in Rats May be Ameliorated by Brimonidine Treatment and N-acetyl Cysteine Supplementation. Journal of Glaucoma. **2009**;18(9):662-5. doi: 10.1097/IJG.0b013e31819c46b1

17 Sano H, Namekata K, Kimura A, Shitara H, Guo X, Harada C, et al. Differential effects of N-acetylcysteine on retinal degeneration in two mouse models of normal tension glaucoma. Cell Death Dis. **2019**;10(2):75. doi: 10.1038/s41419-019-1365-z

18 Dong Z, Shinmei Y, Dong Y, Inafuku S, Fukuhara J, Ando R, et al. Effect of geranylgeranylacetone on the protection of retinal ganglion cells in a mouse model of normal tension glaucoma. Heliyon. **2016**;2(10):e00191. doi: 10.1016/j.heliyon.2016.e00191

19 Mahalingam K, Chaurasia AK, Gowtham L, Gupta S, Somarajan BI, Velpandian T, et al. Therapeutic potential of valproic acid in advanced glaucoma: A pilot study. Indian J Ophthalmol. **2018**;66(8):1104-8. doi: 10.4103/ijo.IJO_108_18

20 Hui F, Tang J, Williams PA, McGuinness MB, Hadoux X, Casson RJ, et al. Improvement in inner retinal function in glaucoma with nicotinamide (vitamin B3) supplementation: A crossover randomized clinical trial. Clin Exp Ophthalmol. **2020**;48(7):903-14. doi: 10.1111/ceo.13818

21 Tribble JR, Otmani A, Sun S, Ellis SA, Cimaglia G, Vohra R, et al. Nicotinamide provides neuroprotection in glaucoma by protecting against mitochondrial and metabolic dysfunction. Redox Biol. **2021**;43:101988. doi: 10.1016/j.redox.2021.101988

22 Naguib S, DeJulius CR, Backstrom JR, Haider AA, Ang JM, Boal AM, et al. Intraocular Sustained Release of EPO-R76E Mitigates Glaucoma Pathogenesis by Activating the NRF2/ARE Pathway. Antioxidants (Basel). **2023**;12(3). doi: 10.3390/antiox12030556

23 Zode GS, Kuehn MH, Nishimura DY, Searby CC, Mohan K, Grozdanic SD, et al. Reduction of ER stress via a chemical chaperone prevents disease phenotypes in a mouse model of primary open angle glaucoma. J Clin Invest. **2011**;121(9):3542-53. doi: 10.1172/jci58183

24 Maddineni P, Kasetti RB, Kodati B, Yacoub S, Zode GS. Sodium 4-Phenylbutyrate Reduces Ocular Hypertension by Degrading Extracellular Matrix Deposition via Activation of MMP9. Int J Mol Sci. **2021**;22(18). doi: 10.3390/ijms221810095

25 Lin WN, Kapupara K, Wen YT, Chen YH, Pan IH, Tsai RK. Haematococcus pluvialis-Derived Astaxanthin Is a Potential Neuroprotective Agent against Optic Nerve Ischemia. Mar Drugs. **2020**;18(2). doi: 10.3390/md18020085

26 Pang Y, Qin M, Hu P, Ji K, Xiao R, Sun N, et al. Resveratrol protects retinal ganglion cells against ischemia induced damage by increasing Opa1 expression. Int J Mol Med. **2020**;46(5):1707-20. doi: 10.3892/ijmm.2020.4711

27 Chronopoulos P, Manicam C, Zadeh JK, Laspas P, Unkrig JC, Göbel ML, et al. Effects of Resveratrol on Vascular Function in Retinal Ischemia-Reperfusion Injury. Antioxidants (Basel). **2023**;12(4). doi: 10.3390/antiox12040853

28 Harada C, Nakamura K, Guo X, Kitaichi N, Mitamura Y, Yoshida K, et al. Neuroprotective effect of geranylgeranylacetone against ischemia-induced retinal injury. Mol Vis. **2007**;13:1601-7. doi:

29 Chou YY, Chien JY, Ciou JW, Huang SP. The Protective Effects of n-Butylidenephthalide on Retinal Ganglion Cells during Ischemic Injury. Int J Mol Sci. **2022**;23(4). doi: 10.3390/ijms23042095

30 Chen T-W, Wu P-Y, Wen Y-T, Desai TD, Huang C-T, Liu P-K, et al. Vitamin B3 Provides Neuroprotection via Antioxidative Stress in a Rat Model of Anterior Ischemic Optic Neuropathy. Antioxidants. **2022**;11(12):2422. doi:

31 Chien JY, Lin SF, Chou YY, Huang CF, Huang SP. Protective Effects of Oroxylin A on Retinal Ganglion Cells in Experimental Model of Anterior Ischemic Optic Neuropathy. Antioxidants (Basel). **2021**;10(6). doi: 10.3390/antiox10060902

32 Musayeva A, Unkrig JC, Zhutdieva MB, Manicam C, Ruan Y, Laspas P, et al. Betulinic Acid Protects from Ischemia-Reperfusion Injury in the Mouse Retina. Cells. **2021**;10(9). doi: 10.3390/cells10092440

33 Wen Y-T, Huang C-W, Liu C-P, Chen C-H, Tu C-M, Hwang C-S, et al. Inhibition of Retinal Ganglion Cell Loss By a Novel ROCK Inhibitor (E212) in Ischemic Optic Nerve Injury Via Antioxidative and Anti-Inflammatory Actions. Investigative Ophthalmology & Visual Science. **2021**;62(6):21-. doi: 10.1167/iovs.62.6.21
